# A revision of the new world species of *Polytrichophora* Cresson and *Facitrichophora*, new genus (Diptera, Ephydridae)

**DOI:** 10.3897/zookeys.231.3687

**Published:** 2012-10-24

**Authors:** Wayne N. Mathis, Tadeusz Zatwarnicki

**Affiliations:** 1 Department of Entomology, NHB 169, PO Box 37012; Smithsonian Institution, Washington, D.C. 20013-7012, USA; 2 Department of Biosystematics, Opole University, ul. Oleska 22, 45-052 Opole, Poland

**Keywords:** Diptera, Ephydridae, Gymnymyzinae, Discocerinini, *Facitrichophora*, *Polytrichophora*, New World, new species

## Abstract

The New World species of *Polytrichophora* Cresson and *Facitrichophora* new genus, are revised. Fifteen new species are described (type locality in parenthesis): *Facitrichophora atrella* **sp. n.** (Costa Rica. Guanacaste: Murciélago [10°56.9'N, 85°42.5'W; sandy mud flats around mangrove inlet]), *Facitrichophora carvalhorum* **sp. n.** (Brazil. São Paulo: Praia Puruba [23°21'S, 44°55.6'W; beach]), *Facitrichophora manza* **sp. n.** (Trinidad and Tobago. Trinidad. St. Andrew: Lower Manzanilla (12 km S; 10°24.5'N, 61°01.5'W), bridge over Nariva River), *Facitrichophora panama* **sp. n.** (Panama. Darien: Garachine [8°04'N, 78°22'W]), *Polytrichophora adarca* **sp. n.** (Barbados. Christ Church: Graeme Hall Nature Sanctuary [13°04.2'N, 59°34.7'W; swamp]), *Polytrichophora arnaudorum* **sp. n.** (Mexico. Baja California. San Felipe [31°01.5'N, 114°50.4'W]), *Polytrichophora barba* **sp. n.** (Cuba. Sancti Spiritus: Topes de Collantes [21°54.4'N, 80°01.4'W, 670 m]), *Polytrichophora flavella* **sp. n.** (Peru. Madre de Dios: Rio Manu, Pakitza [11°56.6'S, 71°16.9'W; 250 m]), *Polytrichophora marinoniorum* **sp. n.** (Brazil. Paraná: Antonina [25°28.4'S, 48°40.9'W; mangal]), *Polytrichophora rostra* **sp. n.** (Peru. Madre de Dios: Rio Manu, Pakitza [11°56.6'S, 71°16.9'W; 250 m]), *Polytrichophora sinuosa* **sp. n.** (Trinidad and Tobago. Trinidad. St. Andrew: Lower Manzanilla [12 km S; 10°24'N, 61°02'W]), *Polytrichophora mimbres* **sp. n.** (United States. New Mexico. Grant: Mimbres River [New Mexico Highway 61 & Royal John Mine Road; 32°43.8'N, 107°52'W; 1665 m]), *Polytrichophora salix* **sp. n.** (United States. Alaska. Matanuska-Susitna: Willow Creek [61°46.1'N, 150°04.2'W; 50 m]), *Polytrichophora sturtevantorum* **sp. n.** (United States. Tennessee. Shelby: Meeman Shelby State Park [Mississippi River; 35°20.4'N, 90°2.1'W; 98 m]), *Polytrichophora prolata* **sp. n.** (Belize. Stann Creek: Cockscomb Basin Wildlife Sanctuary [16°45'N, 88°30'W]). All known New World species of both genera are described with an emphasis on structures of the male terminalia, which are fully illustrated. Detailed locality data and distribution maps for all species are provided. For perspective and to facilitate recognition, the tribe Discocerinini is diagnosed and a key to included genera is provided.

## Introduction

*Polytrichophora* Cresson occurs worldwide and now comprises 31 species, including 11 undescribed species that we treat in this revision of the New World fauna of this genus. Previously on a worldwide basis, 20 species were known (Mathis and Zatwarnicki 1995 and updates). We focus on the New World fauna, which for many years has been woefully in need of comprehensive revision. Cresson (1942, 1946) produced the last synopses of *Polytrichophora* for the Americas (North America and South America respectively) near the end of his distinguished research career on shore flies, but his series of synopses are now 70 years old and quite inadequate for recognizing the vast influx of newly discovered species. In his two synopses, for example, Cresson treated four species in the Nearctic Region (*Polytrichophora agens* Cresson, *Polytrichophora conciliata* Cresson, *Polytrichophora orbitalis* (Loew), *Polytrichophora setigera* (Cresson)) and three species in the Neotropical Region (*Polytrichophora desmata* (Williston), *Polytrichophora pulchra* Cresson, *Polytrichophora setulosa* (Cresson)) with no apparent overlap of species between these two regions. Between Cresson and this revision, Mathis (1997a) described the only other New World congener, *Polytrichophora reginae*, in a faunistic study of the shore flies of the Belizean Cays. Thus, the New World fauna, based on species reported in this revision, has increased dramatically from eight to 19 species, 11 being undescribed, which further documents the need for revising this biologically diverse genus.

Much of this increase has resulted from a greatly improved sampling of the New World fauna, especially the neotropics where the first author has focused much of his fieldwork for nearly four decades. Also contributing to this increase is our use of characters from structures of the male terminalia, which has revealed species complexes for what had been treated in some cases as a single, widespread species. The seven new species of the *conciliata* group are an example of this discovery process and taxonomic evolution.

In addition of a revision of *Polytrichophora*, we are also revising *Facitrichophora*, a new genus that includes four species, all undescribed, and that is related to *Polytrichophora* and other genera of the *Discocerina* group. The four species that we are describing in this genus all occur in the New World tropics.

Why study *Polytrichophora* or *Facitrichophora*? As alluded to previously, the New World fauna of *Polytrichophora* exhibits considerable diversity, and until this revision, most of the components of this diversity, the species, were unknown or had been misidentified. A basic component of this or any biodiversity study is a comprehensive, taxonomic treatment of the fauna wherein all included species are fully elaborated, including how they are differentiated (recognized), where they occur, the relevant literature, and a summary of their natural history, as this information is available. The importance of knowing a species’ scientific name cannot be overly emphasized, as it provides an entrée into the literature on that species for those seeking further information (Thompson 1997).

Another reason for this study is to contribute to our understanding of the evolutionary and phylogenetic relationships among the included taxa. Discovering the complexity of a species group, for example, or determining the phylogenetic signal of character evidence that we discover and document, contribute significantly to clarifying the evolutionary history of the group, which in turn, becomes the backbone for other comparative, biological studies, such as behavioral studies or those on the historical biogeography of a group. Knowing the phylogeny of a group provides the necessary evolutionary context for other studies in comparative biology.

We know virtually nothing about the natural history of any species of *Polytrichophora* or *Facitrichophora* (see “Natural History” under generic heading, p. 26) and another objective of revision is to stimulate work on this invaluable component--understanding the natural history of this genus.

We cannot overly emphasize, however, that the primary stimulus and reason for this revision was to satisfy our own innate curiosity about these shore flies. The motivation generated by the process of discovery is compelling and greatly contributed to this study. Curiosity and discovery are basic to human nature and ought to be supported and otherwise fostered.

The taxonomic and nomenclatural history of the New World species of *Polytrichophora* is both short and simple, especially as there were relatively few species and contributors involved. Loew (1861) described the first New World species, *Polytrichophora orbitalis* (Loew), in the genus *Discocerina* and then Cresson transferred it to *Polytrichophora* (Cresson 1924) when he first proposed *Polytrichophora* as a genus-group taxon. That same sequence of description in another genus then transfer to *Polytrichophora* was followed by the next three species now included in the genus: (1) Williston (1896) described *Polytrichophora desmata* (Williston) in the genus *Psilopa* and then Mathis and Edmiston (1991) transferred it to *Polytrichophora*; (2) Cresson (1916) described *Polytrichophora setigera* (Cresson) in *Discocerina* and then transferred it to *Polytrichophora* (Cresson 1924); and (3) Cresson (1918) described *Polytrichophora setulosa* in *Discocerina* and then transferred it to *Polytrichophora* (Cresson 1924). The other previously described, New World species were all originally described as congeners in *Polytrichophora*: Cresson (1924) described *Polytrichophora agens*, *Polytrichophora conciliata*, and *Polytrichophora pulchra* and Mathis (1997b), in a review of the shore flies of Belizean Cays, described *Polytrichophora reginae*. The only other New World species proposed is *Polytrichophora boriqueni* Cresson (1930) from specimens collected on Puerto Rico. Cresson's species was later discovered to be conspecific with *Polytrichophora desmata*. The latter species had been described 34 years earlier in *Psilopa*, a genus in the subfamily Discomyzinae, not Gymnomyzinae, which is the subfamily in which genera of the tribe Discocerinini are currently placed (Mathis and Zatwarnicki 1995).

Virtually nothing is known about the natural history of *Polytrichophora* except for brief descriptions of habitats where adults have been collected (Deonier 1965), and these, perhaps, are suspect because we now know that three species occur in the Midwest (so far as we have records, however, only *Polytrichophora sturtevantorum* is currently known to occur in Iowa).

## Methods and materials

The methods used generally in this study were explained previously (Mathis 1986, Mathis and Zatwarnicki 1990a). Because specimens are small, usually less than 3.5 mm in length, study and illustration of the male terminalia required use of a compound microscope.

**Figures 1–4.**
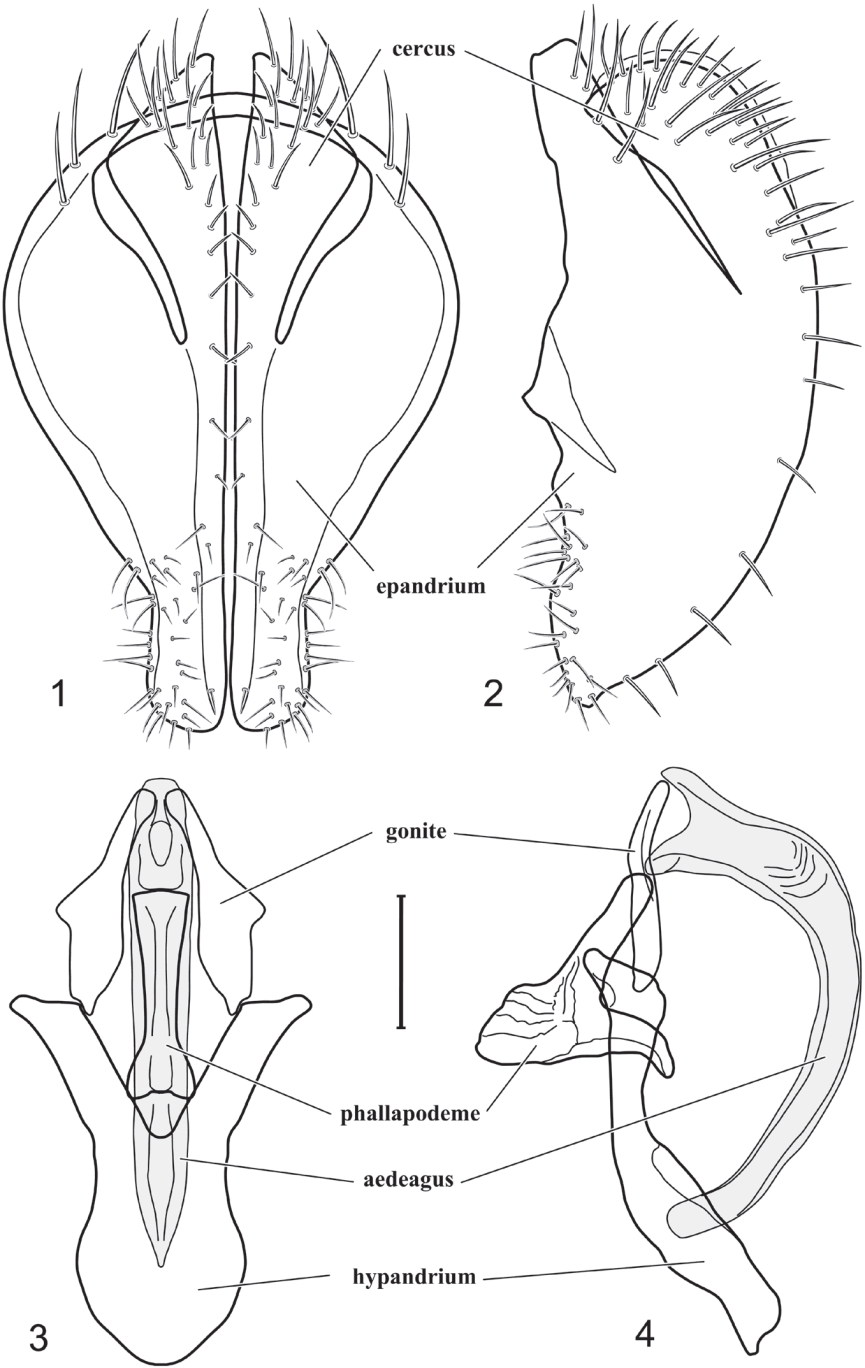
Illustration of *Facitrichophora atrella* sp. n. (male) (Costa Rica. Guanacaste: Nandaynre, Playa Blanca) **1** epandrium and cerci, posterior view **2** same, lateral view **3** internal structures of male terminalia (aedeagus [shaded], phallapodeme, gonite, hypandrium), ventral view **4** same, lateral view. Scale bar = 0.1 mm.

We have followed the terminology for most structures of the male terminalia that other workers in Ephydridae have used (see references in Mathis 1986, and Mathis and Zatwarnicki 1990a, 1990b), and the terminology for these structures provided directly on Figs 1–4 (*Facitrichophora atrella* sp. n.) and is not repeated for comparable illustrations of other species.

Dissections of male terminalia were performed following Clausen and Cook (1971) and Grimaldi (1987). Abdomens were removed with microforceps and macerated in a sodium hydroxide solution. Cleared genitalia were then transferred to glycerin for observation, description, and illustration. The dissected abdomen was placed in a plastic microvial filled with glycerin and attached to the pin supporting the remainder of the insect from which it had been removed.

Three ratios (two are venational) are used commonly in the descriptions and are defined here for the convenience of the user (ratios are averages of three specimens).

1. Gena-to-Eye ratio: Genal height/eye height. Measurements are taken from the head in lateral view.

2. Costal vein ratio: The straight line distance between the apices of R_2+3_ and R_4+5_/distance between the apices of R_1_ and R_2+3_.

3. M vein ratio: The straight line distance along vein M between crossveins (r-m and dm-cu)/distance apicad of crossvein dm-cu.

Distribution maps were made using ESRI ArcView© GIS 3.2. Longitude and latitude coordinates were obtained for the locality where each specimen was collected and entered into a Microsoft Excel© spreadsheet. If unavailable directly from specimen labels, longitude and latitude were estimated using gazetteers and maps to determine the geographical coordinates. Localities of specimens were plotted on a world land projection, presented within ESRI ArcView layouts and exported as encapsulated postscript (EPS) files.

Although we do not include a rigorous cladistic analysis as part of this revision, we do provide some discussion on the phylogeny of some groups, especially the *conciliata* group. We include character evidence, usually synapomorphies, for these groupings, and it is understood that this evidence is putative and further, that the phylogenetic inferences are hypotheses. We have not done a cladistic analysis in part because *Polytrichophora* is a worldwide genus and herein we treat only the New World fauna. In the Old World there are numerous species, including some that are undescribed. At the generic level, the discussions of phylogeny provided here are written within the perspective of an earlier paper on the phylogeny of Discocerinini (Zatwarnicki and Mathis 2001).

Although this study was based in large part on specimens in the National Museum of Natural History (USNM), numerous others were borrowed, particularly type specimens of the species previously described. To our colleagues and their institutions listed below who loaned specimens, we express our sincere thanks. Without their cooperation this study could not have been completed.

AMNHAmerican Museum of Natural History, New York, New York (David A. Grimaldi and Julian Stark)

ANSPAcademy of Natural Sciences of Philadelphia, Pennsylvania (Jon K. Gelhaus and Jason D. Weintraub)

CMNHCarnegie Museum of Natural History, Pittsburgh, Pennsylvania (Chen Young and John E. Rawlins)

DZUPUniversidade Federal do Paraná, Coleção Entomológica Padre Jesus Santiago Moure, Departamento de Zoologia, Curitiba, Paraná, Brazil (Luciane Marinoni)

INBIOInstituto Nacional de Biodiversidad, Santo Domingo, Heredia, Costa Rica (Manuel A. Zumbado)

INPAInstituto Nacional de Pesquisas da Amazônia, Manaus, Amazonas, Brazil (José Albertino Rafael)

MCZMuseum of Comparative Zoology, Harvard University, Cambridge, Massachusetts (Philip D. Perkins)

MZUSPMuseu de Zoologia da Universidade de São Paulo, São Paulo, Brazil (Carlos José Einicker Lamas)

## Systematics

### Tribe Discocerinini

Cresson

Discocerinini (as Discocerini) Cresson 1925: 228. Type genus: *Discocerina* Macquart 1835. Mathis and Zuyin 1989: 435 [description, key to Asian genera]. Zatwarnicki and Mathis 2001: 1–51 [revision of genera, phylogeny].

#### Diagnosis.

A tribe of the subfamily Gymnymyzinae that is distinguished by the following combination of characters: Small to medium-sized shore flies, body length 1.25–3.50 mm; usually invested with considerable microtomentum, especially frons and mesonotum.

*Head*: Frontal vitta (or ocellar triangle) mostly bare of setulae, not conspicuously setulose; pseudopostocellar setae well developed, length greater than distance between either posterior ocellus and anterior ocellus, generally with proclinate orientation and slightly divergent; ocellar seta inserted anterior to lateral alignment of anterior ocellus, sometimes only slightly so; reclinate fronto-orbital seta inserted in front of proclinate fronto-orbital (if 2 proclinate fronto-orbital setae present, reclinate seta inserted in front of the larger proclinate seta); proclinate fronto-orbital seta subequal to length of reclinate seta. Pedicel bearing a large seta anterodorsally; arista with 5–7 dorsally branching rays evenly along aristal length. Compound eye bearing numerous, interfacetal microsetulae. Face generally smooth, not conspicuously pitted or rugose, in lateral view shallowly carinate between antennal bases and/or very shallowly conically produced, convex. Gena generally short (secondarily high in some species), bearing setulae (including midportion) and 1 large seta, its posterior (postgenal) margin rounded, not sharp. Oral opening and clypeus narrow; mouthparts generally dark colored; clypeus generally microtomentose, similar to microtomentum of face.

*Thorax*: Mesonotum generally microtomentose, usually densely so; supra-alar seta usually evident although sometimes reduced; acrostichal setulae arranged in about 8 irregular rows; prescutellar acrostichal setae approximate and inserted behind level of posteromost dorsocentral setae; scutellum usually moderately densely setulose, bearing more than 20 setulae, these evenly scattered; both anterior and posterior notopleural setae inserted at about the same level from notopleural/anepisternal suture; anepisternum with 2 equal setae along posterior margin. Wing with vein R_2+3_ long, extended nearly to level of apex of vein R_4+5_. Foreleg normally developed, not raptorial with greatly enlarged femur.

*Abdomen*: Five tergites visible, usually not covered with microtomentum. Male terminalia: Structures symmetrical; cerci paired, hemispherical, setose, bearing sides of rectum, sometimes fused with posteroventral margin of epandrium; epandrium U-shaped, encircling cerci, anterior margin rounded, in lateral view with setae mainly on dorsum and along anteroventral margin; presurstylus lacking or fused indistinguishably with epandrium; posterolateral arms of epandrium attached with ventral apex of gonites, middle of posterior margin a base for phallapodeme; phallapodeme situated under aedeagus, associated with hypandrium and with ventral part of base of aedeagus, ventral margin with lobate appendix providing attachment for genital muscles that move aedeagus, sometimes fused with base of aedeagus; gonites paired, connecting sides of base of aedeagus and laterodorsal margin of epandrium, bearing 1 or some setulae; subepandrial plate reduced; aedeagus tubular, tapered anteriorly; ejaculatory apodeme usually lacking, if present as a spatula against background of ductus ejaculatorius.

**Discussion.** Starting with Cresson (1925), who first described Discocerinini, and including all students of the family until Mathis and Zuyin (1989), the diagnoses, descriptions, and catalogs of this tribe included some taxa that are not closely related phylogenetically, rendering the tribe polyphyletic. Mathis and Zuyin (1989) recharacterized Discocerinini using synapomorphies and resulting in a monophyletic tribe under which Mathis and Zatwarnicki (1995) included eight genera and 143 species in their world catalog. Zatwarnicki and Mathis (2001) added two additional genera, *Galaterina* and *Orasiopa*, and altered the status of some subgenera in their phylogenetic study of the tribe.

#### Phylogenetic relationships.

On a worldwide basis, Zatwarnicki and Mathis (2001) proposed a phylogenetic hypothesis for higher-level lineages within the tribe Discocerinini and the discussion to follow is written within the context of the hypothesis and supportive evidence they then proposed. Discocerinini, according to their proposed phylogeny, is divided into three sublineages: the *Gymnoclasiopa*, *Diclasiopa*, and *Discocerina* groups. Other genera in addition to *Facitrichophora* and *Polytrichophora* that are included in the *Discocerina* group are: *Discocerina* Macquart, *Galaterina* Zatwarnicki and Mathis, *Hydrochasma* Hendel, *Lamproclasiopa* Hendel, and *Orasiopa* Zatwarnicki and Mathis. *Facitrichophora* and *Polytrichophora* along with other genera of the *Discocerina* group form a monophyletic lineage within the Discocerinini that is corroborated thus far by two synapomorphies that we have identified. The first is the setulose notopleuron. In other genera of the subfamily Gymnomyzinae, including many in the tribe Discocerinini, the notopleuron is bare except for a larger anterior and a posterior setae that are inserted near the ventral margin of the notopleuron. In taxa of the *Discocerina* group, however, the notopleuron bears a few additional setulae that are usually inserted slightly dorsad and toward the anterior portion of the notopleuron, usually around the anterior notopleural seta. The second synapomorphy confirming the monophyly of the *Discocerina* group is the shape of the gonite, which is narrowly elongate, bar-like, and often essentially parallel sided. In other genera of Discocerinini outside of the *Discocerina* group, the gonite is elongate, variously swollen medially, and tapered toward both apices. Within the *Discocerina* group, an elongated male terminalia (hypopygium) that is at least 2.5× longer than wide (the plesiomorphic condition is for the hypopygium to be of moderate length) occurs almost exclusively in three genera: *Galaterina*, *Hydrochasma*, and *Polytrichophora*. The genera *Hydrochasma* and *Polytrichophora* are characterized by a deeply incised posterior margin of the hypandrium (the plesiomorphic condition is a hypandrium with a moderately concave posterior margin). *Polytrichophora* and *Facitrichophora* are distinguished from other New World genera by the characters provided in the key and generic diagnoses that follow (characters being discussed are synapomorphies unless specified otherwise (Zatwarnicki and Mathis 2001)).

#### Key to new world genera of Discocerinini

**Table:** 

1	Notopleuron bare of setulae	2
–	Notopleuron setulose in addition to 2 large setae	6
2	Forefemur slightly enlarged, bearing distinct row of stout, short setae along apical half of posteroventral surface	*Pectinifer* Cresson
–	Forefemur normally developed, lacking row of short, stout setae along posteroventral surface	3
3	Postsutural supra-alar seta strong, distinct, longer than posterior notopleural seta. Face with dorsoclinate seta at lower lateral extremity	*Diclasiopa* Hendel
–	Postsutural supra-alar seta very short or absent, if distinguishable distinctly shorter than posterior notopleural seta. Face without dorsoclinate seta at lower lateral extremity	4
4	Hindtibia with a preapical, ventral, spur-like seta; facial series comprising 2–3 large setae, dorsal seta inserted slightly medially from other setae and arising from distinct, shiny papilla, with a small, slightly dorsoclinate seta laterad of dorsal seta; generally microtomentose, cinereous species, appearing dull	*Hecamedoides* Hendel
–	Hindtibia lacking a preapical, ventral spur-like seta; facial series comprised of 2 large setae, dorsal seta not arising from a shiny papilla and lacking a smaller seta laterad of dorsal seta; mostly bare to sparsely microtomentose, shiny to subshiny species	5
5	Face rather flattened, antennal grooves not always sharply defined ventrally; facial series of setae inserted very close to parafacial, dorsalmost seta not appreciably more removed mesad than ventral seta	*Gymnoclasiopa* Hendel
–	Face rather prominent at level of dorsal facial setae, sometimes transversely carinate: antennal grooves generally sharply defined ventrally	*Ditrichophora* Cresson
6	Face with 2 or more conspicuous rows of setae/setulae on each side, paralleling facial suture setal row medial, row(s) of setulae between setal row and parafacial (Figs 10–11, 28–29)	7
–	Face with a single row of setae laterally	8
7	Face with secondary series of dorsolaterally inclined setae laterad to primary series (Figs 28–29)	*Polytrichophora* Cresson
–	Face with setae and setulae of rows inclinate or ventroinclinate (Figs 10–11)	*Facitrichophora* gen. n.
8	Gena and lower part of parafacial broad; lateral margin of abdomen usually with gray to whitish microtomentose areas, these usually wedge shaped	*Hydrochasma* Hendel
–	Gena and parafacial rather narrow; abdomen lacking wedge-shaped, light-colored areas laterally	9
9	Parafacial wide and bearing setulae	*Discocerina* Macquart
–	Parafacial narrow and lacking setulae	10
10	Postsutural supra-alar and prescutellar acrostichal setae greatly reduced or lacking; facial series of setae 2, these well separated, distance between them subequal to length of basal flagellomere; parafacial very narrow at anteroventral margin of eye	*Lamproclasiopa* Hendel
–	Postsutural supra-alar and prescutellar acrostichal setae present; facial series of setae 3-4, distance between setae conspicuously less than length of basal flagellomere, if 2 facial setae present, see first character; parafacial evenly wide throughout length	*Orasiopa* Zatwarnicki and Mathis

### Genus Facitrichophora gen. n.

urn:lsid:zoobank.org:act:2DD0A279-EB93-4EAC-81C4-33C8B798E41B

http://species-id.net/wiki/Facitrichophora

#### Type species.

*Facitrichophora manza* sp. n., by present designation.

#### Diagnosis.

**Figure 5.**
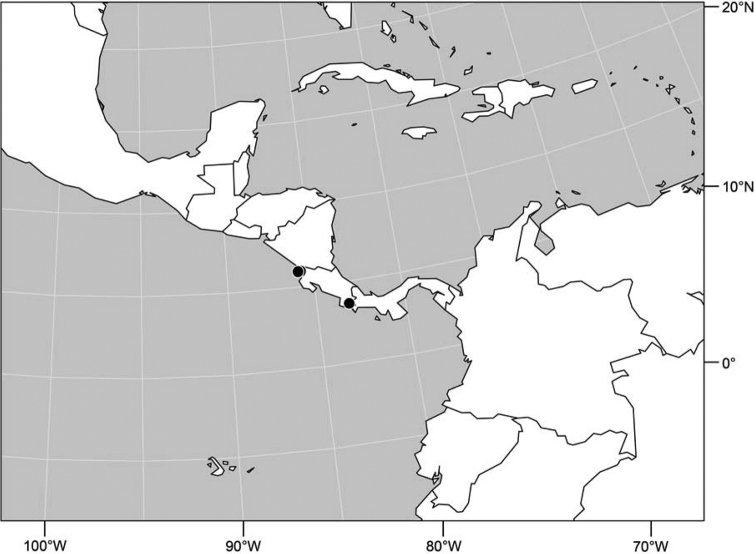
Distribution of *Facitrichophora atrella* sp. n.

Small to moderately small shore flies, length 1.8–3.20 mm; generally densely microtomentose, dull species (Figs 11–13). *Head* (Figs 11–12): Frons lacking anterior, proclinate, fronto-orbital seta; face substantially prominent at level of dorsal facial seta; antennal grooves generally sharply defined ventrally; facial setae comprising at least 2 vertical to verticolateral rows (Figs 11–12), 5 setae of medial row larger than those of lateral row, these not arising from shiny papilla and with similar, mostly inclinate to ventroinclinate orientations; face lacking a distinctly dorsoclinate seta at ventral extremity; parafacial moderately wide, bearing conspicuous, vertical row of large setulae; gena variable but moderately high. Eye generally oval, conspicuously microsetulose, bearing numerous interfacetal setulae; maxillary palpus yellow. *Thorax* (Fig. 13): Acrostichal setulae in 6–8 rows; a presutural and postsutural supra-alar setae moderately well developed, both about 1/2 length of postalar seta; notopleuron bearing several setulae in addition to 2 larger setae; anterior notopleural seta inserted conspicuously closer to posterior notopleural seta than to postpronotal seta; anepisternum bearing 2 prominent, subequal setae along posterior margin. Wings faintly infuscate, more so toward anterior margin; costa bearing 3–5 long, dorsal setae between humeral and subcostal breaks. Forefemur normally developed, lacking row of short, stout setae along anteroventral or posteroventral surfaces; hindtibia lacking a preapical, ventral, spur-like seta. *Abdomen*: Tergites variable, but mostly unicolorous; tergite 2–4 of ♂ becoming progressively longer. Male terminalia (Figs 1–4): Epandrium thinly connected dorsally above cerci, in posterior view more or less elliptical, 2× longer than wide, in ventral half gradually tapered, or pyriform, broader basally and tapered irregularly to apex, on whole surface bearing distinct setae, in lateral view apical half slightly widen by extension of its ventral portion, apical section of ventral margin often with setae; cerci fused ventrolaterally to medial margin of epandrium, in posterior view generally slightly elongate or hemispherical; gonites rod-shaped without setae, rarely lobate; aedeagus strongly elongate, about 4–6× longer than wide, in dorsal view base generally narrowly triangular, apex rounded, in lateral view trunk of aedeagus with uniform thickness, apex pointed and generally bearing subapically a narrow, membranous flap that generally folds back under aedeagus with about 2/3 length of aedeagus to battered tape 2× as long as aedeagus; phallapodeme separate from aedeagus, in posterior view elongate with extension at base and bifurcate anteriorly, in lateral view narrowly subtriangular, with extension toward aedeagal base narrow, parallel sided or tapered, keel usually evident toward hypandrial extension, but sometimes reduced; ejaculatory apodeme absent; hypandrium in dorsal view U- or V- to Y-shaped with thickened stem or base, anterior margin narrowly to broadly rounded, posterior arms either straight or oriented posterolaterally, if straight then with a lateral and medial pair of arms, in lateral view hypandrium generally flattened or broadly and shallowly pocket-like.

#### Distribution.

Species of *Facitrichophora* have only been found in the Neotropical Region.

#### Natural history.

Specimens of *Facitrichophora* are usually associated with mud-shore and sand-shore habitats associated with brackish water in backwater areas of maritime coasts.

#### Discussion.

*Facitrichophora*, along with *Discocerina*, *Hydrochasma* Hendel (New World), and *Polytrichophora*, form a monophyletic lineage within the tribe Discocerinini (see Zatwarnicki and Mathis 2001 for background on this node). *Facitrichophora* may be distinguished from *Discocerina*, *Hydrochasma*, or *Polytrichophora* by the two series of facial setae (see generic description and Figs 11–12) with an inclinate to ventroinclinate orientation; medial facial series larger and numbering 5. These characters are unique to the species of this genus (synapomorphies) and are evidence of its monophyly. *Facitrichophora* is perhaps most closely related to *Polytrichophora* and a synapomorphy establishing this relationship is the fusion of the ventral portion of the cerci with the epandrium.

Four species have been discovered in this genus thus far, and each can be distinguished by external characters. Reference to characters of the male terminalia, however, may be needed to confirm a species’ identity.

#### Key to species of *Facitrichophora*

**Table:** 

1	Two posterior dorsocentral setae, anterior seta slightly smaller than posterior seta (Argentina, Brazil, Costa Rica)	*Facitrichophora carvalhorum* sp. n.
–	One posterior dorsocentral seta	2
2	Mesonotum mostly shiny black with very sparse investment of grayish microtomentum; scutellum bearing several lateral setae, especially posteriorly (Costa Rica)	*Facitrichophora atrella* sp. n.
–	Mesonotum grayish brown to bronzish brown; scutellum with 2 lateral setae	3
3	Tergite 4 of male long, conspicuously longer than tergite 3 and subequal to tergite 5; tergite 3 with ventrolateral margin produced into a curved, narrow, parallel-sided process; tergite 5 with anteroventral angle projected slightly, acutely pointed (Costa Rica, Cuba, Mexico, Trinidad and Tobago)	*Facitrichophora manza* sp. n.
–	Tergites 3-4 of male subequal, 4 not unusually longer, tergite 3 not produced ventrolaterally; tergite 5 rounded or angulate, but not projected anteroventrally and acutely pointed (Panama)	*Facitrichophora panama* sp. n.

### Facitrichophora atrella sp. n.

urn:lsid:zoobank.org:act:95992E47-E1BD-4EC1-8156-1AEAEB992AD2

http://species-id.net/wiki/Facitrichophora_atrella

Figs 1 5

#### Diagnosis.

This species is distinguished from congeners by the following combination of characters: Generally black with tannish gray to silvery face and yellowish tarsomeres. Moderately small shore flies, body length 2.15–2.70 mm.

*Head*: Frons mostly black, generally very sparsely grayish microtomentose, becoming denser and tannish on and around ocellar triangle; fronto-orbits with silvery gray microtomentum. Antennal scape and pedicel black; basal flagellomere black or becoming tannish to brownish apically; arista bearing 5 dorsal rays. Face wide, microtomentum dull to subshiny, tannish gray to silvery. Parafacial concolorous with fronto-orbit. Gena moderately high, height equal to length of scape; gena-to-eye height ratio 0.20–0.28.

*Thorax*: Mesonotum subshiny to shiny, black, with sparse, whitish to grayish microtomentum; a single pair of posterior dorsocentral setae; no evident prescutellar acrostichal setae; scutellum trapezoidal in dorsal view with posterior margin truncate, lateral margins very slightly bowed, bearing 2 primary scutellar setae along lateral margins but also with several larger setae between primary setae, especially posteriorly. Wing uniformly faintly infuscate; costal vein ratio 0.57–0.60; M vein ratio 0.60–0.62.

*Abdomen*: Length of tergites 3 subequal to that of 2, tergite 4 slightly longer than 3, tergite 5 conspicuously longer than 4, triangular. Male terminalia (Figs 1–5): Epandrium narrowly connected dorsally above cerci (Figs 1–2), in posterior view (Fig. 1) pyriform or as an inverted pear, broadly oval on dorsal 3/4, bulged laterally at midheight, dorsal margin broadly rounded, ventral ¼ bilobed, as 2 straight, abutting, parallel-sided, moderately wide processes, width slightly less than that of cerci, each process bearing numerous small setulae, in lateral view (Fig. 2) epandrium and cerci together generally semicircular, cerci prominent, setulose, epandrium robustly developed, posterior margin arched, anterior margin irregularly straight with a shallow, pointed projection at midheight and shallowly sinuous; aedeagus simple, in lateral view (Fig. 4) elongate, narrow, tapered from base to apex, conspicuously curved, more so toward base, apex narrowly and bluntly rounded, in ventral view (Fig. 3) elongate, mostly parallel sided, tapered basally and apically, apex acutely pointed; phallapodeme in lateral view (Fig. 4) with distinct keel, keel angulate, both apices tapered, apex toward aedeagal base longer, parallel sided, digitiform, end toward hypandrium curved, tapered, in ventral view (Fig. 3) bar-like with lateral margins concave, gradually expanded toward each end, hypandrial end more abruptly expanded; gonite in lateral view (Fig. 4) rod-like, elongate, slender, shallowly sinuous, in ventral view (Fig. 3) relatively wide toward hypandrium, opposite end narrowed, digitiform; hypandrium in lateral view (Fig. 4) elongate, narrow, shallowly curved, narrowest at midlength, in ventral view (Fig. 3) more or less V-shaped with base well developed, wide, anterior margin rounded, each lateral arm oriented posterolaterally, robustly developed, apices truncate.

#### Type material.

The holotype male is labeled “COSTA RICA[,] Guanacaste Prov. Murciélago [10°56.9'N, 85°42.5'W;][,] 1 April 1988/Swept from sandy mud flats around mangrove inlet/W. E. Steiner[,] J. M. Hill[,] J. M. Swearingen[,] J. M. Mitchell/USNM ENT 00285972 [plastic bar code label]/HOLOTYPE ♂ *Facitrichophora atrella* Mathis & Zatwarnicki, USNM [red].” The holotype is double mounted (glued to a paper triangle), is in good condition (left wing partially torn), and is deposited in the USNM. Four paratypes (4♀; USNM) bear the same label data as the holotype. Other paratypes are as follows: COSTA RICA. **Guanacaste:** Nandayure, Puerto Thiel (10°01.6'N, 85°11.9'W; 0–20 m), 17 Oct 2003, W. Porras (2♂, 3♀; INBIO, USNM).

#### Other specimens examined.

COSTA RICA. **Puntarenas:** R. V. S. Golfito, Golfito, Llano Bonito (8°38.4'N, 83°11'W; mangal), 28 Apr 2004, W. Porras (2♀; INBIO).

#### Type locality.

Costa Rica. Guanacaste: Murciélago (10°56.9'N, 85°42.5'W; sandy mud flats around mangrove inlet).

#### Distribution

(Fig. 5). Neotropical: Costa Rica (Guanacaste, Puntarenas).

#### Etymology.

The species epithet, *atrella*, is of Latin derivation and refers to the blackish external appearance of this species.

#### Remarks.

Among congeners with a single posterior dorsocentral seta, this species is distinguished externally in having a mostly shiny black mesonotum that is very sparsely invested with grayish microtomentum. In addition, the scutellum has several lateral setae, especially posteriorly.

### Facitrichophora carvalhorum sp. n.

urn:lsid:zoobank.org:act:B47B4F3E-DF71-4922-8A90-FAE44F314160

http://species-id.net/wiki/Facitrichophora_carvalhorum

Figs 6 10

#### Diagnosis.

This species is distinguished from congeners by the following combination of characters: Small to moderately small shore flies, body length 1.80–2.80 mm.

*Head*: Frons dull, moderately to heavily microtomentose, 3-toned, mesofrons mostly tan with some faint golden reflections, more grayish anteriorly and on ocellar tubercle; parafrons black with gray microtomentum, more grayish anteriorly; fronto-orbits silvery gray microtomentum. Antenna short, mostly yellow to yellowish orange, basal flagellomere very short, length subequal to height of pedicle, slightly darker, more brownish yellow; arista with 5 dorsal rays. Face at narrowest point greater than antennal length; face densely microtomentose, seriaceus, silvery white; parafacial and gena similar to face; parafacial with moderate ventral dilation; parafacial 2–3 times wider ventrally than dorsally; gena relatively short, less than 1/4 eye height; eye-to-cheek ratio 0.20–0.22.

*Thorax*: Mesonotum mostly dull to subshiny, moderately densely microtomentose, gray to grayish brown anterolaterally, otherwise brown; no evident prescutellar acrostichal setae; 2 posterior dorsocentral setae, anterior seta slightly smaller; pleural area brownish gray, duller than mesonotum; scutellum triangular in dorsal view, posterior apex pointed; bearing 2 larger lateral setae and lacking larger setae between primary setae. Wing generally infumate, light to dark brown; anterior margin of wing lacking spine-like setae; costal vein ratio 0.53–0.64; M vein 0.50–0.69. Forefemur lacking row of 9–10 short, stout setae along apical half of anteroventral surface; forefemur with a row of 7–9, longer, evenly spaced posteroventral setae, each seta subequal to width of femur; foretibia yellow, mid- and hindtibiae darkened medially, apices yellow; tarsi yellow, apical 1–2 brown.

**Figures 6–9.**
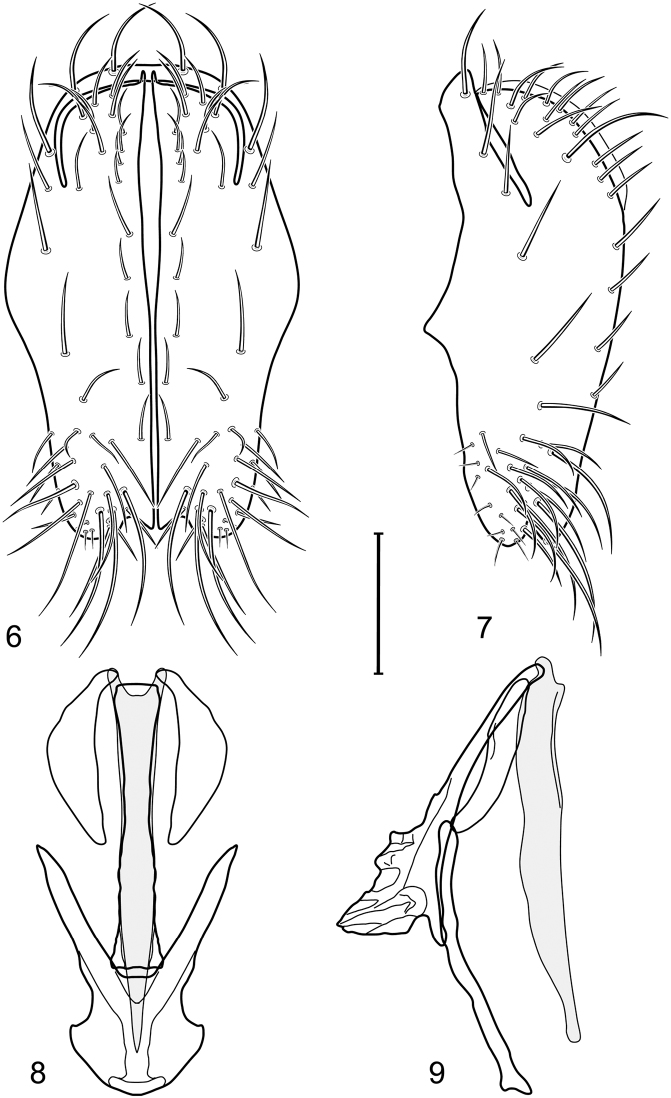
Illustration of *Facitrichophora carvalhorum* sp. n. (male) (Brazil. São Paulo: Praia Puruba) **6** epandrium and cerci, posterior view **7** same, lateral view **8** internal structures of male terminalia (aedeagus [shaded], phallapodeme, gonite, hypandrium), ventral view **9** same, lateral view. Scale bar = 0.1 mm.

*Abdomen*: Tergites dull to partially subshiny, mostly dark brown to grayish brown laterally; tergites 2–3 about equal, shorter than tergites 4–5; tergite 5 of male in dorsal view pointed to narrowly truncate, not bearing row of 6–10, distinctly larger setae along posterior margin with posterodorsal orientation; sternite 4 of male rectangular, sternite 5 V-shaped, neither 4 nor 5 with dense row of setulae along posterior margin. Male terminalia (Figs 6–9): Epandrium connected dorsally above cerci (Figs 6–7), in posterior view rectangularly oval, bulged laterally at midheight, more setulose toward dorsal and ventral ends, dorsal margin bluntly rounded, ventral margin incised, each side broadly and shallowly bifurcate, lateral lobe broadly rounded, in lateral view with ventral apex bifurcate and bearing dense patch of setulae; cerci moderately large, conspicuous; gonite in lateral view rod-like, shallowly sinuous (Fig. 9), in ventral view (Fig. 8) semihemispherical; aedeagus in lateral view simple, tubular, irregularly tapered to narrow, digitiform apex, in ventral view (Fig. 8) elongate, narrow, gradually tapered to acute, ventral apex; phallapodeme with portion toward aedeagal base rod-like, narrow, elongate, parallel sided, opposite end bearing irregularly shaped, pointed keel, in ventral view (Fig. 8) an elongate rod, expanded at each end, end toward aedeagal base incised; hypandrium in lateral view (Fig. 9) elongate, narrow, shallowly curved, in ventral view (Fig. 8) more or less V-shaped, base broad with rounded anterior margin, each posterior arm oriented posterolaterally, elongate, parallel-sided, pointed apically.

#### Type material.

The holotype male is labeled “**BRAZIL.** São Paulo: Praia Puruba (23°21'S, 44°55.6'W; beach), 29Mar2010[,] D. & W. N. Mathis/USNM ENT 00285970 [plastic bar code label]/HOLOTYPE ♂ *Facitrichophora carvalhorum* Mathis & Zatwarnicki, DZUP [red].” The holotype is double mounted (minuten pin in a block of plastic), is in excellent condition, and is deposited in DZUP. Thirty-three paratypes (15♂, 18♀) bear the same locality label data as the holotype with dates from 29–30 Mar 2010.

#### Type locality.

Brazil. São Paulo: Praia Puruba (23°21'S, 44°55.6'W; beach).

#### Other specimens examined.

ARGENTINA. **Buenas Aires:** Rio Santiago, Palo Blanco Berisso (34°51.6'S, 57°52.9'W), 23 Nov 1979, C. M. and O. S. Flint, Jr. (6♂, 1♀; USNM).

COSTA RICA. **Puntarenas:** R. V. S. Golfito, Golfito, Llano Bonito (8°38.4'N, 83°11'W; mangal), 28 Apr 2004, W. Porras (2♀; INBIO).

#### Distribution

**Figure 10.**
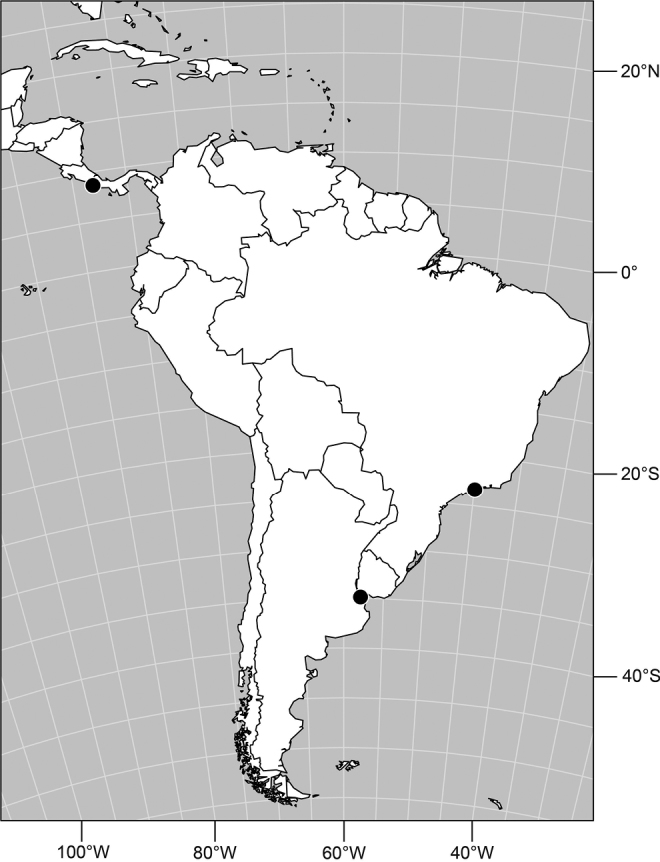
Distribution of *Facitrichophora carvalhorum* sp. n.

(Fig. 10). Neotropical: Argentina (Buenas Aires), Brazil (São Paulo), Costa Rica (Puntarenas).

#### Etymology.

The species epithet, *carvalhorum*, is a plural genitive Latin patronym to honor and recognize the contribution of the A. Bernardo Carvalho family (Bernardo, Monica, Elisa) to this study of the shore flies of Brazil.

#### Remarks.

This species is distinguished externally from congeners most notably by having two posterior dorsocentral setae (in other species there is a single seta), and the anterior dorsocentral seta is slightly smaller than the posterior seta.

### Facitrichophora manza sp. n.

urn:lsid:zoobank.org:act:A85B0B69-12D0-4D0C-AF17-11447AB1E979

http://species-id.net/wiki/Facitrichophora_manza

Figs 11 [Fig F6] 18

#### Diagnosis.

This species is distinguished from congeners by the following combination of characters: Small to moderately small shore flies, body length 1.90–2.55 mm.

**Figures 11–13.**
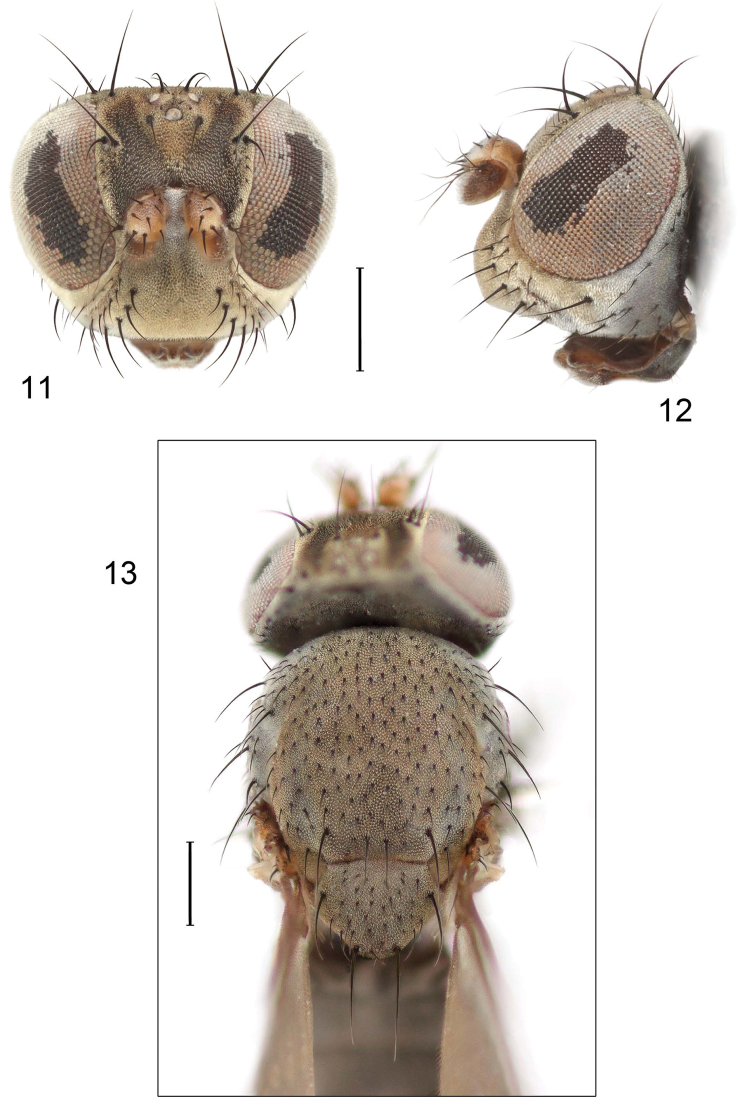
Illustration of *Facitrichophora manza* sp. n. (male) (Trinidad and Tobago. Trinidad. St. Andrew: Lower Manzanilla (12 km S; 10°24.5'N, 61°01.5'W)) **11** head, anterior view **12** same, lateral view **13** thorax, dorsal view.

*Head* (Figs 11–12): Frons brown to black, mesofrons brown, parafrons blackish. Antenna mostly yellow but with basal flagellomere blackish on lateral surface and somewhat dorsally; arista bearing 5–6 dorsal rays. Face broadly and somewhat protruding, lacking bare strip adjacent to carina, bearing 2 rows of setulae, medial row with longer setulae, 5–6, lateral row of setulae shorter, immediately adjacent to parafacial; parafacial becoming distinctly wider posteroventrally, making gena high (about 0.25 eye height), bearing setulae; facial and parafacial microtomentum silvery white to golden yellowish white, sometimes not concolorous; gena relatively short, less than 1/4 eye height; eye-to-cheek ratio 0.21–0.26.

*Thorax* (Fig. 13): Mesonotum subshiny, generally bronzish, becoming grayish anterolaterally, with sparse, whitish to grayish microtomentum; a single pair of posterior dorsocentral setae; prescutellar acrostichal setae small but evident, inserted close together; scutellum trapezoidal in dorsal view with posterior margin truncate, lateral margins essentially straight, bearing 2 primary scutellar along lateral margins but otherwise lacking larger setae between primary setae. Wing hyaline or uniformly faintly infuscate; costal vein ratio 0.56–0.64; M vein 0.58–0.60. Coxae and femora grayish to blackish gray; tibiae and tarsi yellowish.

**Figures 14–17. F6:**
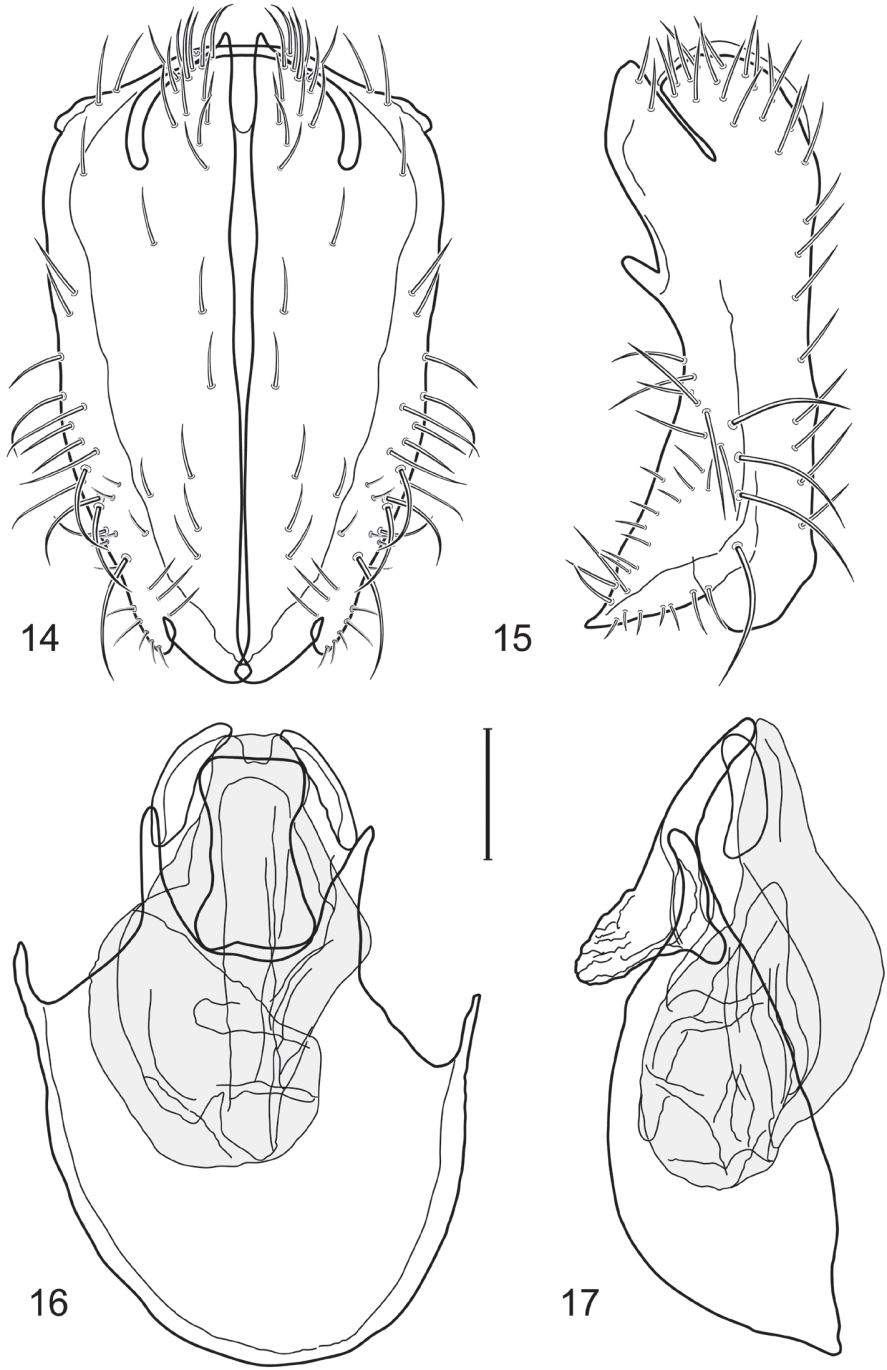
Illustration of *Facitrichophora manza* sp. n. (male) (Mexico. Veracruz-Llave: Isla del Amor) **14** epandrium and cerci, posterior view **15** same, lateral view **16** internal structures of male terminalia (aedeagus [shaded], phallapodeme, gonite, hypandrium), ventral view **17** same, lateral view. Scale bar = 0.1 mm.

*Abdomen*: Tergites 2-3 subequal in length; tergites 3 of male with a broadly produced posteroventral process on each side (on venter); tergite 4 of male with a short point at anteroventral and posteroventral corner; tergite 5 of male and a short point toward anteroventral ; corner; tergites 4 and especially 5 of male elongate; tergite 5 of male truncate posteriorly, bearing short and decumbent setae at posterior margin; sternite 4 of male rectangular, sternite 5 V-shaped, neither 4 nor 5 with dense row of setulae along posterior margin. Male terminalia (Figs 14-17): Generally bluntly wedge shaped in posterior view; epandrium narrowly connected dorsally, in posterior view (Fig. 14) lateral margins gradually tapered, more so ventrally, ventral margin bilobed with short, small ventrolateral lobe, setulae more evident laterally, in lateral view (Fig. 15) generally straight, especially posterior margin, bilobes conspicuous, anteroventral lobe oriented anteriorly and acutely pointed, posterior lobe irregularly rounded; cerci comparatively small, broadly fused with epandrium; aedeagus in lateral view (Fig. 17) narrow basally, thereafter ventrally becoming swollen, complex, in ventral view (Fig. 16) asymmetrical, longer than wide; phallapodeme in lateral view (Fig. 17) with conspicuous, extended, wedge-shaped keel, extension toward aedeagal base elongate, narrow, opposite end short, stubby, in ventral view (Fig. 16) hour glass shaped, relatively short, both ends slightly expanded; gonite in lateral view (Fig. 17) relatively short, shallowly curved, ends rounded, in ventral view (Fig. 16) shallowly curved, length less than lengthy of phallapodeme; hypandrium in lateral view (Fig. 17) relatively large, broadly and shallowly pocket-like, posterior extension narrower and longer than anterior margin, in ventral view (Fig. 16) slightly asymmetrical, anterior margin broadly rounded, posterior margin as 2 paired, narrow prongs, medial pair forming a deep emargination.

#### Type material.

The holotype male is labeled “**TRINIDAD.** St. Andr[ew].: Low[er] Manzanilla(12km S; 10°24'N, 61°02'W)[,] bridgeNariva Riv[er], 20–27Jun1993, WNMathis/USNM ENT 00285971 [plastic bar code label]/HOLOTYPE ♂ *Facitrichophora manza* Mathis & Zatwarnicki, USNM [red].” The holotype is double mounted (minuten pin in a block of plastic), is in excellent condition, and is deposited in the USNM. Twenty paratypes (9♂, 11♀; USNM) bear the same label data as the holotype.

#### Type locality.

Trinidad and Tobago. Trinidad. St. Andrew: Lower Manzanilla (12 km S; 10°24.5'N, 61°01.5'W), bridge over Nariva River.

#### Other specimens examined.

COSTA RICA. **Guanacaste:** Cañas, Rio Tempisque (10°25.6'N, 85°05.7'W), 7–9 Jul 2003, D. Briceño (4♂, 5♀; INBIO); Isla Pájaros (9°52.1'N, 84°54.6'W), 16 Nov 2004, B. Gamboa (2♂; INBIO); Estero Caletas (9°46.5'N, 85°15.6'W), 22 Oct 2003, W. Porras (1♀; INBIO); Nandayure, Playa Bejuco (10°56'N, 85°51.7'W), 21 Oct 2003, D. Briceño (6♂, 4♀; INBIO); Nosara, Privado Nosara (9°59'N, 85°39'W), 15 Jun 2004, D. Briceño (1♀; INBIO); Palo Verde, Nicoya, Isla Saíno (10°19'N, 85°21'W), 16 Nov 2004, B. Gamboa (1♀; INBIO). **Limón:** Talamanca, Estación Gandoca (9°37.4'N, 82°41.7'W), 18 May 2004, W. Porras (1♀; INBIO). **Puntarenas:** San Pedrillo (8°37.2'N, 83°44.1'W), 12–15 Aug 2001, D. and W. N. Mathis (4♂, 5♀; USNM).

CUBA. **Cienfuegos:** Soledad, Jardin Botánico (22°7.5'N, 80°19.2'W), 16-18 Jul 1947, W. L. Nutting (1♀; AMNH).

MEXICO. **Veracruz-Llave:** Isla del Amor (19°05.6'N, 96°06'W), 6 Sep 1975, A. G. Soika (2♂, 1♀; USNM).

#### Distribution

**Figure 18.**
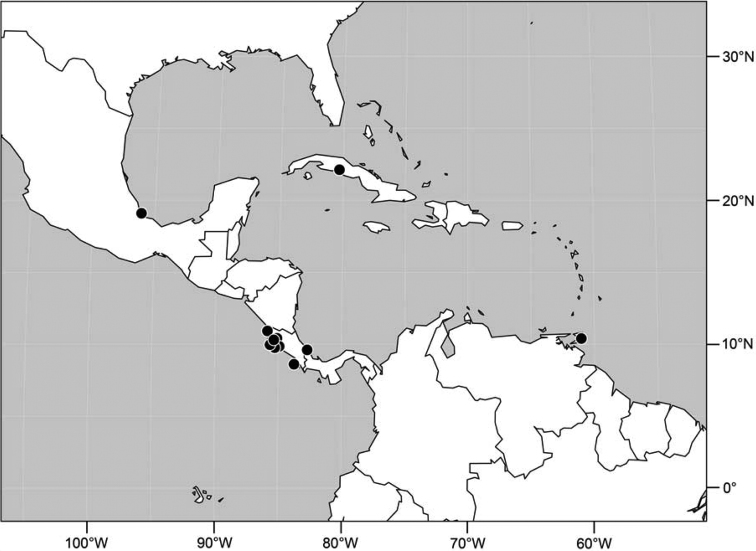
Distribution of *Facitrichophora manza* sp. n.

(Fig. 18). Neotropical: Costa Rica (Guanacaste, Limón, Puntarenas), Mexico (Veracruz-Llave), Trinidad and Tobago, West Indies (Cuba).

#### Etymology.

The species epithet, *manza*, is an abbreviated reference to the general area, Lower Manzanilla, where this species was first discovered on Trinidad.

#### Remarks.

Discovery of this species along the coast of Trinidad was our first indication of this genus. Some specimens were initially classified as an undescribed species of *Polytrichophora* and others within the genus *Discocerina*. Additional species were discovered and with careful study of them, including characters of the male terminalia, we became aware of a monophyletic group that is somewhat between these two genera.

Although similar to *Facitrichophora panama*, this species is distinguished by having a grayish brown to bronzish brown mesonotum, by having two lateral, scutellar setae, and in males, by having an elongated tergite four (conspicuously longer than tergite three and subequal to tergite five). In addition, the ventrolateral margin of tergite three is produced into a curved, narrow, parallel-sided process, and the anteroventral angle of tergite five is slightly projected and acutely pointed.

### Facitrichophora panama sp. n.

urn:lsid:zoobank.org:act:F5D7C973-9859-49CA-9F91-1693E5A6398A

http://species-id.net/wiki/Facitrichophora_panama

Figs 19 23

#### Diagnosis.

This species is distinguished from congeners by the following combination of characters: Small to medium-sized small shore flies, body length 1.84-3.20 mm.

*Head*: Frons gray to brown, mesofrons gray, parafrons grayish brown. Antenna mostly grayish brown to yellow; basal flagellomere blackish on lateral surface and somewhat dorsally; arista bearing 5–6 dorsal rays. Face silvery to whitish gray, broadly and somewhat protruding, lacking bare strip adjacent to carina, bearing 2 rows of setulae, medial row with longer setulae, 5–6, lateral row of setulae shorter, immediately adjacent to parafacial; parafacial becoming distinctly wider posteroventrally, making gena high (about 0.25 eye height), bearing setulae; facial and parafacial microtomentum silvery white to golden yellowish white, sometimes not concolorous; gena relatively high, equal or greater than 1/4 eye height; eye-to-cheek ratio 0.25–0.44.

*Thorax*: Mesonotum subshiny, especially medially, generally grayish peripherally to shiny copperish to blue, with sparse, whitish to grayish microtomentum; a single pair of posterior dorsocentral setae; prescutellar acrostichal setae small but evident, inserted close together; scutellum obtuse triangular, posterior margin shallowly rounded in dorsal view, lateral margins essentially straight, bearing 2 primary scutellar along lateral margins but otherwise lacking larger setae between primary setae. Wing hyaline or uniformly faintly infuscate; costal vein ratio 0.54–0.75; M vein 0.58–0.68. Coxae, femora, and tibiae grayish to blackish gray; hindtibia yellowish at apices; tarsi yellowish.

**Figures 19–22.**
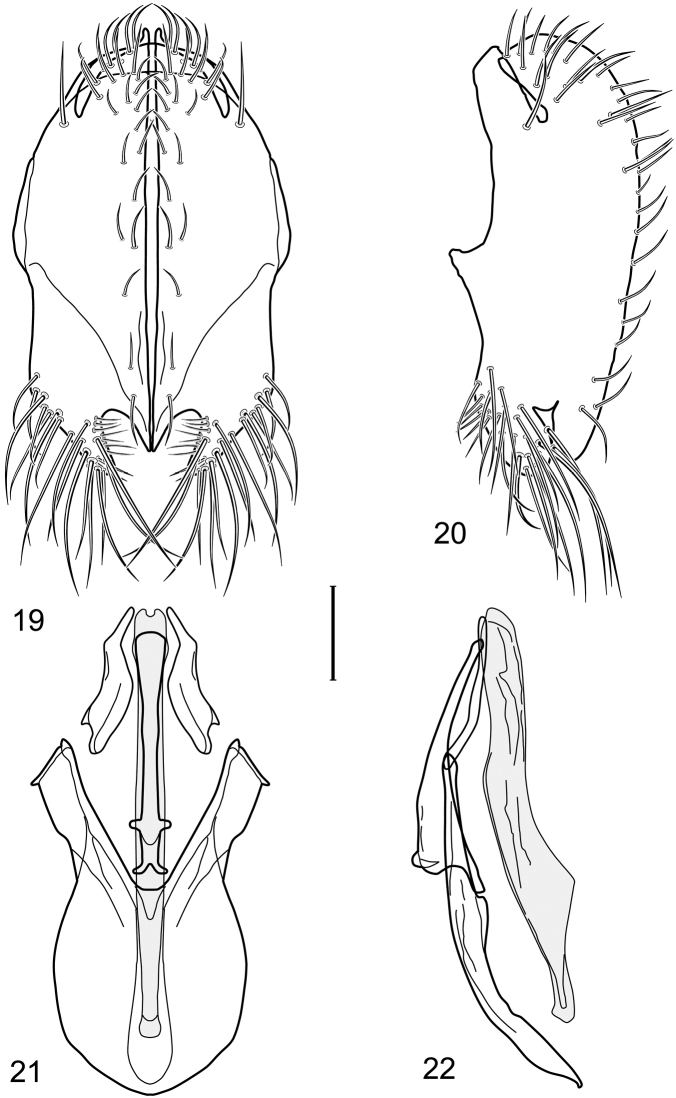
Illustration of *Facitrichophora panama* sp. n. (male) (Panama. Darien: Garachine) **19** epandrium and cerci, posterior view **20** same, lateral view **21** internal structures of male terminalia (aedeagus [shaded], phallapodeme, gonite, hypandrium), ventral view **22** same, lateral view. Scale bar = 0.1 mm.

*Abdomen*: Tergite 5 of male narrowed apically. Male terminalia (Figs 19–22): Epandrium connected dorsally above cerci (Figs 19–20), in posterior view polygonal oval, slightly wider at midheight, dorsal margin broadly rounded, ventral margin deeply incised, each side broadly and deeply bifurcate, lateral lobe narrowly triangular, apex narrowly rounded, more setulose toward dorsal and ventral ends, in lateral view with ventral apex bifurcate and bearing dense patch of setulae; cerci moderately large, conspicuous; gonite in lateral view rod-like, shallowly sinuous (Fig. 22), in ventral view (Fig. 21) semihemispherical; aedeagus in lateral view simple, elongate, tubular, irregularly tapered to narrow, digitiform process, apex truncate, in ventral view (Fig. 21) elongate, narrow, gradually tapered to ventral apex; phallapodeme with portion toward aedeagal base rod-like, very narrow and elongate, tapered with apical half parallel sided, hypandrial end bearing irregularly shaped, pointed keel, in ventral view (Fig. 21) an elongate rod, expanded at each end, end toward aedeagal base shallowly incised; gonite in lateral view (Fig. 22) elongate, very narrow, shallowly and angularly curved, in ventral view (Fig. 21) broadly zigzagged, narrower toward aedeagal base, becoming generally broader toward hypandrium; hypandrium in lateral view (Fig. 22) elongate, very narrow, shallowly curved, in ventral view (Fig. 21) more or less deeply V-shaped, base broad with rounded anterior margin, each posterior arm oriented posterolaterally, elongate, parallel-sided, pointed apically.

#### Type material.

The holotype male is labeled “PANAMA[.] DarienProv[incia][,] Garachine/Feb 1953[,] FSBlanton/USNM ENT 00285973 [plastic bar code label]/HOLOTYPE ♂ *Facitrichophora panama* Mathis & Zatwarnicki, USNM [red].” The holotype is double mounted (glued to a paper triangle), is in moderate condition (arista missing; some head setae missing or misoriented), and is deposited in the USNM. One male paratype (dissected; USNM) bears the same label data as the holotype. Other paratypes are as follows: PANAMA. **Canal Zone:** San Lorenzo (9°07.9'N, 79°32.1'W), 15 Aug 1952, F. S. Blanton (1♀; USNM). **Darien:** El Real (8°07.8'N, 77°43.6'W), 8 Aug 1952, F. S. Blanton (1♀; USNM). **Los Santos:** Bayano (7°57'N, 80°20'W), 8 Apr 1952, F. S. Blanton (1♀; USNM).

#### Type locality.

Panama. Darien: Garachine (8°04'N, 78°22'W).

#### Distribution

**Figure 23.**
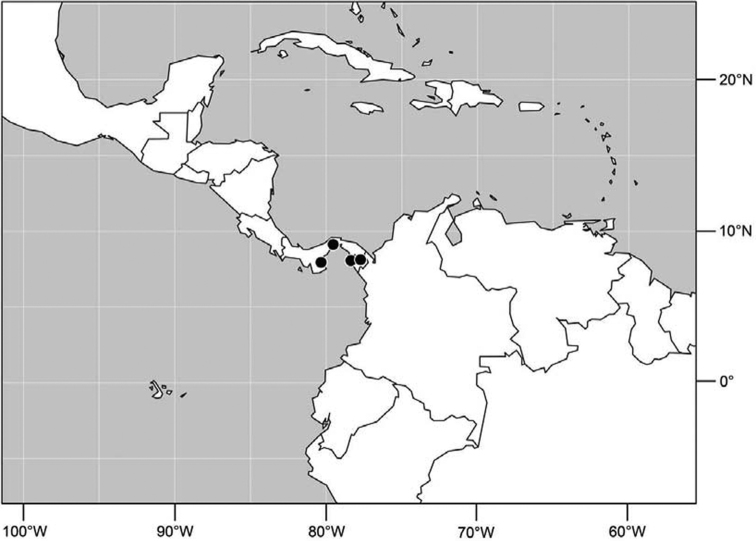
Distribution of *Facitrichophora panama* sp. n.

(Fig. 23). Neotropical: Panama (Canal Zone, Darien, Los Santos).

#### Etymology.

The species epithet, *panama*, refers to the country where this species was collected and is a noun in apposition.

#### Remarks.

Although similar to *Facitrichophora manza* (similarities include having a single dorsocentral seta, the mesonotum grayish brown to bronzish brown, and having two lateral scutellar setae), it is distinguished by having male tergites three and four subequal in size (tergite four not unusually longer as in *Facitrichophora manza*); male tergite three is not produced ventrolaterally; and male tergite five is rounded or angulate (not projected anteroventrally and acutely pointed as in *Facitrichophora manza*).

### Genus Polytrichophora

Cresson

http://species-id.net/wiki/Polytrichophora

Polytrichophora Cresson 1924: 161. Type species: *Polytrichophora agens*Cresson 1924: 161, original designation. Cresson 1942: 114 [review of Nearctic species]; 1946: 143, 151 [review of Neotropical species, key]. Wirth 1965: 739-740 [Nearctic catalog]; 1968: 8-9 [Neotropical catalog]. Zatwarnicki and Mathis 2001: 50 [generic diagnosis].

#### Diagnosis.

**Figures 24–27.**
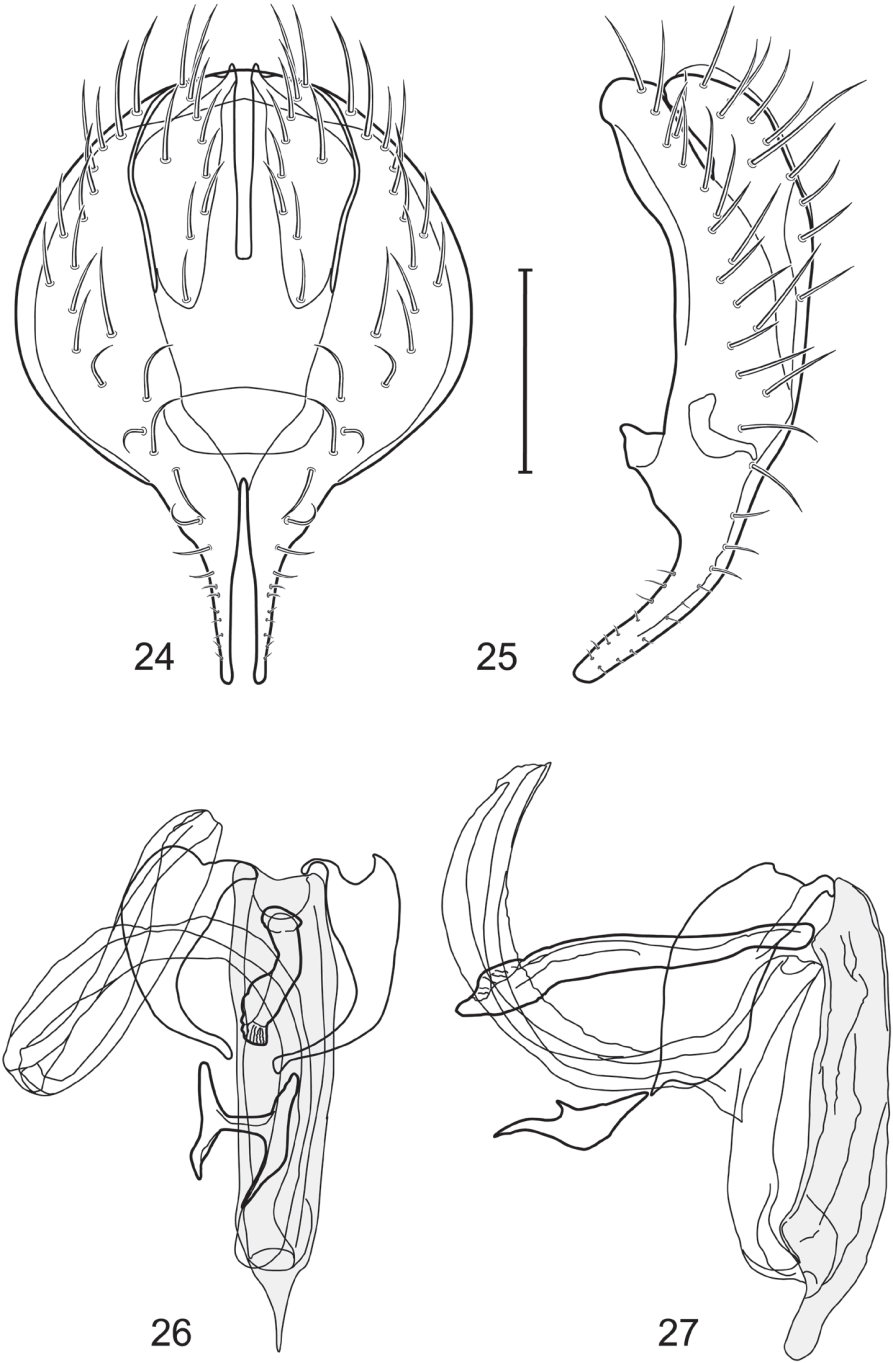
Illustration of *Polytrichophora adarca* sp. n. (male) (Mexico. Chiapas: Boca de Cielo) **24** epandrium and cerci, posterior view **25** same, lateral view **26** internal structures of male terminalia (aedeagus [shaded], phallapodeme, gonite, hypandrium), ventral view **27** same, lateral view. Scale bar = 0.1 mm.

Small to moderately small shore flies, length 1.4–3.5 mm; generally densely microtomentose, dull species (Figs 29–31). *Head* (Figs 29–30): Frons lacking anterior, proclinate, fronto-orbital seta; face substantially prominent at level of dorsal facial seta; antennal grooves generally sharply defined ventrally; facial setae usually comprising 8 setae, these not arising from shiny papilla and generally decreasing in size from dorsum to venter, appearing as 2 series (Figs 29–30) due to divergent orientation of setae in series; setae of primary series inclinate (setae 1, 2, 5, and 7), generally larger than setae of secondary series except for seta 2, which is much reduced and inserted laterad and sometimes slightly ventrad of seta 1, seta 1 largest of all facials, inclinate (cruciate with opposite seta), but not arising from shiny papilla; setae of secondary series oriented dorsolaterally to laterally (setae 3, 4, 6, and 8), usually smaller series than primary series; face lacking a distinctly dorsoclinate seta at ventral extremity; parafacial narrow to moderately wide, generally bearing setulae although sometimes fine and pale or lacking; gena variable but generally short to moderately high. Eye generally oval, conspicuously microsetulose, bearing numerous interfacetal setulae; maxillary palpus yellow. *Thorax* (Fig. 31): Single presutural and postsutural supra-alar setae well developed; postsutural supra-alar seta reduced, about 1/2 length of postalar seta; acrostichal setae present; notopleuron bearing several setulae in addition to 2 larger setae; anterior notopleural seta inserted conspicuously closer to posterior notopleural seta than to postpronotal seta. Wings transparent; costa bearing 3–5 long, dorsal setae between humeral and subcostal breaks. Forefemur normally developed, lacking row of short, stout setae along posteroventral surface; hindtibia lacking a preapical, ventral, spur-like seta. *Abdomen*: Tergites variable, but mostly unicolorous, if bicolorous, bearing pale colored areas laterally, but these not distinctly wedge-shaped; 4th tergite of ♂ only slightly longer than 3rd. Male terminalia (Figs 24–27): Epandrium complete or separate dorsally, in posterior view more or less elliptical, 2× longer than wide, in ventral half gradually tapered, or pyriform, broader basally and tapered irregularly to apex, on whole surface bearing distinct setae, in lateral view apical half slightly widen by extension of its ventral portion, apical section of ventral margin often with setae; cerci fused ventrolaterally to medial margin of epandrium, in posterior view generally slightly elongate or hemispherical; gonites rod-shaped without setae, rarely lobate; aedeagus strongly elongate, about 4–6× longer than wide, in dorsal view base generally narrowly triangular, apex rounded, in lateral view trunk of aedeagus with uniform thickness, apex pointed and generally bearing subapically a narrow, membranous flap that generally folds back under aedeagus with about 2/3 length of aedeagus to battered tape 2× as long as aedeagus; hypandrium in dorsal view H-shaped, with transverse cross-bar, anterior arms arcuate, approximate to each other, with narrow arms with uniform thickness or unequal lobate broadened, sometimes to different degree at apex interrupted or/and with reduced cross-bar, in lateral view hypandrium flat, slightly directed dorsally; phallapodeme separate from aedeagus, in posterior view elongate with extension at base and bifurcate anteriorly, in lateral view subtriangular or subrectangular, with dorsal margin slightly bent ventrally, ventral margin irregularly U-shaped, middle part of apodeme with ventral broad appendix, sometimes slightly directed anteriad; ejaculatory apodeme absent.

#### Distribution.

This is one of the few genera of Ephydridae that occurs worldwide and in temperate or tropical environments. The New World, with 23 species, currently has greater species diversity. Worldwide there are 35 species.

#### Natural history.

Specimens of *Polytrichophora* are usually associated with mud-shore and sand-shore habitats (Deonier 1965) or rarely with a marsh-reed habitat (Scheiring and Foote 1973). We usually found greatest diversity and abundance while collecting on mud and sandy shores that had a mat-like covering of algae. Some species, as will be noted with their individual treatment, are tolerant of mildly saline conditions and occur on mud and sandy shores where the water is brackish.

#### Discussion.

*Polytrichophora*, along with *Discocerina*, *Facitrithophora*, and *Hydrochasma* Hendel (New World genera), form a monophyletic lineage within the Discocerinini, as noted previously under the tribal diagnosis. *Polytrichophora* may be distinguished from other genera of the *Discocerina* group by the following synapomorphies: (1) facial setae with a secondary series of dorsolaterally inclined setae laterad to inclinate primary series (Figs 29–30); (2) parafacial setulose (Figs 29–30); (3) gena short to moderately high (Fig. 30); (4) eye densely pilose; (5) cerci usually fused ventrally or ventrolaterally with epandrium; (6) epandrium with dorsal portion above the cerci weakened, thin, or absent; (7) anterior margin of hypandrium deeply incised; (8) subapical ventral aedeagal appendix (the plesiomorphic condition is an aedeagus without appendices).

#### Key to species of *Polytrichophora* from the New World

**Table:** 

1	Parafacial color contrasted sharply with much darker midfacies; parafacial with little or no dilation ventrally; abdomen mostly shiny, brownish black to black	2
–	Parafacials not markedly differing from midfacies in color; parafacials with slight to considerable ventral dilation; abdomen dull to subshiny, gray to blackish brown	3
2	Mesonotum mostly dull, grayish brown to brown. Midfacies with vitta extended from oral margin to antennal bases, gray to yellowish gray, sharply contrasted with darker, lateral margins of midfacies	*Polytrichophora pulchra* (Cresson)
–	Mesonotum subshiny to shiny, bronzish black. Midfacies either lacking a distinctly colored vitta or if vitta present then not extended dorsally past midheight	*Polytrichophora desmata* (Williston)
3	Forefemur with comb-like row of 9–10, short, stout setae along apical half of anteroventral surface	4
–	Forefemur lacking row of setae as described above	6
4	Anterior margin of wing with 10–12 larger, spine-like setae interspersed with smaller setae; forefemur with row of 6–7 long setae along posteroventral surface greater in length than width of femur at same level	*Polytrichophora adarca* sp. n.
–	Anterior margin of wing lacking larger, spine-like setae; forefemur with posteroventral row of setae shorter	5
5	Epandrium in posterior view (Fig. 48) with lateral margins nearly straight, not rounded; ventral epandrial processes in lateral view (Fig. 49) slightly expanded (Nearctic)	*Polytrichophora conciliata* Cresson
–	Epandrium in posterior view (Fig. 59) with lateral margins arched, rounded; ventral epandrial processes in lateral view (Fig. 60) only slightly expanded (Brazil)	*Polytrichophora marinoniorum* sp. n.
6	Parafacials becoming 3–4 times wider ventrally; gena high, at least 1/4 eye height	7
–	Parafacials 2–3 times wider ventrally; gena less high, less than 1/4 eye height	8
7	Abdomen mostly pale gray dorsally, becoming slightly tinged with bluish gray medially and pale tan basally; forefemur with row of setae along posteroventral surface less well developed, none equal to width of femur	*Polytrichophora arnaudorum* sp. n.
–	Abdomen mostly dark brown to black dorsally, subshiny; forefemur with row of 7–9, well-developed, evenly spaced setae along posteroventral surface each equal to width of femur	*Polytrichophora agens* Cresson
8	Tergite 5 of male with row of 6–10, distinctly larger setae along extreme posterior margin with posterodorsal orientation	9
–	Tergite 5 of male lacking row of setae as above	10
9	Small species, length 2.40 mm or less; abdominal tergites 1–4 bicolored, mostly blackish brown dorsally, gray lateroventrally, tergite 5 of male also gray on posterior 1/3; sternites 3–4 of male with dense patch of setulae along posterior margin	*Polytrichophora reginae* Mathis
–	Large species, length 2.80 mm or longer; abdomen, including tergite 5 of male, entirely black dorsally, mostly shiny, slightly grayish ventrally; sternites 4–5 of male setulose but not with dense patches	*Polytrichophora prolata* sp. n.
10	Row of stout setae along posteroventral surface of forefemur short, length less than width of foretibia; abdominal tergite 5 of male bluntly rounded to truncate	11
–	Row of stout setae along posteroventral surface of forefemur longer, subequal or longer than width of foretibia; abdominal tergite 5 of male pointed apically	16
11	Dorsum of abdominal tergite 5 of male entirely black, contrasted with paler-colored preceding tergites; foretibia mostly gray	*Polytrichophora salix* sp. n.
–	Dorsum of abdominal tergite 5 of male not darker than preceding tergites and usually with distinct, semicircular, grayish areas laterally; foretibia mostly yellowish	12
12	Legs, including most of coxae, yellow; epandrium rounded ventrally and with a subapical, anterior process (Figs 53–55)	*Polytrichophora flavella* sp. n.
–	Femora and some of tibiae grayish, microtomentose; epandrium lacking a secondary process	13
13	Ventral extensions of epandrium elongate, narrow, and in lateral view sinuous (Figs 74–75)	*Polytrichophora sinuosa* sp. n.
–	Ventral extensions of epandrium not elongate and sinuous in lateral view	14
14	Ventral edge of epandrium in lateral view (Fig. 44) bluntly rounded and mostly straight, not curved anteriorly; posterior margin of epandrium about 1/4 from ventral margin with a small prominence that bears a patch of setulae on, ventral margin broadly rounded; 7th tergite of ♀ with 3–4 conspicuously larger setae inserted laterally along posterior margin* P. barba* sp. n.	1
–	Ventral edge of epandrium in lateral view distinctly pointed and curved anteriorly; posterior margin of epandrium lacking small prominence at basal 1/4 that bears a short tuft of setulae	15
15	Epandrium in lateral view (Fig. 70) with secondary prong; anterior curvature of ventral portion gradual	*Polytrichophora setulosa* (Cresson)
–	Epandrium in lateral view (Fig. 65) with a single, apical, pointed process; ventral curvature more abrupt, nearly at right angle	*Polytrichophora rostra* sp. n.
16	Area between anterior arms of hypandrium membranous	17
–	Area between anterior arms partially sclerotized	18
17	Epandrium with ventral margin gradually tapered to apex (Figs 104–105); hypandrium large, truncate anteriorly, and more heavily sclerotized (Fig. 106)	*Polytrichophora setigera* (Cresson)
–	Epandrium with ventral margin abruptly tapered to apex (Figs 94-95); hypandrium small, pointed anteriorly, and weakly sclerotized (Fig. 96)	*Polytrichophora orbitalis* (Loew)
18	Epandrium with posteroventral edge in lateral view very slightly recurved; taper at apex of epandrium abrupt (Figs 89-90)	*Polytrichophora mimbres* sp. n.
–	Epandrium with posteroventral edge evenly curved; taper at apex more gradual and acute (Figs 109–110)	*Polytrichophora sturtevantorum* sp. n.

##### The *conciliata* group

#### Species included: 

*Polytrichophora adarca* sp. n., *Polytrichophora agens* Cresson, *Polytrichophora arnaudorum* sp. n., *Polytrichophora barba* sp. n., *Polytrichophora conciliata* Cresson, *Polytrichophora flavella* sp. n., *Polytrichophora marinoniorum* sp. n., *Polytrichophora rostra* sp. n., *Polytrichophora setulosa* Cresson, *Polytrichophora sinuosa* sp. n.

**Discussion.**Synapomorphies for the *conciliata* group are: phallapodeme bar-like and separated from hypandrium; hypandrium small, H- or C-shaped. Within the *conciliata* group there are two assemblages of species based primarily on the shape of the epandrium in lateral view. The first assemblage, which includes *Polytrichophora agens* Cresson, *Polytrichophora barba*, *Polytrichophora flavella*, *Polytrichophora rostra*, *Polytrichophora setulosa* Cresson, is characterized by a wide hypandrium that bears a shallow and arched posterior margin with long apical arms (reduced in *Polytrichophora agens*). Suggested relationships among these species in parenthetic notation are: *Polytrichophora barba* (*Polytrichophora rostra*, *Polytrichophora setulosa* Cresson) (*Polytrichophora agens* Cresson, *Polytrichophora flavella*). An apomorphy for *Polytrichophora barba* is the presence of a dense tuft of setulae on the epandrium. Specimens of *Polytrichophora rostra* and *Polytrichophora setulosa* have a crescent-shaped ventral epandrial process (surstylus) and have pronounced asymmetry of the anterior hypandrial arms. Specimens of *Polytrichophora agens* and *Polytrichophora flavella* have an asymmetrical epandrium.

The second assemblage, which includes *Polytrichophora adarca*, *Polytrichophora arnaudorum*, *Polytrichophora conciliata* Cresson, *Polytrichophora marinoniorum*, *Polytrichophora sinuosa*, is characterized by a small H-shaped hypandrium in ventral view, and the relationships of its included species in parenthetic notation are: *Polytrichophora arnaudorum* (*Polytrichophora adarca* ((*Polytrichophora conciliata* Cresson, *Polytrichophora marinoniorum*) *Polytrichophora sinuosa*))). An apomorphy for *Polytrichophora arnaudorum* is the long anterior process of the aedeagus (basiphallus). For other species of this group, there is a narrow ventral epandrial process (surstylus). Specimens of *Polytrichophora adarca* have a shortened ventral epandrial process (surstylus). For other species, there is a lateral projection on the distiphallus and a narrowed anterior process of the aedeagus (basiphallus). Specimens of *Polytrichophora conciliata* Cresson and *Polytrichophora marinoniorum* have a digitiform ventral epandrial process (surstylus), and *Polytrichophora sinuosa* has an elongated ventral epandrial process (surstylus).

### Polytrichophora adarca sp. n.

urn:lsid:zoobank.org:act:5D23AFB8-0616-4200-9AD0-3AA475CCFA6C

http://species-id.net/wiki/Polytrichophora_adarca

Figs 24 28

Polytrichophora conciliata in part of authors, not Cresson [misidentification]. Mathis and Zatwarnicki 1995: 184 [world catalog].

#### Diagnosis.

This species is distinguished from congeners by the following combination of characters: Small to moderately small shore flies, body length 1.45–2.10 mm.

*Head*: Frons dull, heavily microtomentose, 2-toned, mostly tan with some faint golden reflections on posterior 2/3, anterior 1/3 (from level of fronto-orbital setae anteriad), gray. Antenna mostly yellow to yellowish orange, anterior portion of pedicel and basodorsal area of basal flagellomere with some blackish coloration; arista with 5 dorsal rays. Face at narrowest point about equal to combined length of pedicel and basal flagellomere; face densely microtomentose, microtomentum with shiny to pearly luster, mostly white but with considerable gold coloration in antennal grooves and extended laterally onto parafacial; parafacial not markedly differing from midfacies in color, more golden dorsally, becoming whiter ventrally; parafacial with slight to considerable ventral dilation, 2–3 times wider ventrally than dorsally; gena relatively short, subequal to height of basal flagellomere, gena-to-eye ratio 0.12–0.14.

*Thorax*: Mesonotum mostly dull, densely microtomentose, concolorous with posterior 2/3 of frons; pleural area blackish gray. Stout setae on apical half; anterior margin of wing lacking large, spine-like setae; costal vein ratio 0.63–0.75; M vein ratio 0.48–0.53. Forefemur lacking row of 9–10 short, stout setae along apical half of anteroventral surface; forefemur with anteroventral comb-like row of 7–10 short setulae, each equal to width of femur; tibiae mostly yellowish; basal tarsomeres yellow, apical 1–2 brown.

*Abdomen*: Tergites dull to subshiny, gray to blackish brown; tergites 1–4 bicolored, mostly blackish brown dorsally, gray lateroventrally; tergites 3–4 of male subequal, 4th not unusually longer, tergite 3 not produced ventrolaterally; dorsum of tergite 5 of male not darker than preceding tergites and usually with distinct, semicircular, grayish areas laterally; tergite 5 of male in dorsal view blunt, truncate, bearing row of 6–10, distinctly larger setae along extreme posterior margin with posterodorsal orientation; tergite 5 of male also gray on posterior 1/2–1/3; sternites 3–4 of male with dense row of setulae along posterior margin; sternite 5 as 2 subtriangular sternites. Male terminalia (Figs 24–27): Epandrium narrowly connected dorsally above cerci (Fig. 24), in posterior view tapered ventrally to an acute point (Fig. 24); ventral third of epandrium in lateral view curved anteriorly, slightly expanded apically, narrowly spatulate, slightly curved anteroventrally, pointed apically, and with subapical ledge that also ends ventrally in a point, bearing a large, subapical seta laterally (Fig. 27); gonite in lateral view rod-like, shallowly curved (Fig. 27); aedeagus in lateral view (Fig. 27) with basiphallus tubular, very shallowly sinuous, narrowed apically to form digitiform process, distiphallus membranous, longer and narrower than basiphallus, folded back on basiphallus, angulate at basal 1/3, thereafter distinctly curved; phallapodeme in lateral view (Fig. 27) elongate, narrow, bar-like, narrower basally, gradually expanded toward apex, keel much reduced; hypandrium in lateral view (Fig. 27) with apices acutely tapered, swollen medially, in posterior view (Fig. 26) deeply H-shaped, anterior arms with one side longer.

#### Type material.

The holotype male is labeled “BARBADOS. Chr[i]st Ch[u]rch: Graeme Hall Swamp[,] 13°04.2'N, 59°34.7'W[,] 21–22May1996, D. & W. Mathis, H. Williams/ HOLOTYPE ♂ Polytrichophora adarca Mathis & Zatwarnicki, USNM [red]/USNM ENT 00141761 [plastic bar code label].” The holotype is double mounted (minuten pin in a block of plastic), is in excellent condition, and is deposited in the USNM. Thirty-six paratypes (32♂, 4♀; USNM) bear the same locality data as the holotype with dates from 21 May–12 Sep 1996, 1997. Other paratypes are as follows: BARBADOS. St. Andrew: Belleplaine (13°14.8'N, 59°33.6'W), 21 May 1996, D. and W. N. Mathis, H. B. Williams (4♂, 1♀; USNM); Long Pond (13°15.1'N, 59°33.3'W), 21 May-10–11 Sep 1996, D. and W. N. Mathis, H. B. Williams (12♂, 8♀; USNM); Walkers Bridge (13°15'N, 59°34.5'W), 11 Sep 1996, W. N. Mathis (1♂; USNM).

Type locality. Barbados. Christ Church: Graeme Hall Nature Sanctuary (13°04.2'N, 59°34.7'W; swamp).

Other specimens examined. Neotropical. BELIZE. Stann Creek: Cockscomb Basin Wildlife Sanctuary (16°47'N, 88°30'W), 5–6 Apr 1993, W. N. Mathis (♂, ♀; USNM); Glover'S Reef (Southwest Cay; 16°42.9'N, 87°51'W), Jul 1989, W. N. Mathis (7♂, 3♀); Man of War Cay (17°13'N, 87°54'W), Jun-Nov 1987, 1989, 1990, D. and W. N. Mathis, H. B. Williams (19♂, 24♀; USNM); Mullins River (17 km N Dangriga; 17°06.2'N, 88°17.8'W), 29 Mar 1988, W. N. Mathis (1♀; USNM); Round Cay (near Coco Plum Cay; 16°52'N, 88°07'W), Mar 1988, W. N. Mathis (4♂, 5♀; USNM); Saddle Cay (17°13.9'N, 87°31'W), Mar 1988, W. N. Mathis (3♂, 4♀; USNM); Sittee River, Possum Point Biological Station (16°52.1'N, 88°22.5'W), 6 Nov 1987, D. and W. N. Mathis (1♀; USNM); Stewart Cay (16°46'N, 88°09'W), Mar-Jul 1988, 1989, W. N. Mathis, H. B. Williams (4♂, 14♀; USNM); Tobacco Range (16°52.9'N, 88°04.9'W), Jul 1989, W. N. Mathis, H. B. Williams (4♂, 4♀; USNM); Twin Cays (Aanderaa Flats, dock area, east shore of East Island, mud flat near Lair Channel, south end of West Island, West Bay; 16°49.9'N, 88°06.1'W), Jan-Jul 1987, 1988, 1989, 1990, W. N. Mathis, C. Feller, H. B. Williams (61♂, 84♀; USNM); Wee Wee Cay (16°45.9'N, 88°08.6'W), Mar–Jul 1987, 1988, 1989, W. N. and D. Mathis, C. Feller (11♂, 25♀; USNM). Belize District: Turneffe Islands: Rope Walk Cay (17°13'N, 87°51'W), Mar 1993, W. N. Mathis (1♀).

COSTA RICA. Guanacaste: Bagaces, Fortuna, Z. P. Miravalles, Sector Cabro Muco (10°42.7'N, 85°09.3'W; 980 m), 8-31 Jul 2002, J. D. Gutiérez (2♀; INBIO); Cañas, Rio Tempisque (10°25.6'N, 85°05.7'W), 7–9 Jul 2003, D. Briceño (2♂, 6♀; INBIO); Nandayure, Isla Berrugate (10°02.6'N, 85°10.6'W; 0–100 m), 27 Aug 2003, W. Porras (1♀; INBIO); Nicoya, Isla Pájaros (09°52.1'N, 84°54.6'W), 16 Nov 2004, B. Gamboa (1♀; INBIO); Parque Nacional Marino Las Baulas, Playa Grande (10°19.3'N, 85°50.5'W; 0–100 m), 19-25 Aug 2003, D. Briceño (2♂, 5♀; INBIO); Playa Tamarindo (10°17.8'N, 85°50.6'W; swept from sandy mud flats around brackish lagoon), 28 Mar 1987, W. E. Steiner (11♂, 12♀; USNM); Playa Puerto Soley (11°02.5'N, 85°40.1'W; beach), 16 Jun 2003, D. and W. N. Mathis (15♂; USNM). Limón: R. V. S. Grandoca-Manzanillo, Playa Gandoca (09°35.9'N, 82°36.78'W; 0 m), 23 May 2004, Y. Cardenas (1♀; INBIO); Talamanca (Estación Gandoca; 09°37.4'N, 82°41.7'W), 23 May 2004, Y. Cardenas (1♂; INBIO); Westfalia (4 km S; 9°54.5'N, 82°59'W; beach), 27 Jun 2001, W. N. Mathis (4♂, 1♀; USNM). Puntarenas: Bahia Gigante (Rio Lajas; 9°53.8'N, 84°56'W; beach), 22 Jun 2001, D. and W. N. Mathis (16♂, 3♀; USNM); Cabuye (Rio Lajas; 9°37'N, 85°04.8'W), 20 Jun 2001, D. and W. N. Mathis (6♂, 1♀; USNM); Chomes (10°2.7'N, 84°54.7'W), 19 Jun 2001, W. N. Mathis (15♂, 3♀; USNM); Dominical (9°14.8'N, 83°51.4'W; 0–2 m), 11 Jun 2003, D. and W. N. Mathis (23♂; USNM); Drake (8°41.4'N, 83°40.1'W), 12 Aug 2001, D. and W. N. Mathis (2♂, 2♀; USNM); Isla Bejuco (10°00'N, 85°02'W; 0–24 m), 16 Oct 2003, W. Porras (1♀; INBIO); Montezuma (1 km S; 9°39'N, 85°04.3'W), 20 Jun 2001, D. and W. N. Mathis (1♀; USNM); Parrita (9°31.2'N, 84°19.6'W; 8-9 m), 10 Jun 2003, D. Mathis (1♀; USNM); Playa Manuel Antonio (6 km SE Puerto Quepos; 9°22.9'N, 84°08.7'W), 30 May 1988, J. M. Hill, J. M. Mitchell, J. M. Swearingen, W. E. Steiner (2♂, 1♀; USNM); Pochotal (9°31.4'N, 84°28.4'W; 0–2 m), 12 Jun 2003, D. and W. N. Mathis (13♂, 1♀; USNM); Puntarenas (9°58.5'N, 84°50.9'W), 18 Jun 2003, D. and W. N. Mathis (1♂, 1♀; USNM); San Pedrillo (8°37.2'N, 83°44.1'W), 12-15 Aug 2001, D. and W. N. Mathis (17♂, 1♀; USNM).

CUBA. Havana: Havana (beach; 23°5.8'N, 82°27.7'W), 2–14 Dec 1994, W. N. Mathis (1♂, 1♀; USNM). Sancti Spiritus: La Boca (4 km S; 21°45.9'N, 80°01.5'W), 12 Dec 1994, W. N. Mathis (3♂, 6♀; USNM); Playa Ancón (21°44.1'N, 79°59.9'W), 12 Dec 1994, W. N. Mathis (5♂, 1♀; USNM).

DOMINICA. Cabrits Swamp (15°35'N, 61°29'W), 1 Feb-19 Jun 1965, 1991, D. and W. N. Mathis, W. W. Wirth (6♂, 5♀; USNM); Grande Savane (pond margin), 20 Mar 1965, W. W. Wirth (15♂; USNM); Macoucheri (seashore), 1 Feb 1965, W. W. Wirth (1♂, 2♀; USNM).

DOMINICAN REPUBLIC. Azua: Puerto Viejo (18°20.9'N, 70°50.4'W), 14 May 1995, W. N. Mathis (2♂, 2♀; USNM). El Seibo: Rincón (near; 18°45.3'N, 68°55.7'W), 12 May 1995, W. N. Mathis (1♂; USNM). Independencia: near Jimani (18°32.6'N, 71°50.3'W), 20 May 1998, D. and W. N. Mathis (1♂; USNM); Mella (canal W; 18°21.5'N, 71°25.7'W), 20 May 1998, D. and W. N. Mathis (4♂, 3♀; USNM). La Altagracía: Bayahibe (18°22.3'N, 68°50.4'W), 13 May 1994, W. N. Mathis (3♂; USNM). La Romana: Isla Saona, Mano Juan (18°08.1'N, 68°44.5'W), 13 May 1995, W. N. Mathis (9♂, 1♀; USNM). La Vega: Salto Guasara (near Jarabacoa; 19°04.4'N, 70°42.1'W; 680 m), 9 May 1995, W. N. Mathis (1♀; USNM). Monte Cristi: Monte Cristi (beach; 19°51.5'N, 71°39.5'W), 18 May 1995, W. N. Mathis (1♂; USNM). Peravia: Rio Ocoa (San José Ocoa; 18°31.7'N, 70°30.4'W), 21 May 1998, D. and W. N. Mathis (1♂; USNM). Puerto Plata: Rio Camu (14 km E Puerto Plata; 19°11.9'N, 70°37.4'W), 17 May 1995, W. N. Mathis (1♂; USNM). San Cristobal: Rio Haina (18°25.9'N, 70°00.4'W), 27 May 1998, D. and W. N. Mathis (3♂; USNM); Nigua (18°22.4'N, 70°03'W), 27 May 1998, D. and W. N. Mathis (1♀; USNM).

ECUADOR. Guayas: Boliche (14.5 km S; 2°09.4'S, 79°35.5'W), 14 Jan 1978, W. N. Mathis (2♀; USNM). Manabi: Bahia de Caraquez (0°36.5'S, 80°25.8'W), 10 Jan 1978, W. N. Mathis (5♂, 22♀; USNM); Bahia de Caraquez (35.6 km E; 0°35.8'S, 80°13.5'W), 8-9 Jan 1978, W. N. Mathis (3♀; USNM).

GRAND CAYMAN. Airport (2 km E; 19°17.1'N, 81°21'W), 26 Apr 1994, W. N. Mathis (3♂, 5♀; USNM); Airport (W end; 19°17.4'N, 81°22.2'W), 26 Apr 1994, W. N. Mathis (1♀; USNM); Frank Sound Road (19°18.9'N, 81°10.9'W), 28 Apr 1994, W. N. Mathis (3♂, 3♀; USNM); George Town Harbour (19°18'N, 81°22.9'W), 28-29 Apr 1994, W. N. Mathis (1♂; USNM); Governor Gore Bird Sanctuary (19°16.7'N, 81°18.5'W), 25 Apr 1994, W. N. Mathis (9♂, 2♀; USNM); Heritage Beach (19°18'N, 81°9.8'W), 28 Apr 1994, W. N. Mathis (1♂, 1♀; USNM); Spotts (19°16.5'N, 81°19.1'W), 25 Apr 1994, W. N. Mathis (3♀; USNM).

GRENADA. St. George: Airport (Point Salines; 11°59.9'N, 61°46.1'W), 15 Sep 1996, W. N. Mathis (4♂, 2♀; USNM); Airport (Point Salines; 12°05'N, 61°46.9'W), 11-12 Sep 1997, W. N. Mathis (17♂, 2♀; USNM); Beauséjour Bay (12°05.5'N, 61°44.9'W), 21 Sep 1996, W. N. Mathis (1♂; USNM); True Blue Beach (11°59.9'N, 61°46.1'W), 15 Sep 1996, W. N. Mathis (1♀; USNM). St. Patrick: Bathway Beach (12°12.6'N, 61°36.7'W), 18-20 Sep 1996, 1997, W. N. Mathis (11♂, 2♀; USNM).

GUYANA. Hope Beach (6°44.7'N, 57°57.3'W), 22 Apr 1994, 1995, W. N. Mathis (12♂, 13♀; USNM); Mahaica (6°42.8'N, 57°55.6'W), 22 Apr 1995, W. N. Mathis (15♂, 5♀; USNM); Mahaica (3 km W; 6°43.5'N, 57°56.6'W), 14 Apr 1994, W. N. Mathis (3♂, 1♀; USNM).

HAITI. Baie de Chouchou (19°49'N, 72°28.5'W), 8 Jun 1978, L. Raccurtt, R. Lowrie (1♂; USNM).

HONDURAS. Cortés: Puerto Cortés/Omoa (15°49'N, 87°56.2'W), 26 Sep 1995, D. and W. N. Mathis (1♂; USNM).

JAMAICA. Clarendon: Farquhars Beach (17°50.9'N, 77°22.8'W), 9 May 1996, D. and W. N. Mathis, H. B. Williams (11♂, 12♀; USNM); Grantham (18°09.3'N, 77°23.8'W; 340 m), 16 Apr 2000, W. N. Mathis (1♂; USNM); Milk River Bath (mangroves), 11 Mar 1970, T. Farr, W. W. Wirth (4♂, 2♀; USNM); Portland Cottage (17°45.4'N, 77°11'W), 13 May 1996, D. and W. N. Mathis, H. B. Williams (1♂; USNM); Rest (3.5 km N; 17°54.1'N, 77°21.1'W), 9 May 1996, D. and W. N. Mathis, H. B. Williams (3♂, 1♀; USNM). Manchester: Alligator Pond (17°52.1'N, 77°33.9'W), 8 May 1996, D. and W. N. Mathis, H. B. Williams (10♂, 6♀; USNM); near Mandeville (18°03.5'N, 77°31.9'W), 15-18 Apr 2000, W. N. Mathis (1♀; USNM). Portland: Berridale (18°06.5'N, 76°20'W), Rio Grande River, 25 Apr 2000, W. N. Mathis (1♂; USNM); Long Bay (2.3 km W; 18°06.5'N, 76°20'W), 24 Apr 2000, W. N. Mathis (1♂; USNM); Reach (4 km N; 18°03.6'N, 76°20.4'W), 15 May 1996, D. and W. N. Mathis, H. B. Williams (4♂; USNM). St. Andrew: Mavis Bank (1.7 km E; 18°02.4'N, 77°39.5'W; 575 m), Yallahs River, 21-22 Apr-1 May 2000, W. N. Mathis (1♂; USNM); Mavis Bank (4.3 km SE; 18°01.4'N, 76°38.1'W; 480 m); Yallahs River, 22-23 Apr 2000, W. N. Mathis (4♂, 2♀; USNM). St. Elizabeth: Black River (18°01.4'N, 77°51.1'W), 11 May 1996, D. and W. N. Mathis, H. B. Williams (1♂, 2♀; USNM); Brae River (18°05.2'N, 77°39.3'W), 10 May 1996, D. and W. N. Mathis, H. B. Williams (2♂; USNM); Brae River (2 km S; 18°04.2'N, 77°39.5'W), 10 May 1996, D. and W. N. Mathis, H. B. Williams (5♂, 2♀; USNM); Elim (18°07.1'N, 77°40.6'W), 10 May 1996, D. and W. N. Mathis, H. B. Williams (2♀; USNM). St. Catherine: Port Henderson (17°56'N, 76°53'W; bay shore), 24 Feb 1969, W. W. Wirth (2♀; USNM). St. Thomas: Rozelle (17°52.3'N, 76°27.7'W), 14 May 1996, D. and W. N. Mathis, H. B. Williams (1♀; USNM); Yallahs River (mouth; 17°53'N, 76°35.6'W), 14 May 1996, D. and W. N. Mathis, H. B. Williams (1♂; USNM). Westmoreland: Negril Beach (18°16.4'N, 78°21.2'W; mangrove, rocky shore), 12 Mar 1970, W. W. Wirth (3♀; USNM); Savanna-La-Mar (18°13'N, 78°08'W), 13 Mar 1970, W. W. Wirth (7♂, 4♀; USNM).

MEXICO. Chiapas: Boca de Cielo (17 km S Puerto Arista; 15°51.1'N, 93°40.4'W), 18 May 1985, A. Freidberg, W. N. Mathis (1♀; USNM); Puerto Arista (2 km E; 15°58.5'N, 93°41.1'W), 18 May 1985, A. Freidberg, W. N. Mathis (4♂, 5♀; USNM).

PANAMA. Canal Zone: Kobbe Beach (Playa Bonita; 8°53.9'N, 79°34.7'W), Jul 1967, W. W. Wirth (1♂, 6♀; USNM). Coclé: Playa Santa Clara (8°22.4'N, 80°06.4'W), 2 Jul 1967, W. W. Wirth (7♂, 21♀; USNM).

PUERTO RICO. Arecibo (beach; 18°28.7'N, 66°42'W), 23 Sep 1995, D. and W. N. Mathis (1♂, 1♀; USNM); Fajardo, Las Croabas (Seven Seas Beach; 18°23'N, 65°37'W), 17 Feb 1996, W. E. Steiner, J. M. Swearingen (1♂; USNM); Playa de Guayanilla (18°0.4'N, 66°46.1'W), 19 Sep 1995, D. and W. N. Mathis (11♂, 1♀; USNM); Playa de Ponce (17°58.9'N, 66°37.2'W), 26 Apr 1954, H. J. Teas (1♂, 1♀; USNM).

ST. LUCIA. Castries (5 km S; 13°59'N, 60°00'W), 16 Jun 1991, D. and W. N. Mathis (2♂, 4♀; USNM); Dauphin Boguis (1.6 km S Marquis; 14°01'N, 60°55'W), 17 Jun 1991, D. and W. N. Mathis (1♀; USNM); Fond St. Jacques (13°50'N, 61°02'W), 13-14 Jun 1991, W. N. and D. Mathis (1♂; USNM); Laborie (5 km W; 13°49'N, 60°54'W), 15 Jun 1991, D. and W. N. Mathis (3♂, 3♀; USNM); Soufrière (beach; 13°51'N, 61°54'W), 11-12 Jun 1991, W. N. and D. Mathis (1♀; USNM); Sulphur Spring (13°50'N, 61°03'W), 14 Jun 1991, D. and W. N. Mathis (1♂, 4♀; USNM).

ST. VINCENT. Charlotte: Spring (13°11.1'N, 61°08.5'W), 6 Sep 1997, W. N. Mathis (4♂, 1♀; USNM). St. Andrew: Buccament Bay (near beach; 13°11'N, 61°16'W), 25-28 Mar 1989, W. N. Mathis (20♂, 21♀; USNM); Layou (13°12'N, 61°17'W), 8 Jun 1991, D. and W. N. Mathis (3♀; USNM). St. George: Yambou Head, 27 Mar 1989, W. N. Mathis (2♂; USNM). St. Patrick: Cumberland Bay (13°16'N, 61°16'W), 15 Sep 1997, W. N. Mathis (10♂, 9♀; USNM).

TRINIDAD AND TOBAGO. Tobago. St. Patrick: Pigeon Point (beach; 11°9.7'N, 60°50'W), 19 Apr 1994, D. and W. N. Mathis (13♂, 7♀; USNM). St. Paul: Delaford, Kings Bay (11°16'N, 60°32.8'W), 13 Jun 1993, W. N. Mathis (5♂, 1♀; USNM); Kendall (11°14.3'N, 60°35.7'W), 21 Apr 1994, W. N. Mathis (2♂, 1♀; USNM). Trinidad. St. Andrew: Lower Manzanilla (12 km S; 10°24'N, 61°02'W), bridge over Nariva River, 20-27 Jun 1993, W. N. Mathis (2♂, 5♀; USNM); Lower Manzanilla (16 km S; 10°22'N, 61°01'W), 20 Jun 1993, W. N. Mathis (9♂, 5♀; USNM). St. Patrick: Pitch Lake (10°14'N, 61°38'W), 24 Jun 1993, W. N. Mathis (2♀; USNM).

**Figure 28.**
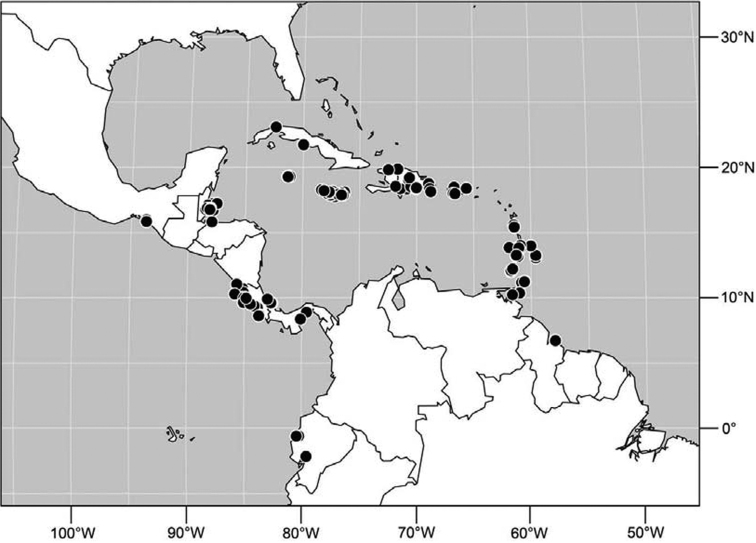
Distribution of *Polytrichophora adarca* sp. n.

Distribution (Fig. 28). Neotropical: Belize, Costa Rica (Guanacaste, Limón, Puntarenas), Ecuador (Guayas, Manabi), Guyana, Honduras (Cortés), Mexico (Chiapas), Panama, Trinidad and Tobago, West Indies (Barbados, Cuba, Dominica, Dominican Republic, Grand Cayman, Grenada, Haiti, Jamaica, Puerto Rico, St. Lucia, St. Vincent).

#### Remarks.

Although similar to *Polytrichophora conciliata* and *Polytrichophora marinoniorum* in having a row of setulae along the anteroventral surface of the forefemur and in having a small H-shaped hypandrium in ventral view, this species is distinguished from these congeners in having a shortened ventral epandrial process (Fig. 24).

### Polytrichophora agens

Cresson

http://species-id.net/wiki/Polytrichophora_agens

**Figures 29–31.**
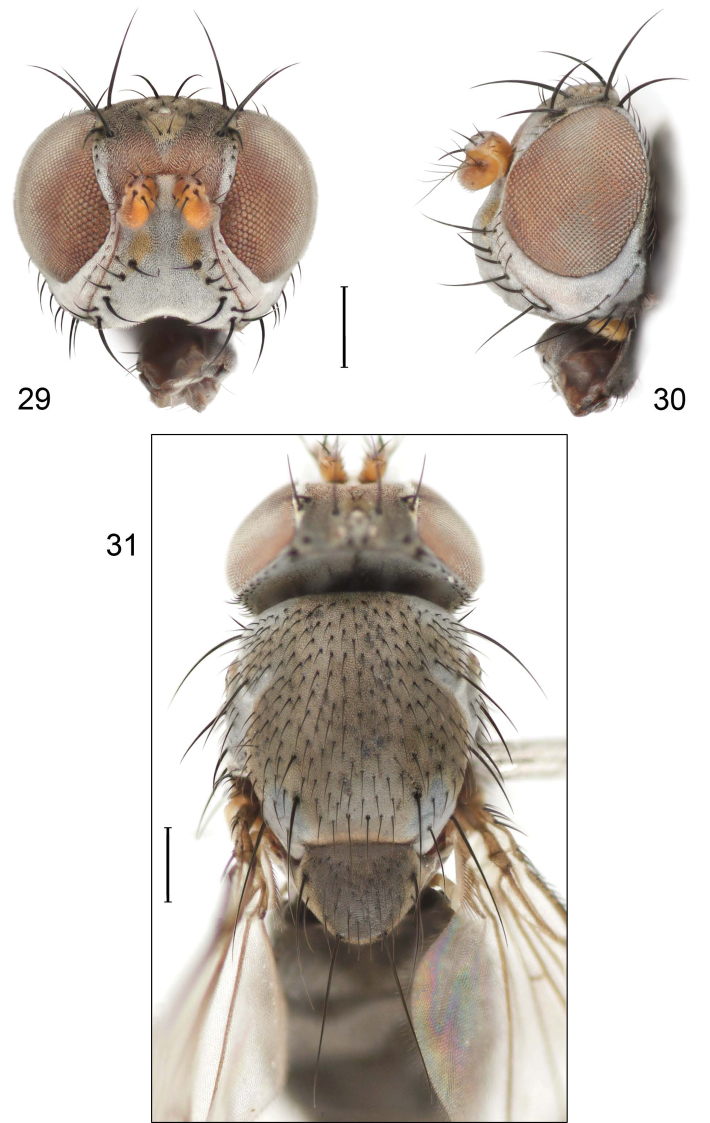
Illustration of *Polytrichophora agens* Cresson (male) (Grand Cayman. Governor Gore Bird Sanctuary (19°16.7'N, 81°18.5'W)) **29** head, anterior view **30** same, lateral view **31** thorax, dorsal view.

Figs 29 [Fig F13] 37

Polytrichophora agens Cresson 1924: 161; 1942: 114 [review of Nearctic fauna]. Wirth 1956: 9 [list, Bahamas]; 1965: 740 [Nearctic catalog]; 1968: 8 [Neotropical catalog]. Mathis and Zatwarnicki 1995: 183–184 [world catalog]. Mathis 1997b: 36–39 [review, Belize].

#### Diagnosis.

This species is distinguished from congeners by the following combination of characters: Moderately small shore flies, body length 2.30–2.90 mm (comparatively large and robust in comparison to congeners).

*Head* (Figs 29–30): Frons dull, heavily microtomentose, 2-toned, mostly tan with some faint golden reflections on posterior 2/3, anterior 1/3 (from level of fronto-orbital setae anteriad), gray. Antenna mostly yellow to yellowish orange, anterior portion of pedicel and basodorsal area of basal flagellomere with some blackish coloration; arista with 5 dorsal rays. Face at narrowest point about equal to combined length of pedicel and basal flagellomere; face densely microtomentose with subshiny to pearly luster, mostly white but with considerable gold coloration in antennal grooves and extended laterally onto parafacial; parafacial color not markedly differing from midfacies; parafacial becoming much wider ventrally and with slight to considerable ventral dilation, 2–3 times wider ventrally than dorsally; gena moderately high, height about equal to combined length of pedicel and basal flagellomere; gena-to-eye ratio 0.20–0.23.

*Thorax* (Fig. 31): Mesonotum mostly dull, densely microtomentose, concolorous with posterior 2/3 of frons; pleural area blackish gray. Anterior margin of wing lacking spine-like setae; costal vein ratio 0.54–0.58; M vein ratio 0.55–0.58. Forefemur lacking a row of 9–10 short, stout setulae along apical half of anteroventral surface; forefemur with a row of 7–9, of mostly elongate, well-developed, evenly spaced posteroventral setae, each equal to width of femur; tibiae mostly yellowish; basal tarsomeres yellow, apical 1–2 brown.

**Figures 32–36. F13:**
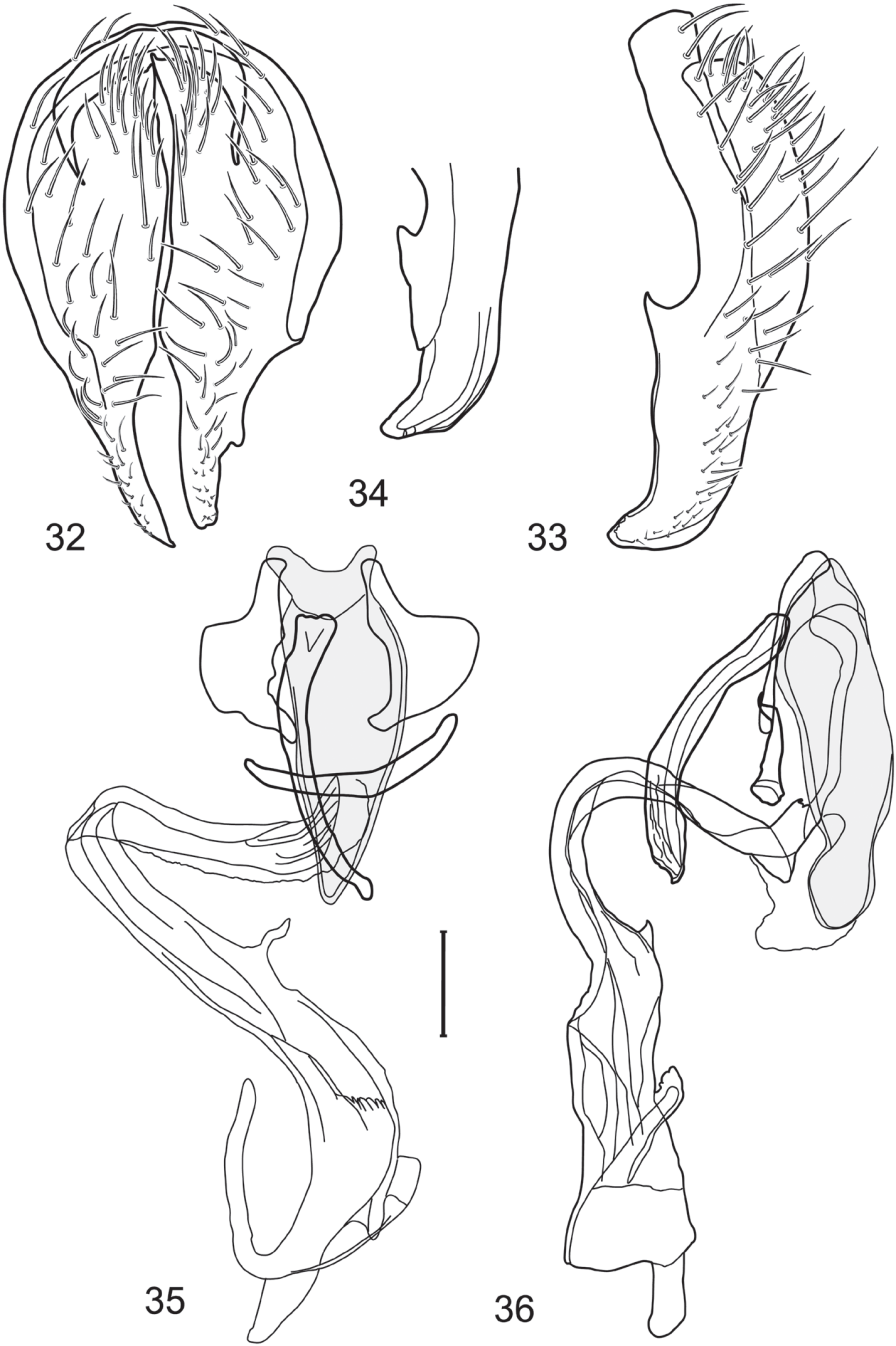
Illustration of *Polytrichophora agens* Cresson (male) (Florida. Monroe: Big Pine Key) **32** epandrium and cerci, posterior view **33** same, lateral view of left side **34** anterior portion of epandrium, lateral view of right side **35** internal structures of male terminalia (aedeagus [shaded], phallapodeme, gonite, hypandrium), ventral view **36** same, lateral view. Scale bar = 0.1 mm.

*Abdomen*: Tergites dull to subshiny, gray to blackish brown; tergites 1–4 bicolored, mostly blackish brown dorsally, gray lateroventrally; tergites 3–4 of male subequal, 4th not unusually longer, tergite 3 not produced ventrolaterally; dorsum of tergite 5 of male not darker than preceding tergites and usually with distinct, semicircular, grayish areas laterally; tergite 5 of male in dorsal view not markedly narrowed posteriorly, bearing row of 6–10, distinctly larger setae along extreme posterior margin with posterodorsal orientation; tergite 5 of male also gray on posterior 1/2–1/3; male sternite 1 transversely rectangular; sternite 2 much longer than wide, narrowly triangular, wider anteriorly, anterior margin emarginate; sternite 3 longer than wide, more robust than sternite 2, lateral margins shallowly arched, nearly parallel sided; sternite 4 robustly T-shaped, with posterior, transverse bar; sternite 5 as 2 , narrow, bar-like sternites. Male terminalia (Figs 32–36): Many structures asymmetrical; epandrium asymmetrical (Fig. 32), connected dorsally above cerci, in posterior view (Fig 32) tapered ventrally to an acutely pointed apex under which lies cordate-shaped, laterally flanged anterior margin (Fig. 32); epandrium in lateral view (Fig. 33) slightly curved anteroventrally, pointed apically, and with subapical ledge that also ends ventrally in a point; gonite in lateral view (Fig. 36) mostly rod-like, obtusely angled, end toward aedeagal base slightly enlarged, clavate; aedeagus with distinct basiphallus and distiphallus, in lateral view (Fig. 36) basiphallus somewhat tubular but lacking digitiform projection at apex, in ventral view (Fig. 35) narrowly cordate; distiphallus more membranous, contorted and sinuous, asymmetrically bifurcate apically (Figs 35–36); phallapodeme in lateral view (Fig. 36) as a curved, narrow, rod with keel essentially undeveloped, in ventral view (Fig. 35) asymmetrical, as a curved rod; hypandrium in lateral view (Fig. 36) short, rod-like, enlarged toward anterior margin, in ventral view (Fig. 35) transversely crescent shaped, narrow.

#### Type material.

The holotype female is labeled “Galveston J[u]n[e] [19]00 Tex[as]/W. M. Wheeler Collection./Holo-TYPE Polytrichophora AGENS E. T. Cresson Jr [maroon; species name handwritten]. The holotype is double mounted (pin in a rectangular card), is in good condition, and is deposited in the AMNH.

#### Type locality.

United States. Texas. Galveston (29°18.1'N, 94°47.9'W).

#### Other specimens examined.

Nearctic. UNITED STATES. DELAWARE. **Sussex:** Rehoboth (38°43.2'N, 75°04.6'W), 25 Jun 1939, A. L. Melander (1♀; ANSP).

FLORIDA. **Lee:** Fort Myers (26°38.4'N, 81°52.3'W), 24 Apr 1930, A. L. Melander (1♂; ANSP). **Monroe:** Bahia Honda Key (24°40.1'N, 81°15.9'W; seashore), 11 Apr 1970, W. W. Wirth (3♂, 2♀; USNM); Big Pine Key, Long Beach (24°38.1'N, 81°21.6'W), 11 Feb 2000, D. and W. N. Mathis (20♂, 5♀; USNM); Flamingo, Everglades National Park (25°18.8'N, 80°56.2'W), 25 Jan 1939, A. L. Melander (1♂, 2♀; ANSP); Layton (Long Key; (24°49.9'N, 80°48.3'W), 10 Feb 2000, D. and W. N. Mathis (1♀; USNM); Sugarloaf Key (24°38.6'N, 81°30.9'W), 11 Feb 2000, D. and W. N. Mathis (2♂, 1♀; USNM).

MISSISSIPPI. **Jackson:** Ocean Springs, Marsh Point, Gulf Coast Research Laboratory (30°23.6'N, 88°47.8'W), 4–6 Jun 1962, D. L. Deonier (16♂, 165♀; USNM).

NORTH CAROLINA. **Brunswick:** Southport (33°55.3'N, 78°01.2'W), 19 Apr 1989, D. and W. N. Mathis (7♀; USNM).

TEXAS. **Aransas:** Aransas State Park (28°08.1'N, 96°58.9'W), 20 May 1972, W. W. Wirth (1♀; USNM). **Galveston:** Galveston (29°18.1'N, 94°47.9'W), Jan 1900, W.M. Wheeler (1♂, 1♀; ANSP); Galveston Island (29°10'N, 94°05'W), 14 May 1993, D. and W. N. Mathis (1♂; USNM).

VIRGINIA. **Accomack:** Assateague Island, Toms Cove (37°53.3'N, 75°20.6'W), 20 Sep 2007, D. and W. N. Mathis (1♀; USNM); Assateague Island, wildlife loop (37°54.6'N, 75°21.1'W), 20 Sep 2007, D. and W. N. Mathis (1♂; USNM).

Neotropical. BAHAMAS. **Andros:** Fresh Creek (24°44'N, 77°47'W), 23 Apr 1953, E. B. Hayden (1♀; AMNH). **Grand Bahamas Island:** West End (26°41'N, 78°58'W), 12 May 1953, E. B. Hayden, L. Giovannoli, G. B. Rabb (1♀; AMNH). **Rum Cay:** near Port Nelson (23°39.6'N, 74°49.8'W), 16 Mar 1953, E. B. Hayden, L. Giovannoli (2♀; AMNH).

BELIZE. **Stann Creek:** Bread and Butter Cay (16°45'N, 88°09'W), Mar 1988, W. N. Mathis (1♀; USNM); Hopkins (16°52'N, 88°17'W), 3 Apr 1993, W. N. Mathis (2♂, 1♀; USNM); Man of War Cay (17°13'N, 87°54'W), Nov 1987, W. N. and D. Mathis (1♀; USNM); Round Cay (near Coco Plum Cay; 16°52'N, 88°07'W), Mar 1988, W. N. Mathis (1♂, 1♀; USNM); Stewart Cay (16°46'N, 88°09'W), 9 Nov 1987, W. N. Mathis (1♂, 1♀; USNM); Twin Cays (Aanderaa Flats, dock area, east shore of East Island, mud flat near Lair Channel, south end of West Island, West Bay; 16°49.9'N, 88°06.1'W), Mar-Nov 1987, 1988, W. N. and D. Mathis (35♂, 29♀; USNM); Wee Wee Cay (16°45.9'N, 88°08.6'W), Mar-Nov 1987, 1988, W. N. and D. Mathis (2♂, 1♀; USNM).

CUBA. **Havana:** Playa Jibacoa (23°08.9'N, 81°51'W), 26 Apr 1983, W. N. Mathis (2♀; USNM); Puerto Escondido (23°08.7'N, 81°42.5'W), 26 Apr 1983, W. N. Mathis (1♀; USNM)). **Sancti Spiritus:** La Boca (4 km S; 21°45.9'N, 80°01.5'W), 12 Dec 1994, W. N. Mathis (3♂, 6♀; USNM).

DOMINICAN REPUBLIC. **Azua:** Puerto Viejo (18°20.9'N, 70°50.4'W), 14 May 1995, W. N. Mathis (8♂, 4♀; USNM). **Barahona:** Barahona (18°12'N, 71°5.3'W), 20 May 1998, D. and W. N. Mathis (7♂, 4♀; USNM). **La Altagracía:** Bayahibe (18°22.3'N, 68°50.4'W), 13 May 1994, W. N. Mathis (3♂, 5♀; USNM). **La Romana:**Isla Saona, Catuano (18°11.7'N, 68°46.8'W), 13 May 1995, W. N. Mathis (4♂, 2♀; USNM); Isla Saona, Mano Juan (18°08.1'N, 68°44.5'W), 13 May 1995, W. N. Mathis (11♂, 6♀; USNM). **Monte Cristi:** Monte Cristi (beach; 19°51.5'N, 71°39.5'W), 18 May 1995, W. N. Mathis (2♂; USNM). **Pedernales:** Lago Oviedo (N shore; 17°47'N, 71°22.5'W), 15 May 1995, W. N. Mathis (1♂, 3♀; USNM).

GRAND CAYMAN. Airport (2 km E; 19°17.1'N, 81°21'W), 26 Apr 1994, W. N. Mathis (2♂; USNM); Bodden Town (1.6 km E; 19°17.5'N, 81°13.3'W), Meagre Bay Pond, 29 Apr 1993, W. N. Mathis (4♂, 5♀; USNM); Double Head (19°23.4'N, 81°22.3'W), 27 Apr 1994, D. and W. N. Mathis (2♂, 5♀; USNM); Frank Sound Road (19°18.9'N, 81°10.9'W), 28 Apr 1994, W. N. Mathis (2♂, 4♀; USNM); George Town Harbour (19°18'N, 81°22.9'W), 28–29 Apr 1994, W. N. Mathis (9♂, 2♀; USNM); Governor Gore Bird Sanctuary (19°16.7'N, 81°18.5'W), 25 Apr 1994, W. N. Mathis (6♂, 1♀; USNM); Heritage Beach (19°18'N, 81°9.8'W), 28 Apr 1994, W. N. Mathis (3♂, 1♀; USNM); Spotts (19°16.5'N, 81°19.1'W), 25 Apr 1994, W. N. Mathis (11♂, 9♀; USNM).

JAMAICA. **Clarendon:** Barnswell Beach (17°45.'N, 77°08.5'W), 13 May 1996, D. and W. N. Mathis, H. B. Williams (13♂, 1♀; USNM); Farquhars Beach (17°50.9'N, 77°22.8'W), 9 May 1996, D. and W. N. Mathis, H. B. Williams (9♂, 1♀; USNM); Jackson Bay (17°44.7'N,77°12.6'W), 13 May 1996, D. and W. N. Mathis, H. B. Williams (5♂; USNM); Portland Cottage (1 km S; 17°45.8'N, 77°12.6'W), 13 May 1996, D. and W. N. Mathis, H. B. Williams (4♂, 1♀; USNM). **Manchester:** Alligator Pond (17°52.1'N, 77°33.9'W), 8 May 1996, D. and W. N. Mathis, H. B. Williams (1♂, 3♀; USNM). **St. Andrew:** Mavis Bank (1.7 km E; 18°02.4'N, 77°39.5'W; 575 m), Yallahs River, 21–22 Apr-1 May 2000, W. N. Mathis (2♂, 1♀; USNM); Mavis Bank (4.3 km SE; 18°01.4'N, 76°38.1'W; 480 m); Yallahs River, 22–23 Apr 2000, W. N. Mathis (1♂, 1♀; USNM). **St. Elizabeth:** Black River (18°01.4'N, 77°51.1'W), 11 May 1996, D. and W. N. Mathis, H. B. Williams (5♂, 4♀; USNM); Salt Pond, Parottee Beach (17°58.1'N, 77°50.2'W), 19 Apr 2000, W. N. Mathis (7♂, 3♀; USNM); Ys Falls (18°09.3'N, 77°49.5'W), 17–18 Apr 2000, W. N. Mathis (1♀; USNM). **Westmoreland:** Negril (S beach; 18°17.7'N, 78°21.4'W), 11 May 1996, D. and W. N. Mathis, H. B. Williams (1♀; USNM).

PUERTO RICO. Playa de Guayanilla (18°0.4'N, 66°46.1'W), 19 Sep 1995, D. and W. N. Mathis (1♀; USNM); Punta Jacinto (near Guanica; 17°57'N, 66°52.6'W), 20 Sep 1995, D. and W. N. Mathis (1♂, 1♀; USNM).

#### Distribution

**Figure 37.**
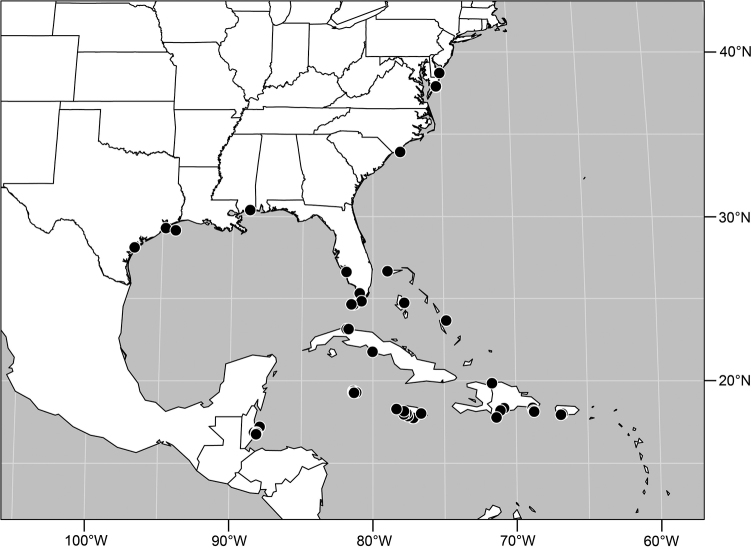
Distribution of *Polytrichophora agens* Cresson.

(Fig. 37). Nearctic: United States (Delaware, Florida, Mississippi, North Carolina, Texas, Virginia). Neotropical: Bahamas, Belize, West Indies (Cuba, Dominican Republic, Grand Cayman, Jamaica, Puerto Rico).

#### Natural history.

All specimens that we have examined are from localities that are associated with marine or at least brackish-water shorelines, indicating a tolerance and perhaps an affinity for these habitats.

#### Remarks.

Specimens of this species are usually relatively large and robust, especially the abdominal tergites. Unlike most congeners, there is some asymmetry in some structures of the male terminalia (similar to *Polytrichophora flavella*; Figs 32, 35). From similar congeners, this species is distinguished by the shortened apical arms (apparently secondarily shortened) of the H-shaped hypandrium.

### Polytrichophora arnaudorum sp. n.

urn:lsid:zoobank.org:act:8C68F454-E3FA-4598-8D50-B71F6CFDE7DA

http://species-id.net/wiki/Polytrichophora_arnaudorum

Figs 38 42

#### Diagnosis.

This species is distinguished from congeners by the following combination of characters: Small to moderately small shore flies, body length 1.90–2.40 mm.

*Head*: Frons largely dull, moderately microtomentose, 2-toned, posterior 2/3 mostly grayish to whitish tan, anterior 1/3 (from level of fronto-orbital setae anteriad) yellow to yellowish gray. Antenna mostly yellow to yellowish orange, basodorsal area of basal flagellomere with some blackish coloration; arista with 5 dorsal rays. Face at narrowest point about equal to combined length of pedicel and basal flagellomere; face densely microtomentose, microtomentum with faint shiny to pearly luster, mostly tannish white; parafacial and gena becoming more whitish than face; parafacial becoming much wider ventrally with slight to considerable ventral dilation; parafacial 2–3 times wider ventrally than dorsally; gena moderately high, height about equal to combined length of pedicel and basal flagellomere; gena-to-eye ratio 0.21–0.24.

*Thorax*: Mesonotum mostly dull to faintly subshiny, densely microtomentose, mostly tan to brown, becoming gray laterally and anteriorly; pleural area mostly gray. Anterior margin of wing lacking spine-like setae; costal vein ratio 0.58–0.72; M vein ratio 0.48–0.54. Forefemur lacking a row of 9–10 short, stout setulae along apical half of anteroventral surface; forefemur with a row of 4–5, of mostly elongate, well-developed, evenly spaced posteroventral setae, each equal to width of femur; tibiae mostly gray, only apices yellowish; basal tarsomeres yellow, apical 1–2 becoming darker, mostly brown.

**Figures 38–41.**
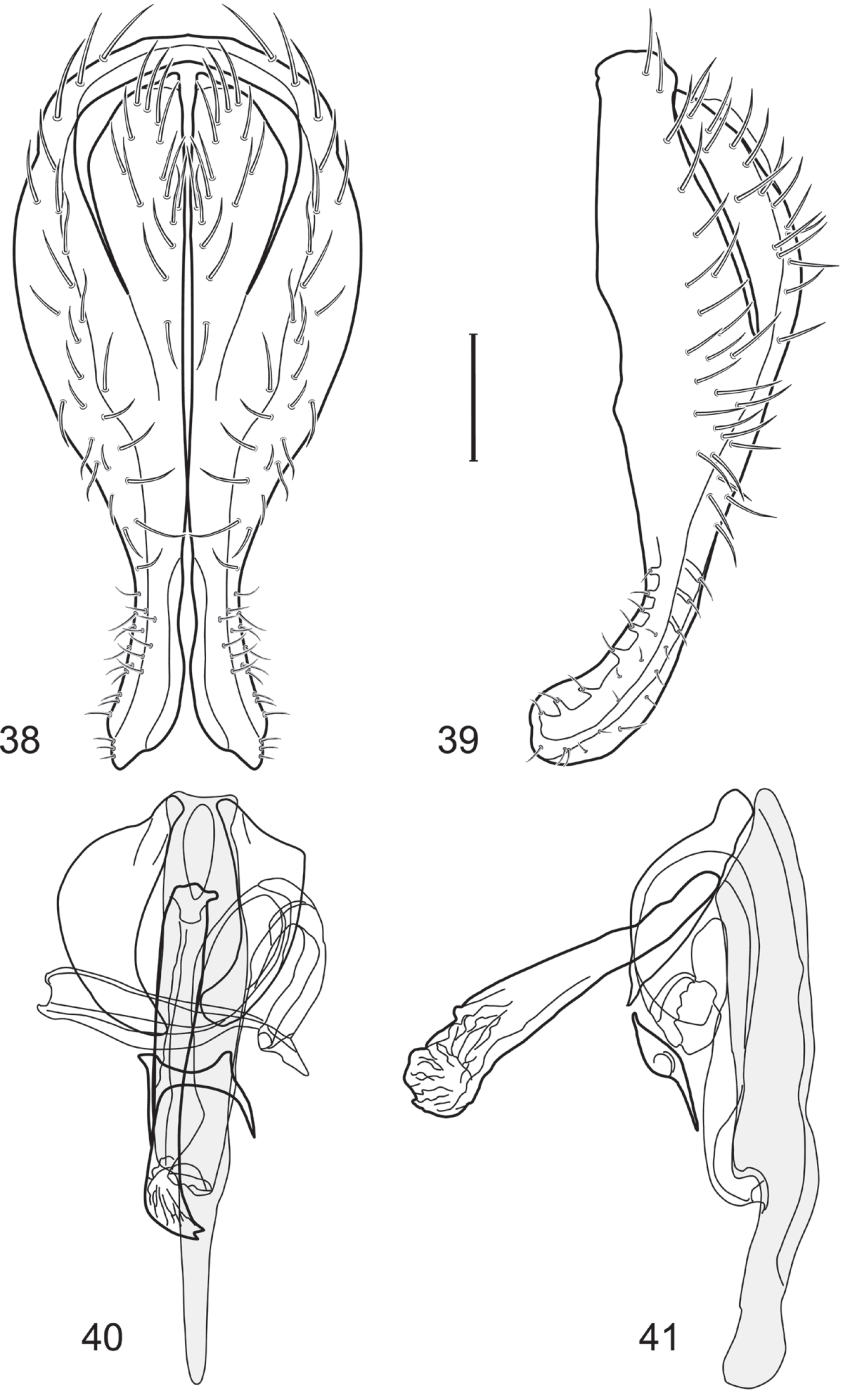
Illustration of *Polytrichophora arnaudorum* sp. n. (male) (Mexico. Baja California. San Felipe) **38** epandrium and cerci, posterior view **39** same, lateral view **40** internal structures of male terminalia (aedeagus [shaded], phallapodeme, gonite, hypandrium), ventral view **41** same, lateral view. Scale bar = 0.1 mm.

**Figure 42.**
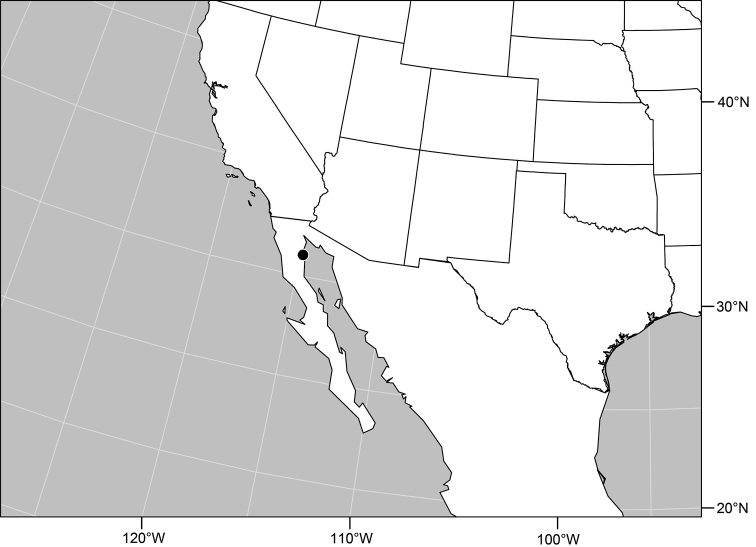
Distribution of *Polytrichophora arnaudorum* sp. n.

*Abdomen*: Male terminalia (Figs 38–41): Epandrium connected dorsally above cerci (Fig. 38), evenly setulose, in posterior view (Fig. 38) with dorsal 2/3 oval, ventral 1/3 distinctly narrowed, elongate, each extended ventral process robustly developed throughout length, lateral margins shallowly concave, tapered just before apex to acute point (Figs 38–39), ventral third of epandrium in lateral view (Fig. 39) shallowly curved anteriorly, ventral extension robustly developed, expanded gradually forming narrowly spatulate process, apex rounded; cerci large, prominent in lateral view; aedeagus complex (Figs 40–41) with distinct, narrow basiphallus and contorted, curved, more membranous distiphallus, apex of distiphallus lacking narrow projection; phallapodeme in lateral view (Fig. 41) rod-like, narrowly clavate, narrowed basally, very slightly sinuous, keel very shallowly developed, evident only toward end extended to hypandrium, in ventral view (Fig. 40) nearly straight except for curved apex; gonite in lateral view (Fig. 41) elongate, very narrow, conspicuously curved, in ventral view (Fig. 40) asymmetrically developed with extended flanges differing in shape on right and left sides; hypandrium in lateral view (Fig. 42) relatively short, in ventral view (Fig. 40) broadly H-shaped with posterior arms stubby, much shorter than anterior arms.

#### Type material.

The holotype male is labeled “San Felipe[,] Baja Cal.Mex[,] 19 Feb 1954[,] P.H.Arnaud/USNM ENT 00285968 [plastic bar code label]/HOLOTYPE ♂ *Polytrichophora arnaudorum* Mathis & Zatwarnicki, CAS [red].” The holotype is double mounted (glued to a paper triangle), is in excellent condition (apical portion of left arista missing), and is deposited in CAS. Twenty-three paratypes (6♂, 14♀; CAS, USNM) bear the same label data as the holotype. A male and female paratype (CAS) bear the same locality data as the holotype but with the date of 5 Mar 1963.

#### Type locality.

Mexico. Baja California. San Felipe (31°01.5'N, 114°50.4'W).

#### Distribution

(Fig. 42). Nearctic: Mexico (Baja California).

#### Etymology.

The species epithet, *arnaudorum*, is a plural genitive patronym to recognize and honor Paul H. Arnaud, Jr. and his wife, Madeline (nee Milliet), who contributed greatly to our careers in entomology and specifically to the study of Diptera. Paul was the collector of the type series.

#### Remarks.

This species is similar and probably closely related to *Polytrichophora conciliata*, *Polytrichophora adarca*, and *Polytrichophora sinuosa* but is distinguished from these species and other congeners in having a long anterior process of the aedeagus (basiphallus) (Figs 40–41).

### Polytrichophora barba sp. n.

urn:lsid:zoobank.org:act:B0A80F11-799A-4DA1-8984-F5FAB45FD8B7

http://species-id.net/wiki/Polytrichophora_barba

Figs 43 –47

Polytrichophora orbitalis of authors, not Loew [misidentification]. Woodley and Hilburn 1994: 31 [list, Bermuda].

#### Diagnosis.

This species is distinguished from congeners by the following combination of characters: Small to moderately small shore flies, body length 1.65–2.10 mm.

*Head*: Frons largely dull to faintly subshiny, moderately microtomentose, mostly grayish to brown, extreme anterior portion yellowish orange. Antenna mostly yellow to yellowish orange, arista with 5 dorsal rays. Face at narrowest point about equal to combined length of pedicel and basal flagellomere; face densely microtomentose, microtomentum with faint shiny to pearly luster, mostly yellow to faintly whitish yellow; parafacial and gena becoming slightly more whitish than face; parafacial becoming much wider ventrally with moderate ventral dilation; gena moderately short, height less than combined length of pedicel and basal flagellomere; gena-to-eye ratio 0.16–0.17.

*Thorax*: Mesonotum mostly dull to faintly subshiny, densely microtomentose, mostly tan to brown, becoming gray laterally and anteriorly; pleural area mostly gray. Anterior margin of wing lacking spine-like setae; costal vein ratio 0.48–0.68; M vein ratio 0.47–0.60. Forefemur lacking a row of 9–10 short, stout setulae along apical half of anteroventral surface; forefemur with a row of 4–5, of moderately well-developed, evenly spaced posteroventral setae on apical half, each seta less than width of femur; tibiae mostly gray, only apices yellowish; basal tarsomeres yellow, apical 1–2 becoming slightly darker, mostly tannish yellow.

**Figures 43–46.**
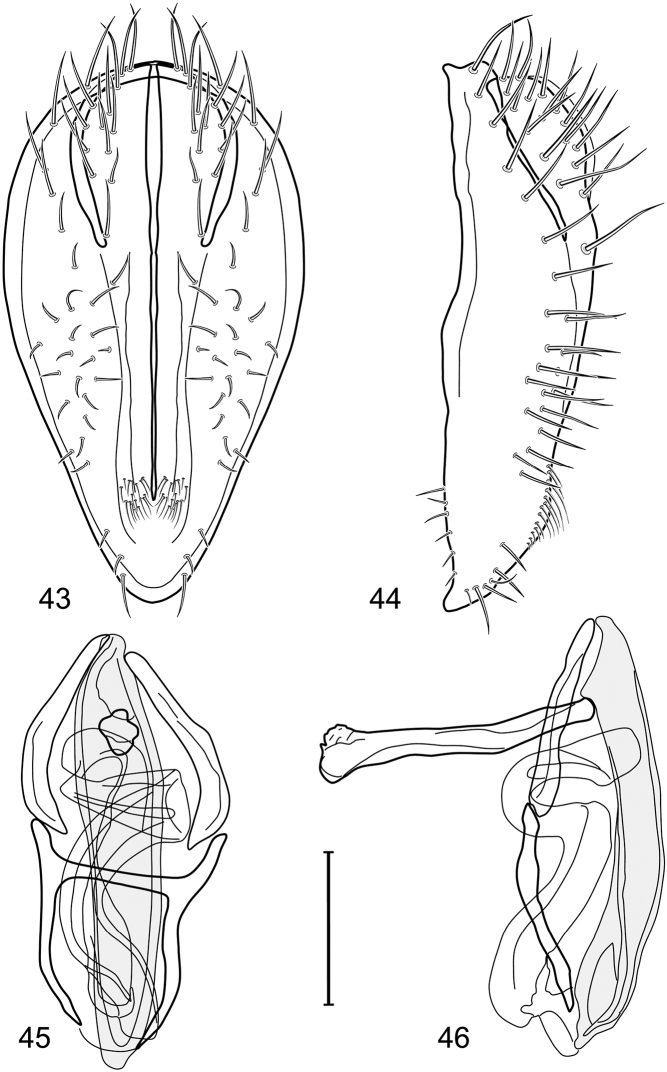
Illustration of *Polytrichophora barba* sp. n. (male) (Peru. Loreto: Granja UNAP, near Iquitos) **43** epandrium and cerci, posterior view **44** same, lateral view **45** internal structures of male terminalia (aedeagus [shaded], phallapodeme, gonite, hypandrium), ventral view **46** same, lateral view. Scale bar = 0.1 mm.

**Figure 47.**
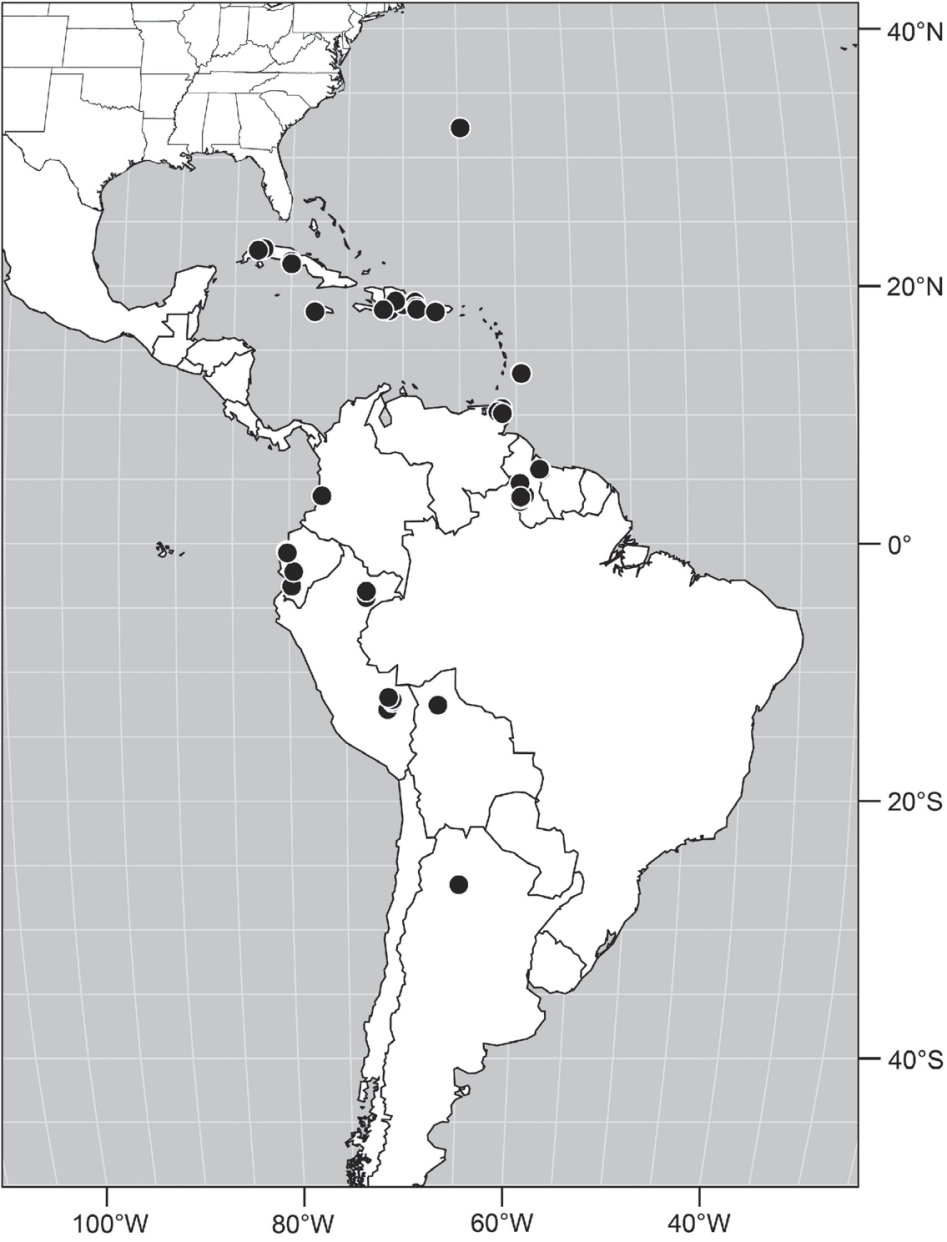
Distribution of *Polytrichophora barba* sp. n.

*Abdomen*: Tergites widely subshiny gray medially, laterally tan to tannish gray. Male terminalia (Figs 43–47): Epandrium in posterior view (Fig. 43) moderately broadly rounded dorsally, thereafter tapered ventrally to narrowly rounded ventral apex, in lateral view (Fig. 43) with posterior margin curved with slight prominence at ventral fourth that bears a more dense tuft of setulae, anterior margin irregularly straight; cerci moderately sized, relatively narrowly fused with epandrium; aedeagus in lateral view (Fig. 47) elongate, narrow, folded back on itself, basiphallus shallowly curved, parallel sided, distiphallus twisted, deeply recurved subapically; phallapodeme in lateral view rod-like, oriented almost perpendicular to general plane of terminalia, keel barely evident at hypandrial end, almost parallel sided, very shallowly angular, in ventral view (Fig. 46) as a small rounded structure with short dorsal and ventral knobs; gonite in lateral view (Fig. 47) narrow, elongate, nearly straight, rod-like, in ventral view (Fig. 46) slightly asymmetrical, elongate, more curved toward hypandrial end; hypandrium in lateral view (Fig. 47) nearly flat, very narrow and elongate, margins irregularly sinuous, in ventral view (Fig. 47) slightly asymmetrically H-shaped with bar situated much closer to posterior portion, anterior arms slightly longer than bar, pointed apically, posterior short, about half length of bar and slightly flared laterally.

#### Type material.

The holotype male is labeled “**CUBA.** Sanc[ti].Spiritus[,] Topes de Collantes[,] 21°54.4'N, 80°01.4'W, 670m, 9–11Dec1994[,] Wayne N. Mathis/HOLOTYPE ♂ *Polytrichophora barba* Mathis & Zatwarnicki, USNM [red]/USNM ENT 00133322 [plastic bar code label].” The holotype is double mounted (minuten pin in a block of plastic), is in excellent condition, and is deposited in the USNM. Twenty-six paratypes (14♂, 12♀; USNM) bear the same label data as the holotype. Other paratypes are as follows: CUBA. **Cienfuegos:** Topes de Collantes (5 km WNW; 21°56.5'N, 80°2.3'W; 600 m), 11 Dec 1994, W. N. Mathis (2♀; USNM).**Havana:** Ojo de Agua (22°54.6'N, 82°29.1'W), 8 Dec 1994, W. N. Mathis (3♀; USNM). **Pinar del Rio:** Soroa (22°47.7'N, 83°W), 4–6 Dec 1994, W. N. Mathis (4♂, 3♀; USNM).**Sancti Spiritus:** Playa Ancón (21°44.1'N, 79°59.9'W), 12 Dec 1994, W. N. Mathis (1♀; USNM).

#### Type locality.

Cuba. Sancti Spiritus: Topes de Collantes (21°54.4'N, 80°01.4'W, 670 m).

#### Other specimens examined.

Nearctic. BERMUDA. **Devonshire:** Devonshire Marsh (32°18'N, 64°45'W), 30 May 1991, W. N. Mathis (2♂, 2♀; USNM).

Neotropical. ARGENTINA. **Tucumán:** Padre Monti, Burruyacu (26°29'S, 64°58'W), 17 Jan 1948, R. Golbach (1♀; USNM).

BARBADOS. **St. Andrew:** Belleplaine (13°14.8'N, 59°33.6'W), 21 May-1 Sep 1996, 1997, D. and W. N. Mathis, H. B. Williams (7♂, 3♀; USNM); Walkers Bridge (13°15'N, 59°34.5'W), 11 Sep 1996, W. N. Mathis (1♀; USNM). **St. Joseph:** Joes River (13°12.8'N, 59°32.3'W), 10 Sep 1996, W. N. Mathis (2♂; USNM).

BOLIVIA. **Beni:** Mission Cavinas (12°31.8'S, 66°55'W), Jan, W. M Mann (1♂; USNM).

COLOMBIA. **Valle de Cauca:** Río Raposo (3°43'N, 77°08'W; light trap), Apr–Jul 1964, V. H. Lee (2♂, 2♀; USNM).

DOMINICAN REPUBLIC. **Barahona:** Cortico, La Mina (18°06.7'N, 71°13.4'W; 1300 m), 21 Mar 1999, W. N. Mathis (4♂; USNM). **Distrito Nacional:** Santo Domingo (Jardín Botanico; 18°32.5'N, 69°57'W), 26 Mar 1999, D. and W. N. Mathis (1♂, 1♀; USNM). **El Seibo:** El Seibo (5 km E; 18°44.73'N, 68°59.2'W; 120 m), 12 May 1995, W. N. Mathis (1♀; USNM); Rincón (near; 18°45.3'N, 68°55.7'W), 12 May 1995, W. N. Mathis (3♂, 2♀; USNM). **La Altagracía:** Bayahibe (18°22.3'N, 68°50.4'W), 13 May 1994, W. N. Mathis (2♂, 1♀; USNM). La Romana: Isla Saona, Catuano (18°11.7'N, 68°46.8'W), 13 May 1995, W. N. Mathis (1♂; USNM). **La Vega:** Constanza (ca. 16 km SE; 18°50.6'N, 70°40.7'W; 1580 m), 15 May 1998, D. and W. N. Mathis (1♀; USNM). **Pedernales:** Rio Mulito (21 km N Pedernales; 18°09.3'N, 71°45.6'W; 270 m), 18–20 Mar 1999, W. N. Mathis (2♂; USNM).

ECUADOR. **El Oro:** Pasaje (6 km E; 3°18.1'S, 79°47.1'W), 13 Jan 1978, W. N. Mathis (6♂, 4♀; USNM). **Guayas:** Boliche (14.5 km S; 2°10.5'S, 79°36.5'W), 14 Jan 1978, W. N. Mathis (2♂, 3♀; USNM). **Manabi:** Bahia de Caraquez (35.6 km E; 0°35.8'S, 80°13.5'W), 8–9 Jan 1978, W. N. Mathis (4♂, 5♀; USNM); Chone (0°41.7'S, 80°05.3'W), 9 Jan 1978, W. N. Mathis (2♂, 2♀; USNM); Chone (12 km W; Arabia; 0°42.8'S, 80°10.3'W), 8 Jan 1978, W. N. Mathis (1♂; USNM).

GUYANA. Dubulay Ranch, Aramatani Creek (5°40.9'N, 57°51.5'W), 9–11 Apr 1994, W. N. Mathis (1♂; USNM); Dubulay Ranch, Berbice River (5°40.9'N, 57°51.5'W), 9–11 Apr 1994, W. N. Mathis (7♂, 11♀; USNM); Karanambo (3°45.1'N, 59°18.6'W), 31 Mar 1994, W. N. Mathis (4♂, 8♀; USNM); Karanambo, Rupununi River (3°45'N, 59°17.5'W; 85 m; sand bank), 2 Dec 2010, W. N. Mathis (2♂; USNM); Karanambo, Rupununi River (ox bow; 3°45.1'N, 59°18.6'W), 2 Apr-13 Dec 1994, 2010, W. N. Mathis (10♂, 8♀; USNM); Kumu River and Falls (25 km SE Lethem in Kanuku Mountains; 3°15.9'N, 59°43.6'W), 28–30 Apr 1995, W. N. Mathis (1♀; USNM); Paramakatoi (04°42'N, 59°42.8'W), 24–25 Aug 1995, W. N. Mathis (1♀; USNM); Pirara Ranch and River (03°32.1'N, 59°40.5'W), 24–25 Apr 1995, W. N. Mathis (8♂, 4♀; USNM); Pirara Ranch, Cashew Lake (3°36.7'N, 59°40.5'W), 23–27 Apr 1995, W. N. Mathis (2♂; USNM); Wiruni River (5°46.6'N, 58°01'W), 11 Apr 1994, W. N. Mathis (3♂, 7♀; USNM).

JAMAICA. **St. Elizabeth:** Black River (18°01.4'N, 77°51.1'W), 11 May 1996, D. and W. N. Mathis, H. B. Williams (1♀; USNM).

PERU. **Cuzco:** Paucartambo, Atalaya (Río Alto Madre de Dios; 12°53.1'S, 71°21.6'W; 600 m), 4 Sep 1988, W. N. Mathis (1♀; USNM). **Loreto:** Rio Itaya (25 km S Iquitos; 04°11.3'S, 73°15'W), 22 Feb 1984, W. N. Mathis (1♀; USNM); Granja UNAP, near Iquitos (03°42'S, 73°13.7'W), 23 Feb 1984, W. N. Mathis (1♂; USNM). **Madre de Dios:** Diamante (Río Alto Madre de Dios; 12°19.9'S, 70°57.5'W; 400 m), 7 Sep 1988, W. N. Mathis (2♀; USNM); Quebrada Romero (near; Rio Manu; 12°07'S, 70°58'W), 8 Sep 1988, W. N. Mathis (1♂; USNM); Rio Manu, Pakitza (11°56.6'S, 71°16.9'W; 250 m), 9–23 Sep 1988, W. N. Mathis (19♂, 14♀; USNM).

PUERTO RICO. Laguna Cartagena (18°0.7'N, 67°06.4'W), 20 Jan 1954, J. Maldonado Capriles (1♂; USNM).

TRINIDAD and TOBAGO. Trinidad. **Caroni:** Talparo (2 km N, 10°31'N, 61°17'W), 22 Jun 1993, W. N. Mathis (1♂, 1♀; USNM). **St. Patrick:** Pitch Lake (10°14'N, 61°38'W), 24 Jun 1993, W. N. Mathis (1♀; USNM). **Victoria:** Basse Terre (7 km E; 10°07'N, 61°14'W), 27 Jun 1993, W. N. Mathis (3♂; USNM).

#### Distribution

(Fig. 47). Nearctic: Bermuda. Neotropical: Argentina (Tucumán), Bolivia (Beni), Colombia (Valle de Cauca), Ecuador (El Oro, Guayas, Manabi), Guyana, Peru (Cuzco, Loreto, Madre de Dios), Trinidad and Tobago, West Indies (Barbados, Cuba, Dominican Republic, Jamaica, Puerto Rico).

#### Etymology.

The species epithet, *barba*, is Latin word for beard and refers to the distinctive tuft of setulae, the beard, on the ventral portion of the epandrium of this species.

#### Remarks.

This species is similar to and has been confused with *Polytrichophora orbitalis* (see synonymy). Structures of the male terminalia are very distinctive, however, especially the tuft of short, setulae, which is apparently an autapomorphy, on the ventral portion of the epandrium (Figs 43-44). This tuft can often be seen in preserved specimens without dissection.

### Polytrichophora conciliata

Cresson

http://species-id.net/wiki/Polytrichophora_conciliata

Figs 48 52

Polytrichophora conciliata Cresson 1924: 161; 1942: 114 [review of Nearctic fauna]. Johnson 1925: 271 [list, Maine, Massachusetts]; 1930: 153 [list, Massachusetts]. Wirth 1965: 740 [Nearctic catalog]. Mathis and Zatwarnicki 1995: 184 [world catalog]. Mathis 1997b: 39 [review, Belize].

#### Diagnosis.

This species is distinguished from congeners by the following combination of characters: Small to moderately small shore flies, body length 1.70-2.15 mm.

*Head*: Frons dull, heavily microtomentose, 2-toned, mostly tan with some faint golden reflections on posterior 2/3, anterior 1/3 (from level of fronto-orbital setae anteriad), gray. Antenna mostly yellow to yellowish orange, anterior portion of pedicel and basodorsal area of basal flagellomere with some blackish coloration; arista with 5 dorsal rays. Face at narrowest point about equal to combined length of pedicel and basal flagellomere; face densely microtomentose, microtomentum with shiny to pearly luster, mostly white but with considerable gold coloration in antennal grooves and extended laterally onto parafacial; parafacial not markedly differing from midfacies in color, more golden dorsally, becoming whiter ventrally; parafacial with slight to considerable ventral dilation, 2–3 times wider ventrally than dorsally; gena relatively short, less than 1/4 eye height; gena-to-eye ratio 0.10–0.16. *Thorax*: Mesonotum mostly dull, densely microtomentose, concolorous with posterior 2/3 of frons; pleural area blackish gray. Stout setae on apical half; anterior margin of wing lacking large, spine-like setae; costal vein ratio 0.45–0.55; M vein ratio 0.55–0.59. Forefemur lacking row of 9–10 short, stout setae along apical half of anteroventral surface; forefemur with posteroventral row of 7–10 short setulae, each equal to width of femur; tibiae mostly yellowish; basal tarsomeres yellow, apical 1–2 brown.

**Figures 48–51.**
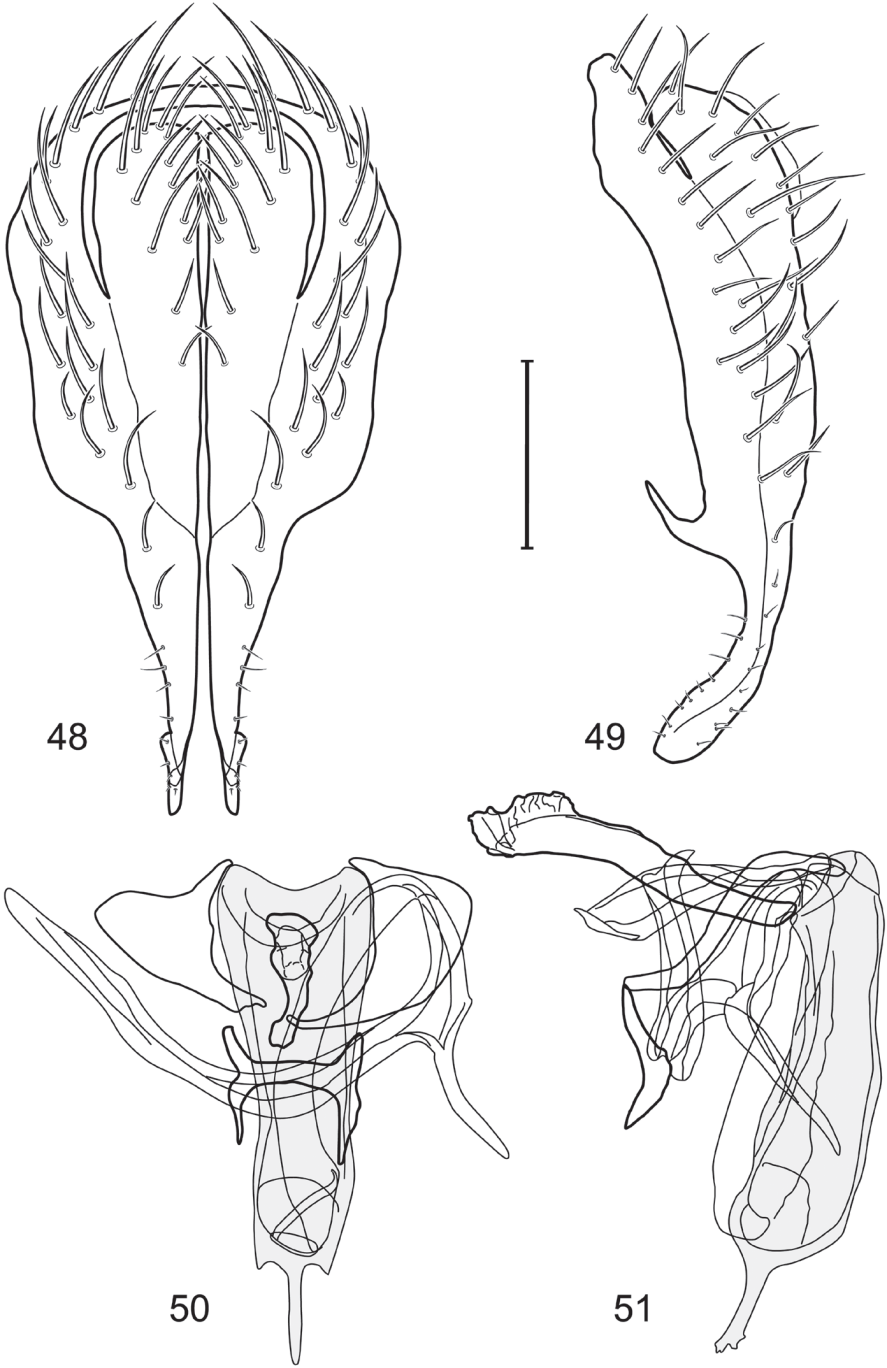
Illustration of *Polytrichophora conciliata* Cresson (male) (Florida. Lee Co., Ft. Myers Beach). **48** epandrium and cerci, posterior view **49** same, lateral view **50** internal structures of male terminalia (aedeagus [shaded], phallapodeme, gonite, hypandrium), ventral view **51** same, lateral view. Scale bar = 0.1 mm.

*Abdomen*: Tergites dull to subshiny, gray to blackish brown; tergites 1–4 bicolored, mostly blackish brown dorsally, gray lateroventrally; tergites 3–4 of male subequal, 4th not unusually longer, tergite 3 not produced ventrolaterally; dorsum of tergite 5 of male not darker than preceding tergites and usually with distinct, semicircular, grayish areas laterally; tergite 5 of male in dorsal view relatively blunt, somewhat truncate, bearing row of 6–10, distinctly larger setae subapically toward posterior margin with posterodorsal orientation; tergite 5 of male also gray on posterior 1/2–1/3; sternites 3–4 of male with dense row of setulae along posterior margin; sternite 5 as 2 subtriangular sternites. Male terminalia (Figs 48–51): Epandrium connected dorsally above cerci (Fig. 48), evenly setulose, in posterior view (Fig. 48) with dorsal 2/3 rectangular with rounded corners, ventral 1/3 distinctly narrowed, elongate, tapered ventrally to an acute point; ventral third of epandrium in lateral view (Fig. 49) shallowly curved anteriorly, apical 1/3 of ventral extension slightly expanded, narrowly spatulate, apex bluntly rounded; cerci large, prominent in lateral view; aedeagus complex with distinct basiphallus and twisted and curved distiphallus, apex of distiphallus (Figs 50–51) with elongate, parallel-sided, narrow projection; phallapodeme in lateral view (Fig. 51) rod-like, sinuous, keel shallowly developed, evident only toward end extended to hypandrium, in ventral view (Fig. 50) slightly contorted asymmetrically; gonite in lateral view (Fig. 51) elongate, narrow, parallel sided, conspicuously sinuous, almost S-shaped, in ventral view (Fig. 50) asymmetrically developed with extended flanges differing in shape; hypandrium in lateral view (Fig. 51) relatively short, in ventral view (Fig. 50) broadly H-shaped.

#### Type material.

The holotype male is labeled “Wildwood[,]N[ew]J[ersey][,] VII, 18, 1908 [18 Jul 1908]/♂/TYPE No. 6293 [magenta; number handwritten]/TYPE Polytrichophora CONCILIATA E.T.Cresson,Jr. [magenta; species name handwritten].” The holotype is double mounted (minuten pin in rectangular card block), is in good condition, and is deposited in the ANSP (6293).

#### Type locality.

United States. New Jersey. Cape May: Wildwood (39°0.4'N, 74°47.5'W).

**Other specimens examined.** UNITED STATES. ALABAMA. **Mobile:** Coden (30°23'N, 88°14'W), 12 May 1993, D. and W. N. Mathis (8♂, 12♀; USNM); Dauphin Island (30°15'N, 88°11'W), 12 May 1993, D. and W. N. Mathis (2♂, 2♀; USNM).

DELAWARE. **Kent:** Bombay Hook National Wildlife Refuge (Raymond Pool; 39°15.6'N, 75°28.2'W), 18 May 2006, D. and W. N. Mathis (6♂, 2♀; USNM); Bombay Hook National Wildlife Refuge (Shearness Pool; 39°16.2'N, 75°28.1'W), 18 May 2006, D. and W. N. Mathis (7♂, 1♀; USNM); Port Mahon (39°11.4'N, 75°23.9'W; beach), 19 May 2006, D. and W. N. Mathis (12♂, 8♀; USNM); Woodland Beach (39°19.9'N, 75°28.3'W; beach), 18 May 2006, D. and W. N. Mathis (5♂, 1♀; USNM). **Sussex:** Dewey Beach (38°41.5'N, 75°05.5'W), 27 Aug 1972, L. V. Knutson (1♀; USNM); Lewes Angola Neck Park (38°40.3'N, 75°09.1'W), 21 Sep 2007, G. A. Foster, A. M. Welch (2♀; USNM); Rehoboth Bay, Lynch Thicket (38°37.6'N, 75°05.6'W), 6 Sep 2007, G. A. Foster, A. M. Welch (1♂, 1♀; USNM); Indian River Inlet (38°36.9'N, 75°04.3'W), 17 May 2006, D. and W. N. Mathis (12♂, 3♀; USNM); Lewes, Delaware Bay, Roosevelt Inlet (38°47'N, 75°08'W), 29 May 1992, H. B. Williams (1♂, 3♀; USNM); Lewes-Rehoboth Canal (38°47'N, 75°08'W), 29 May 1992, H. B. Williams (10♂, 9♀; USNM).

DISTRICT OF COLUMBIA. Kenilworth Park (38°55'N, 76°57'W), 31 May-1 Jun 1996, D. and W. N. Mathis (1♂, 2♀; USNM); Theodore Roosevelt Island (38°53.7'N, 77°03.7'W), 5-19 May 1991, H. Robinson (6♂, 2♀; USNM).

FLORIDA. **Brevard:** Cape Canaveral causeway (28°24.3'N, 80°38.6'W), 18 Jan 2009, D. and W. N. Mathis (1♀; USNM). **Monroe:** Big Pine Key, Long Beach (24°38.1'N, 81°21.6'W), 11 Feb 2000, D. and W. N. Mathis (21♂, 1♀; USNM); Layton (Long Key; 24°49.9'N, 80°48.3'W), 10 Feb 2000, D. and W. N. Mathis (1♂, 4♀; USNM); Lower Matecumbe Key (24°51.4'N, 80°43.7'W), 10 Feb 2000, D. and W. N. Mathis (2♀; USNM); Sugarloaf Key (24°38.6'N, 81°30.9'W), 11 Feb 2000, D. and W. N. Mathis (1♂, 1♀; USNM). **Miami-Dade:** Key Largo (25°05.2'N, 80°26.8'W)), 25 Jan 1932, A. L. Melander (1♀; ANSP). **St. Johns:** Crescent Beach (1.6 km W; 29°46.1'N, 81°15.2'W), 26 Mar-10 Apr 1984, B. A. Foote (2♂, 5♀; CMNH). **Wakulla:** Panacea (1.6 km E; 30°02.2'N, 84°23.3'W), 16 Apr 1989, B. A. Foote (1♂, 1♀; CMNH).

GEORGIA. **Chatham:** Savannah (32°05'N, 81°06'W), 25 Aug 1958, H. R. Dodge (1♂, 1♀; USNM); Tybee Island (32°0'N, 80°50.7'W), 20 Apr 1911 (2♀; ANSP). **Glynn:** St. Simon Island (31°13'N, 81°22.3'W), 22 Apr-12 May 1911, J. C. Bradley (2♂, 2♀; ANSP). **Liberty:** St. Catherines Island (31°42.3'N, 81°16.2'W), 21-23 Apr 1978, R. W. Matthews, A. Hook, J. Krispyn (1♀; USNM).

LOUISIANA. **Liberia:** Cypremort Point State Park (29°44.5'N, 91°51.3'W; beach), 7 Jun 2004, W. N. Mathis (14♂, 2♀; USNM).

MAINE. **Washington:** Machias (44°42.9'N, 67°27.7'W), Jul 1917 (1♀; ANSP).

MARYLAND. **Anne Arundel:** Edgewater (6 km S; 38°53'N, 76°33'W; Smithsonian Environmental Research Center), 4 Aug 1994, W. N. Mathis (4♂, 1♀; USNM). **Baltimore:** Baltimore (39°17.4'N, 76°36.7'W), 16 May 1938, E. G. Fisher (1♀; ANSP). **Calvert:** Calvert Beach (38°27.9'N, 76°28.6'W), 17 Sep 1989, J. M. Hill, W. E. Steiner, J. M. Swearingen (1♀; USNM); Scientists Cliffs (38°31'N, 76°30.8'W), 2 Jun 1977, W. N. Mathis (5♂, 34♀; USNM). **Kent:** East Neck Island (39°05.4'N, 76°13.4'W), 24 Apr 1938, E. G. Fisher (2♂, 4♀; ANSP). **Queen Anne'S:** Kent Narrows (38°58.1'N, 76°14.3'W), Jul 1954, M. R. Wheeler (1♂, 1♀; USNM). **Worcester:** North Ocean City (38°23'N, 75°03'W), 24-25 Jul 1993, W. E. Steiner (1♀; USNM).

MASSACHUSETTS. **Dukes:** Muskeget Island (41°20.1'N, 70°18'W), 14 Jul 1927, W. S. (1♀; ANSP). **Barnstable:** Wellfleet (41°56.1'N, 70°01.6'W), 29 Jun 1930, A. L. Melander (1♀; ANSP).

MICHIGAN. **Wayne:** Detroit (42°19.9'N, 83°02.7'W), 27 Apr 1938, G. C. Steyskal (1♀; ANSP).

MISSISSIPPI. **Issaquena:** Mahannah Wildlife Area (32°32.9'N, 90°52.4'W; 13 m), 9 Jun 2004, W. N. Mathis (1♂; USNM). **Jackson:** Gravel Pit near highway 90 (30°25'N, 88°48'W; clay bank), 9 Jun 1962, D. L. Deonier (2♂, 2♀; USNM); Ocean Springs, Marsh Point, Gulf Coast Research Laboratory (30°23.6'N, 88°47.8'W), 5-6 Jun 1962, D. L. Deonier (10♂, 17♀; USNM).

NEW JERSEY. **Atlantic:**Absecon (39°24.9'N, 74°29.6'W), 26 Sep 2003, D. and W. N. Mathis (5♂, 2♀; USNM). **Cape May:** Avalon (39°06.1'N, 74°43'W), 8 Aug 1909 (1♀; ANSP); Stone Harbor (39°02'N, 74°45.5'W), 17 Jul 1910 (1♂; ANSP); Wildwood (39°0.4'N, 74°47.5'W), 18 Jul 1908, E. T. Cresson, Jr. (2♂, 5♀; ANSP).

NEW YORK. **Kings:** Sheepshead Bay (40°35'N, 73°55.8'W), 3 Jun 1904 (1♀; ANSP). **Nassau:** Oak Island (40°37.7'N, 73°21.9'W), Jul (2♀; ANSP). **Suffolk:** Cold Spring Harbor, Long Island (40°52.3'N, 73°27.4'W), Aug, A. L. Melander (1♂, 2♀; ANSP). NORTH CAROLINA. **Hanover:** Wilmington, Cape Fear River (33°53.1'N, 78°0.8'W), 23 Apr 1985, M. W. LaSalle (5♂, 3♀; USNM).

OHIO. **Lake:** Mentor Marsh (41°43.7'N, 81°19.3'W), 16 Jun 1976, D. L. Deonier (2♂, 2♀; USNM).

SOUTH CAROLINA. **Charleston:** McClellanville (Wedge Plantation; 11.25 km NE; 33°10.2'N, 79°24.2'W), 3 Apr 1970, K. W. Simpson (1♂; USNM).

TEXAS. **Galveston:** Gilchrist (5 km N; 29°30'N, 94°25'W), 14 May 1993, D. and W. N. Mathis (12♂, 6♀; USNM).

VIRGINIA. **Accomack:** Assateague Island, near Refuge headquarters (37°54.5'N, 75°21.6'W), 24 Jun-3 Oct 1967, 2005, D. and W. N. Mathis, G. C. Steyskal (3♂, 2♀; USNM); Assateague Island, Toms Cove (37°53.3'N, 75°20.6'W), 20 Sep-4 Oct 2005, 2007, D. and W. N. Mathis (16♂, 2♀; USNM); Assateague Island, Toms Cove (37°53.1'N, 75°20.7'W), 15 Jun 2007, D. and W. N. Mathis (9♂, 3♀; USNM); Assateague Island, wildlife loop (37°54.6'N, 75°21.1'W), 15 Jun-20 Sep 2007, D. and W. N. Mathis (13♂; USNM); Chincoteague (37°55.4'N, 75°21.2'W), 15 Jun-20 Sep 2007, D. and W. N. Mathis (10♂, 1♀; USNM); Chincoteague (causeway; 37°56.1'N, 75°25.2'W), 16 Jun-19 Sep 2007, D. and W. N. Mathis (16♂, 3♀; USNM); Wallops Island (37°51.1'N, 75°28.3'W), 29 May 1913, W. L. McAtee (1♂; ANSP). **Essex:** Tappahannock (37°55.8'N, 76°51.4'W; Rappahannock River), 27 Apr 2007, D. and W. N. Mathis (1♀; USNM). **Lancaster:** Belle Isle State Park (Humphreys Picnic area; 37°46.4'N, 76°35.6'W; Rappahannock River), 28 Apr 2007, D. and W. N. Mathis (11♂, 2♀; USNM); Belle Isle State Park (Watch House; 37°46.4'N, 76°36.1'W; Rappahannock River), 28 Apr 2007, D. and W. N. Mathis (3♂, 3♀; USNM). **Northampton:** Kiptopeke (37°10'N, 75°59.1'W), 2-6 Oct 1986, 1987, J. M. Hill, W. E. Steiner (12♂, 3♀; USNM); Oyster (37°17.2'N, 75°55.4'W), 14 Jun 2007, D. and W. N. Mathis (10♂, 3♀; USNM); Portsmouth (36°50.1'N, 76°17.5'W), 29 Jun 1939, A. L. Melander (1♀; USNM). **Richmond:** Warsaw (37°57.4'N, 76°45.6'W), 26 Jul 1952, W. W. Wirth (1♀; USNM). **Smyth:** Saltville (36°52.9'N, 81°45.7'W), 4 May 1962, W. W. Wirth (1♀; USNM); Saltville (36°52.3'N, 81°46.4'W; 521 m), 22 Jun-24 Sep 2007, D. and W. N. Mathis (2♂; USNM). **Spotsylvania:** Rappahannock River (38°18.8'N, 77°32.5'W), 15 Apr 2006, D. and W. N. Mathis (1♂; USNM). **Stafford:** Aquia Harbour (38°27.9'N, 77°23.3'W), 15 May 2000, D. and W. N. Mathis (1♀; USNM); Aquia Harbour, Aquia Creek (38°27.8'N, 77°23.1'W), 12 Apr 2006, D. and W. N. Mathis (4♀; USNM); Aquia Harbour, Lions Park (38°27'N, 77°23.3'W), 4 Jul-4 Nov 2005, 2006, D. and W. N. Mathis (4♂, 2♀; USNM); Aquia Landing (38°23.2'N, 77°19'W), 14 Apr-5 Nov 2005, 2006, D. and W. N. Mathis (10♂, 2♀; USNM); Falmouth (38°19.2'N, 77°28.1'W; Rappahannock River; 9 m), 30 Jun 2007, D. and W. N. Mathis (1♀; USNM). **Westmoreland:** Colonial Beach (38°14.9'N, 76°57.6'W; Potomac River), 8 Jun 2007, D. and W. N. Mathis (1♂, 2♀; USNM). *York*: York River (37°14.5'N, 76°31.2'W), 21 May 1938, E. G. Fisher (2♂, 1♀; ANSP). **Independent City:** Fredericksburg (Rappahannock River; 38°18.3'N, 77°27.5'W), 14 Apr 2006, D. and W. N. Mathis (8♂, 2♀; USNM).

#### Distribution

**Figure 52.**
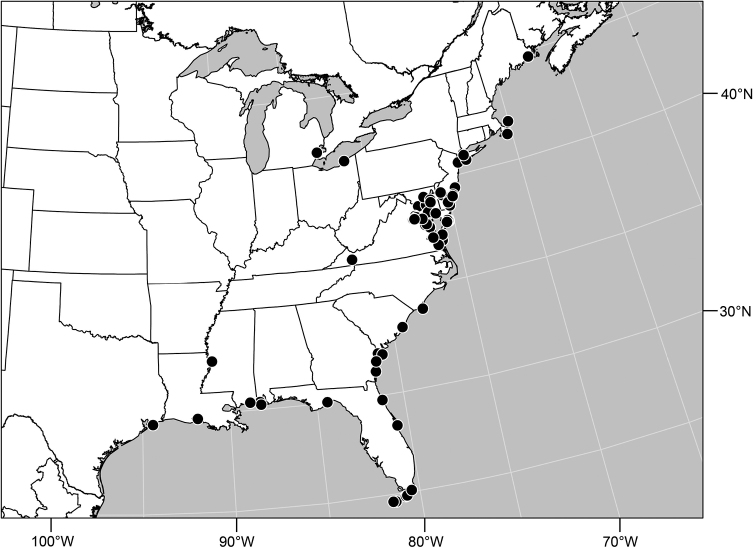
Distribution of *Polytrichophora conciliata* Cresson.

(Fig. 52). Nearctic: United States (Alabama, Delaware, District of Columbia, Florida, Georgia, Louisiana, Maine, Maryland, Massachusetts, Michigan, Mississippi, New Jersey, New York, North Carolina, Ohio, South Carolina, Texas, Virginia).

#### Remarks.

This species is similar to *Polytrichophora adarca* and *Polytrichophora marinoniorum* in having a row of short setulae along the anteroventral surface of the forefemur and subtlety differs from these two species in structures of the male terminalia, especially the ventral processes of the epandrium (perhaps equaling fused surstylus). Although there is some variation in the shape of these processes, males from the Atlantic coast of the United States have the apex in lateral view slightly enlarged apically (Fig. 49).

### Polytrichophora flavella sp. n.

urn:lsid:zoobank.org:act:67C3FD30-857B-4B6D-B1BF-14072E095C1A

http://species-id.net/wiki/Polytrichophora_flavella

Figs 53 58

#### Diagnosis.

This species is distinguished from congeners by the following combination of characters: Small to moderately small shore flies, body length 1.85–2.25 mm.

*Head*: Frons largely dull to faintly subshiny, brown with moderate investment of gray to yellowish gray microtomentum, anterior margin with some orange coloration; fronto-orbits narrow, gray to whitish gray. Antenna mostly yellow; basal flagellomere tannish yellow apicodorsally; arista with 5 dorsal rays. Face at narrowest point about equal to combined length of pedicel and basal flagellomere; face more densely microtomentose than frons, microtomentum with faintly shiny, mostly gray to whitish gray; parafacial and gena becoming slightly more whitish to silvery white than face; parafacial becoming wider ventrally with moderate ventral dilation; gena moderately high, height less than combined length of pedicel and basal flagellomere; gena-to-eye ratio 0.17–0.21.

*Thorax*: Mesonotum mostly dull to faintly subshiny, moderately densely microtomentose, mostly tan to brown, becoming gray laterally and anteriorly; pleural area mostly gray. Anterior margin of wing lacking spine-like setae; costal vein ratio 0.65–0.80; M vein ratio 0.55–0.64. Legs, including most of coxae, yellow; forefemur lacking a row of 9-10 short, stout setulae along apical half of anteroventral surface; forefemur with a row of 4–5, of moderately well-developed, evenly spaced, posteroventral setae on apical half, each seta less than width of femur; tibiae mostly gray, only apices yellowish; basal tarsomeres yellow, apical 1–2 becoming slightly darker, mostly tannish yellow.

**Figures 53–57.**
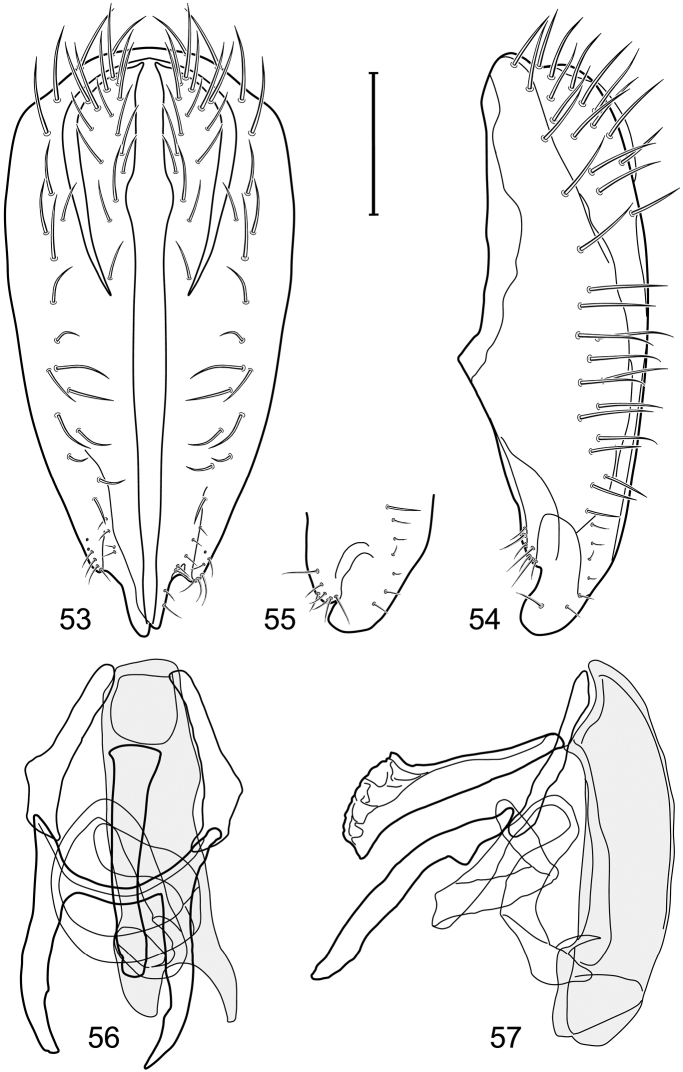
Illustration of *Polytrichophora flavella* sp. n. (male) (Peru. Madre de Dios: Rio Manu, Pakitza) **53** epandrium and cerci, posterior view **54** same, lateral view of left side **55** anterior portion of epandrium, lateral view of right side same, lateral view **56** internal structures of male terminalia (aedeagus [shaded], phallapodeme, gonite, hypandrium), ventral view **57** same, lateral view. Scale bar = 0.1 mm.

*Abdomen*: Tergites mostly blackish to brown gray, subshiny, with gray microtomentose areas laterally; dorsum of tergite 5 of male not darker than preceding tergites and usually with distinct, semicircular, grayish areas laterally; tergite 5 of male somewhat pointed posteriorly, lacking distinctive row of setulae apically. Male terminalia (Figs 53–57): Epandrium narrowly connected dorsally, in posterior view (Fig. 53) generally wedge-shaped, wider dorsally, tapered laterally, with a subapical, abrupt indentation, producing conspicuously more narrowly apices, setation more concentrated on dorsal half, setulae on ventral ¼ minute, in lateral view (Fig. 54) elongate, height about 3× width, with posterior margin arched and anterior margin with a shallow but distinct point, ventral portion with thumb-like apex and a subapical, anterior, shallow indentation; aedeagus complex with distinct basiphallus and elongate, contorted, distiphallus, basiphallus in lateral view (Fig. 57) sausage-like, distiphallus contorted, basiphallus in ventral view (Fig. 56) slightly asymmetrical, with a moderately deep, apical emargination; phallapodeme in lateral view (Fig. 57) with a distinct through short keel, generally elongate with keel at end toward hypandrium, in ventral view (Fig. 56) rod-like, slightly curved asymmetrically; gonite in lateral view (Fig. 57) elongate, rod-like, sinuous, in ventral view (Fig. 56) elongate, wider toward hypandrium, shallowly pointed subapically; hypandrium in lateral view (Fig. 57) elongate, very shallowly curved, pointed at each end, in ventral view (Fig. 56) H-shaped but with crossing bar much more toward connection with gonite.

#### Type material.

The holotype male is labeled “PERU. Madre de Dios: Manu, Rio Manu,250 m[,] Pakitza, 12[sic, 11]°7[six, 56.6]'S, 70[sic, 1]°58 [sic, 16.9]'W, 9-23 Sep 1988[,] Wayne N. Mathis/USNM ENT 00285978 [plastic bar code label]/HOLOTYPE ♂ *Polytrichophora flavella* Mathis & Zatwarnicki, USNM [red].” The holotype is double mounted (minuten pin in a block of plastic), is in excellent condition, and is deposited in the USNM. Thirty-seven paratypes (22♂, 15♀; USNM) bear the same label data as the holotype. Other paratypes are as follows: PERU. **Madre de Dios:** Río Manu, Erika (near Salvación; 12°50.7'S, 71°23.3'W; 550 m), 5-6 Sep 1988, A. Freidberg, W. N. Mathis (1♂, 2♀; USNM); Rio Manu, Pakitza (11°56.6'S, 71°16.9'W; 250 m), 9-23 Sep 1988, W. N. Mathis (23♂, 15♀; USNM); Rio Manu, Pakitza (5 km E; Aguajal; 11°58.2'S, 71°17'W; 250 m), 19 Sep 1988, W. N. Mathis (11♂, 8♀; USNM).

#### Type locality.

Peru. Madre de Dios: Rio Manu, Pakitza (11°56.6'S, 71°16.9'W; 250 m).

#### Other specimens examined.

Neotropical. PERU. **Loreto:** Iquitos (14 km W; 03°52.1'S, 73°28.2'W), 16 Feb 1984, W. N. Mathis (2♀; USNM).

#### Distribution

**Figure 58.**
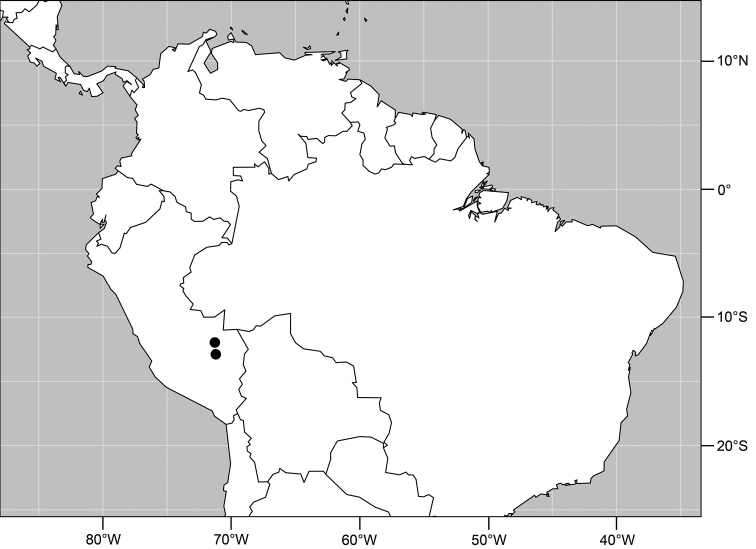
Distribution of *Polytrichophora flavella* sp. n.

(Fig. 58). Neotropical: Peru (Loreto, Madre de Dios).

#### Etymology.

The species epithet, *flavella*, is the Latin word for yellowish and refers to the yellow legs of this species.

#### Remarks.

This species, like *Polytrichophora agens*, has some asymmetry in structures of the male terminalia. Unlike *Polytrichophora agens*, however, this species has yellow legs, including most of the coxae, and the epandrium is rounded ventrally and has a subapical, anterior process (Figs 53–55).

### Polytrichophora marinoniorum sp. n.

urn:lsid:zoobank.org:act:9DE9B1C0-6FF5-4469-8E2A-88C4EDF44D3D

http://species-id.net/wiki/Polytrichophora_marinoniorum

Figs 59 63

#### Diagnosis.

This species is distinguished from congeners by the following combination of characters: Small to moderately small shore flies, body length 1.75–2.25 mm.

*Head*: Frons generally blackish brown, dull, moderately heavily microtomentose, mostly tan with some faint golden reflections on posterior 2/3, anterior 1/3 (from level of fronto-orbital setae anteriad), gray. Antenna mostly yellow to yellowish orange, dorsal portion of pedicel and basodorsal area of basal flagellomere with some blackish coloration; arista with 5 dorsal rays. Face at narrowest point about equal to combined length of pedicel and basal flagellomere; face densely microtomentose, microtomentum with subshiny to pearly luster, mostly white but with considerable gray to bluish gray coloration in antennal grooves and extended laterally onto parafacial; parafacial not markedly differing from midfacies in color, more golden dorsally, becoming whiter ventrally; parafacial with considerable ventral dilation; parafacial color not markedly different from that of middle of face, usually silvery white; parafacial 2–3 times wider ventrally than dorsally; gena relatively short, less than 1/4 eye height; gena-to-eye ratio 0.18–0.20.

*Thorax*: Mesonotum mostly subshiny, moderately densely microtomentose, dark brown, anterolateral areas bluish gray; pleural area blackish gray. Stout setae on apical half; anterior margin of wing lacking large, spine-like setae; costal vein ratio 0.70–0.77; M vein ratio 0.46–0.49. Forefemur lacking row of 9–10 short, stout setae along apical half of anteroventral surface; forefemur with posteroventral row of 7–10 short setulae, each equal to width of femur; tibiae mostly yellowish; basal tarsomeres yellow, apical 1–2 brown.

**Figures 59–62.**
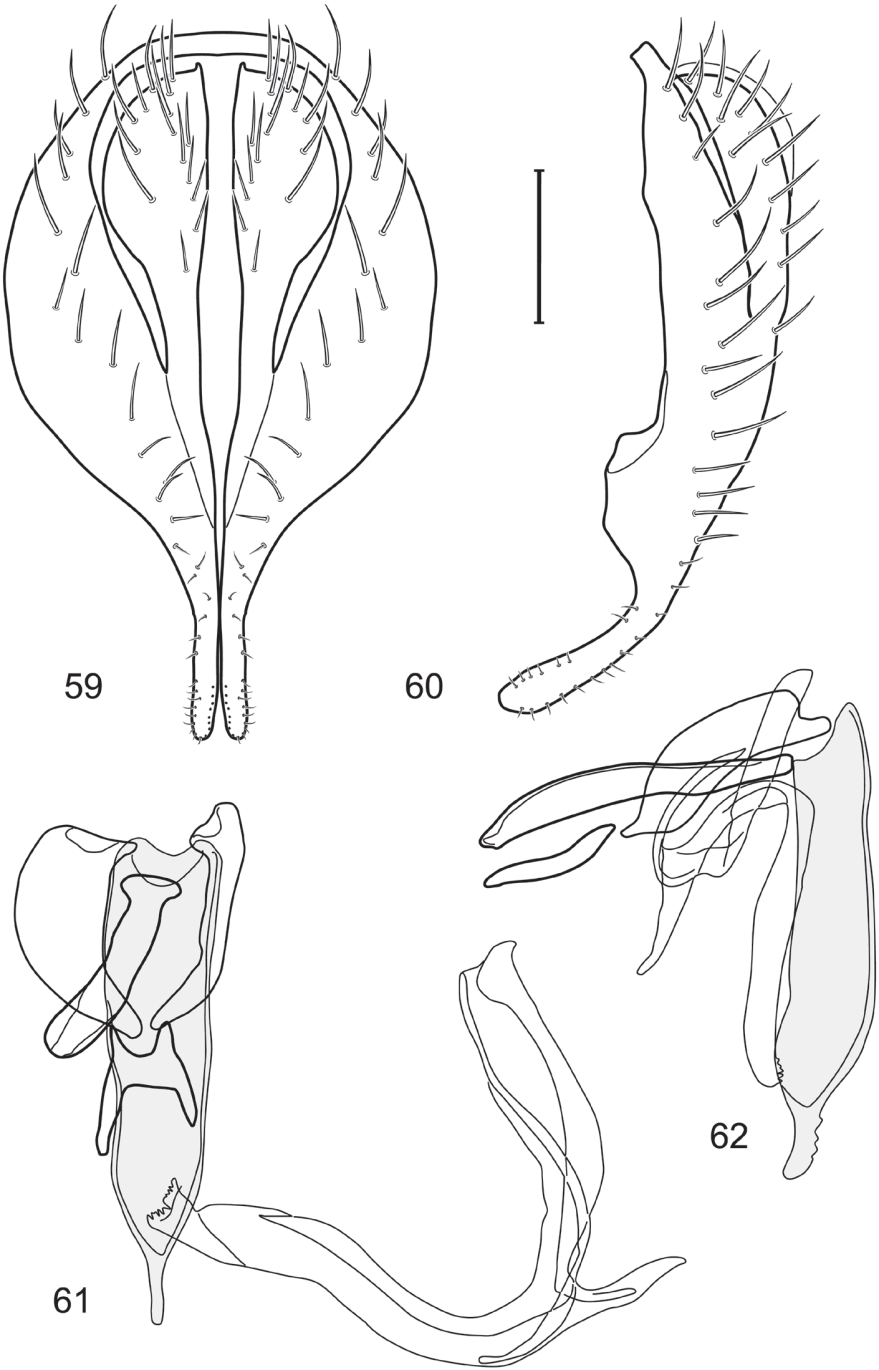
Illustration of *Polytrichophora marinoniorum* sp. n. (male) (Brazil. Paraná: Antonina) **59** epandrium and cerci, posterior view **60** same, lateral view **61** internal structures of male terminalia (aedeagus [shaded], phallapodeme, gonite, hypandrium), ventral view **62** same, lateral view. Scale bar = 0.1 mm.

*Abdomen*: Tergites dull to subshiny, gray to blackish brown; tergites 1–4 bicolored, mostly blackish brown dorsally, gray lateroventrally; tergites 3–4 of male subequal, 4th not unusually longer, tergite 3 not produced ventrolaterally; dorsum of tergite 5 of male not darker than preceding tergites and usually with distinct, semicircular, grayish areas laterally; tergite 5 of male in dorsal view blunt, truncate, bearing row of 6–10, distinctly larger setae along extreme posterior margin with posterodorsal orientation; tergite 5 of male also gray on posterior 1/2–1/3; sternites 3–4 of male with dense row of setulae along posterior margin; sternite 5 as 2 subtriangular sternites. Male terminalia (Figs 59–62): Epandrium connected dorsally above cerci, in posterior view (Fig. 59) broadly oval with ventral, tapered, narrow extensions, oval portion evenly setulose; extensions parallel sided, essentially abutting over most of length, narrowly separated apically, apex narrowly rounded, extensions minutely setulose; ventral third of epandrium in lateral view (Fig. 60) curved anteriorly, moderately robustly developed, slightly expanded apically, moderately narrowly spatulate, slightly curved anteroventrally, rounded apically; gonites asymmetrical, in lateral view (Fig. 62) narrowly rectangular, relatively short, each end with nipple, shallowly curved, in ventral view (Fig. 61) with flange on each side unequal, left side extended, right side not extended; aedeagus in lateral view (Fig. 62) complex, with distinct basiphallus that ends in a relatively short, wider, digitiform extension, and a twisted, contorted distiphallus; phallapodeme in lateral view (Fig. 62) elongate, rod-like, essentially lacking a keel, in ventral view (Fig. 61) curved laterally to left; hypandrium in ventral view (Fig. 61) relatively small, H-shaped, in lateral view (Fig. 61) elongate, tapered to point at each end.

#### Type material. 

The holotype male is labeled “**BRAZIL.** Paraná: Antonina(25°28.4'S, 48°40.9'W; mangal)[,] 4 Feb 2010[,] D. & W. N. Mathis/USNM ENT 00285969 [plastic bar code label]/HOLOTYPE ♂ *Polytrichophora marinoniorum* Mathis & Zatwarnicki, DZUP [red].” The holotype is double mounted (minuten pin in a black of plastic), is in excellent condition, and is deposited in DZUP. Fifteen paratypes (15♂; DZUP, USNM) bear the same locality label as the holotype with dates from 4 Feb-14 Nov. Other paratypes are as follows: BRAZIL. Paraná: Antonina (25°27.1'S, 48°41.1'W; beach; Ponta da Pita), 3–15 Feb 2010, D. and W. N. Mathis (11♂; DZUP, USNM); Matinhos (N; 25°46.4'S, 48°30.8'W; 1 m; beach/estuary), 30 Jan-25 Mar 2010, D. and W. N. Mathis (14♂, 6♀; DZUP, USNM)

#### Type locality.

Brazil. Paraná: Antonina (25°28.4'S, 48°40.9'W; mangal).

#### Other specimens examined.

Neotropical. BRAZIL. **Rio de Janeiro.** Ilha da Marambaia (23°3.6'S, 43°59.1'W), 4 Sep 2000, D. and W. N. Mathis (6♂, 9♀; DZUP, USNM). **Santa Catarina.** Barra Velha (26°38'S, 48°40.9'W; beach), 29 Apr 2010, D. and W. N. Mathis (5♂, 6♀; DZUP, USNM). **São Paulo.** Ubatuba, Praia do Estaleiro (23°20.5'S, 44°53'W; beach), 30 Mar 2010, D. and W. N. Mathis (1♂; USNM).

#### Distribution

**Figure 63.**
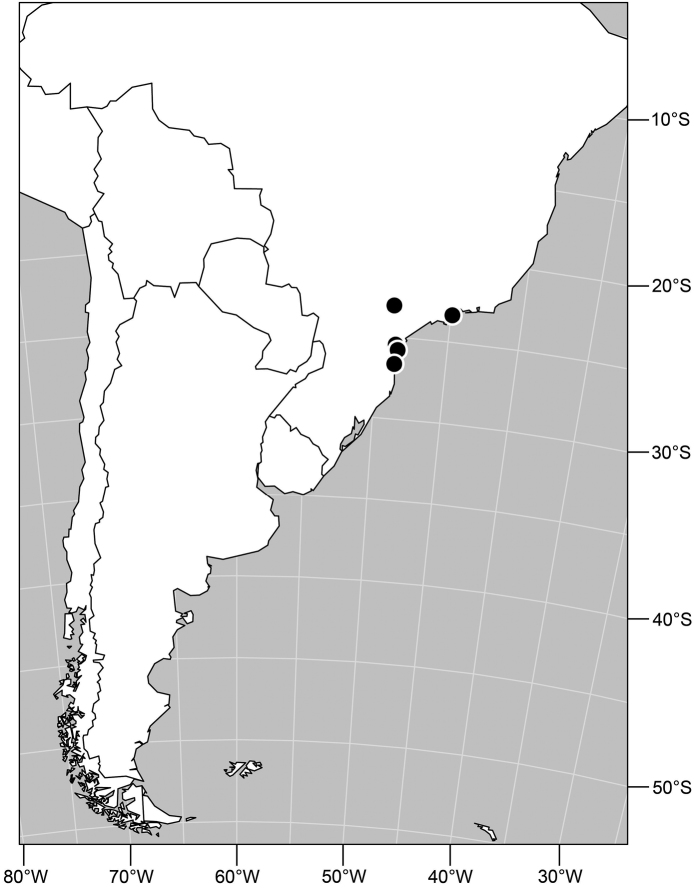
Distribution of *Polytrichophora marinoniorum* sp. n.

(Fig. 63). Neotropical: Brazil (Paraná, Rio de Janeiro, Santa Catarina, São Paulo).

#### Etymology.

The species epithet, *marinoniorum*, is a plural genitive patronym to recognize and honor Renato C. (1939–2011) and Neuza (nee Fonseca) Marinoni, who were gracious hosts and greatly fostered our field work in southern Brazil.

#### Remarks.

This species and *Polytrichophora conciliata* are very similar externally (forefemur with row of short, stout setulae along anteroventral margin). Characters of the male terminalia are likewise similar, but in detail, there are subtle differences in structures of the male terminalia (compare Fig. 59 with 48), such as the more rounded epandrium in posterior view and the slightly thinner ventral epandrial processes.

### Polytrichophora rostra sp. n.

urn:lsid:zoobank.org:act:0215113D-C01B-46AB-A4AE-60F7D5295276

http://species-id.net/wiki/Polytrichophora_rostra

Figs 64 68

#### Diagnosis.

This species is distinguished from congeners by the following combination of characters: Small to moderately small shore flies, body length 1.80–2.25 mm.

*Head*: Frons dull, moderately heavily microtomentose, partially2-toned, mostly grayish tan to grayish black with some faint golden reflections on posterior 3/4, anterior 1/4 becoming orange immediately along ptilinal suture. Antenna mostly yellow to yellowish orange, dorsal portion of pedicel and to a lesser degree basodorsal area of basal flagellomere with some blackish coloration; arista with 5 dorsal rays. Face at narrowest point about equal to combined length of pedicel and basal flagellomere; face densely microtomentose, microtomentum with subshiny to pearly luster, mostly yellowish white but with considerable gold coloration in antennal grooves and extended laterally onto parafacial; parafacial not markedly differing from midfacies in color, becoming silvery white ventrally and posteriorly; parafacial with slight ventral dilation; gena relatively short, less than 1/4 eye height; gena-to-eye ratio 0.18–0.19.

*Thorax*: Mesonotum mostly slightly dull to subshiny, moderately densely microtomentose, mostly brown to tan laterally; pleural area gray. Lacking stout setae on apical half; anterior margin of wing lacking large, spine-like setae; costal vein ratio 0.45–0.63; M vein ratio 0.55–0.58. Forefemur lacking row of 9-10 short, stout setae along apical half of anteroventral surface; forefemur with posteroventral row of 6–7 setulae, each subequal to width of femur; tibiae mostly yellowish at apices, otherwise gray medially; basal tarsomeres yellow, apical 1–2 brown.

**Figures 64–67.**
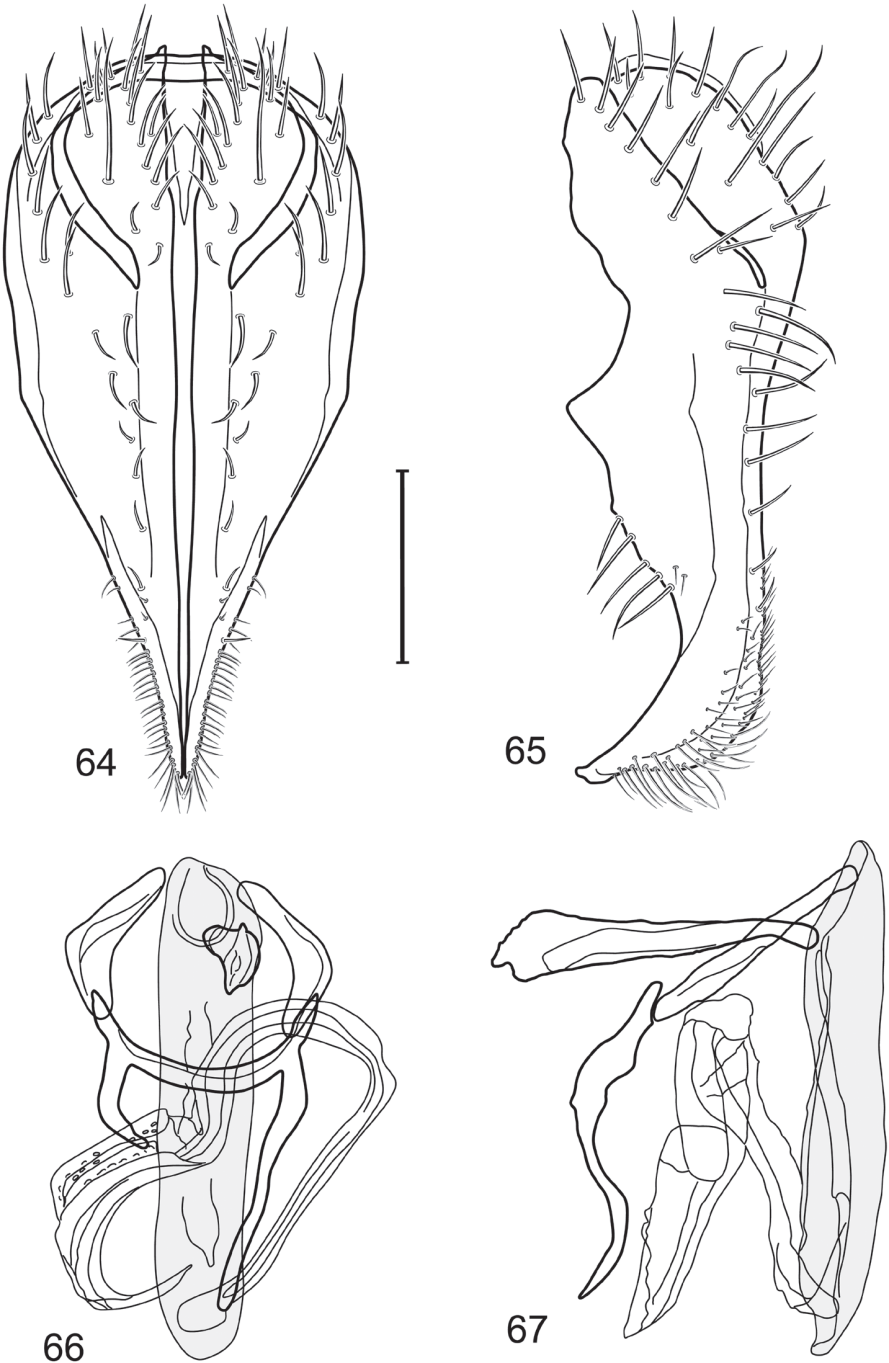
Illustration of *Polytrichophora rostra* sp. n. (male) (Ecuador. Napo: Pastaza) **64** epandrium and cerci, posterior view **65** same, lateral view **66** internal structures of male terminalia (aedeagus [shaded], phallapodeme, gonite, hypandrium), ventral view **67** same, lateral view. Scale bar = 0.1 mm.

*Abdomen*: Tergites subshiny to shiny medially, grayish black, becoming grayer laterally; tergite 5 of male somewhat pointed posteriorly, lacking distinctive row of setulae apically. Male terminalia (Figs 64–67): Epandrium in posterior view (Fig. 64) as an inverted tear drop, wide basally, at midheight, tapered ventrally to form a narrowly pointed ventral margin, connected dorsally around cerci, in lateral view (Fig. 65) with most of posterior margin mostly straight, angled anteriorly and either end, ventral portion forming a beak-like extension that tapers to a narrow point, anterior margin shallowly zigzagged, apical portion bearing numerous short setulae; cerci prominent, moderately well developed; aedeagus complex, in lateral view (Fig. 67) elongate, doubly folded back on itself, basiphallus elongate, narrow, pointed apically, distiphallus more narrowly developed than basiphallus, folded back twice with apical segment oriented in same direction as general plane of terminalia, with complicated smaller folds and lines, in ventral view (Fig. 66) with basiphallus tubular, elongate, distiphallus twisted and irregularly curved; phallapodeme in lateral view (Fig. 67) elongate, clavate, narrowed toward aedeagal base, keel only slightly extended, in ventral view (Fig. 66) appearing aforeshortened as small irregularly rounded structure with short knobs; gonite in lateral view (Fig. 67) rod-like, narrow, elongate, irregularly parallel sided, in ventral view (Fig. 66) angularly curved, like a boomerang; hypandrium in lateral view (Fig. 67) elongate, very narrow, sinuous, in ventral view (Fig. 66) irregularly H-shaped with bar shallowly curved and posterior arms subequal to each other, anterior arms, narrow, left arm considerably shorter than right arm, both narrowly developed.

#### Type material.

The holotype male is labeled “PERU. Madre de Dios: Manu, Rio Manu,250 m[,] Pakitza, 12[sic, 11]°7[six, 56.6]'S, 70[sic, 1]°58 [sic, 16.9]'W, 9-23 Sep 1988[,] Wayne N. Mathis/USNM ENT 00285963 [plastic bar code label]/HOLOTYPE ♂ *Polytrichophora rostra* Mathis & Zatwarnicki, USNM [red].” The holotype is double mounted (minuten pin in a block of plastic), is in good condition (abdomen removed and dissected, midlegs missing), and is deposited in the USNM. One paratype (♂; USNM) bears the same label data as the holotype. Other paratypes are as follows: PERU. **Cuzco:** Paucartambo, Atalaya (Río Alto Madre de Dios; 12°53.1'S, 71°21.6'W; 600 m), 4 Sep 1988, W. N. Mathis (1♂; USNM).

#### Type locality.

Peru. Madre de Dios: Rio Manu, Pakitza (11°56.6'S, 71°16.9'W; 250 m).

#### Other specimens examined.

Neotropical. ECUADOR. **Napo:** Pastaza (01°38'S, 77°0.1'W), J. R. Levi-Castillo (1♂; USNM).

#### Distribution

**Figure 68.**
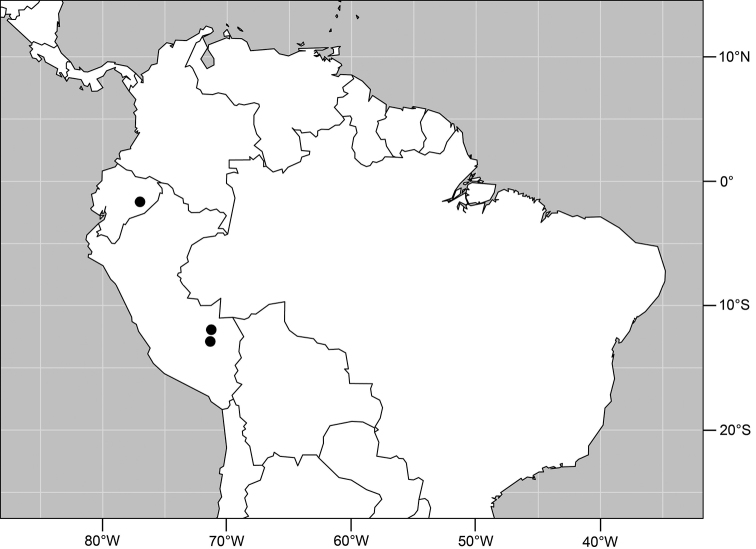
Distribution of *Polytrichophora rostra* sp. n.

(Fig. 68). Neotropical: Ecuador (Napo), Peru (Cuzco, Madre de Dios).

#### Etymology.

The species epithet, *rostra*, is the Latin word for beak and refers to the shape of the beak-like epandrium as viewed in lateral view.

#### Remarks.

Like *Polytrichophora setulosa*, this species has asymmetry in the anterior hypandrial arms, which is probably a synapomorphy that indicates a close relationship with that species. This species is distinguished by the beak-like apical process of the epandrium, best seen in lateral view (Fig. 65), which is unmistakable and is the primary character that distinguishes this species from congeners within the *conciliata* group.

### Polytrichophora setulosa

(Cresson)

http://species-id.net/wiki/Polytrichophora_setulosa

Figs 69 73

Discocerina setulosa Cresson 1918: 58.
Polytrichophora setulosa . Cresson 1946: 143 [generic combination, review of Neotropical fauna]. Wirth 1968: 9 [Neotropical catalog]. Mathis and Zatwarnicki 1995: 186 [world catalog].

#### Diagnosis.

This species is distinguished from congeners by the following combination of characters: Small to moderately small shore flies, body length 1.70–2.60 mm.

*Head*: Frons dull, heavily microtomentose, 2-toned, mostly tan to brown with some faint golden reflections on posterior 2/3, especially medially, anterior 1/3 (from level of fronto-orbital setae anteriad), gray. Antenna mostly yellow to yellowish orange, anterior portion of pedicel and basodorsal area of basal flagellomere with some blackish coloration; arista with 5 dorsal rays. Face at narrowest point about equal to combined length of pedicel and basal flagellomere; face and clypeus densely microtomentose, especially ventrally, less so dorsally, microtomentum with shiny to pearly luster, mostly white but with considerable gold coloration in antennal grooves and extended laterally onto parafacial; parafacial not markedly differing from midfacies in color, more golden dorsally, becoming whiter ventrally; parafacial with moderate ventral dilation, 2–3 times wider ventrally than dorsally; gena moderately short, about equal to height of basal flagellomere; gena-to-eye ratio 0.19–0.21.

*Thorax*: Mesonotum mostly dull, moderately densely microtomentose, partially subshiny, concolorous with posterior 2/3 of frons; pleural area blackish gray. Stout setae on apical half; anterior margin of wing lacking large, spine-like setae; costal vein ratio 0.60–0.76; M vein ratio 0.48–0.58. Forefemur lacking row of 9–10 short, stout setae along apical half of anteroventral surface; forefemur with posteroventral row of 7–10 short setulae, each equal to width of femur; tibiae mostly yellowish; basal tarsomeres yellow, apical 1–2 brown.

**Figures 69–72.**
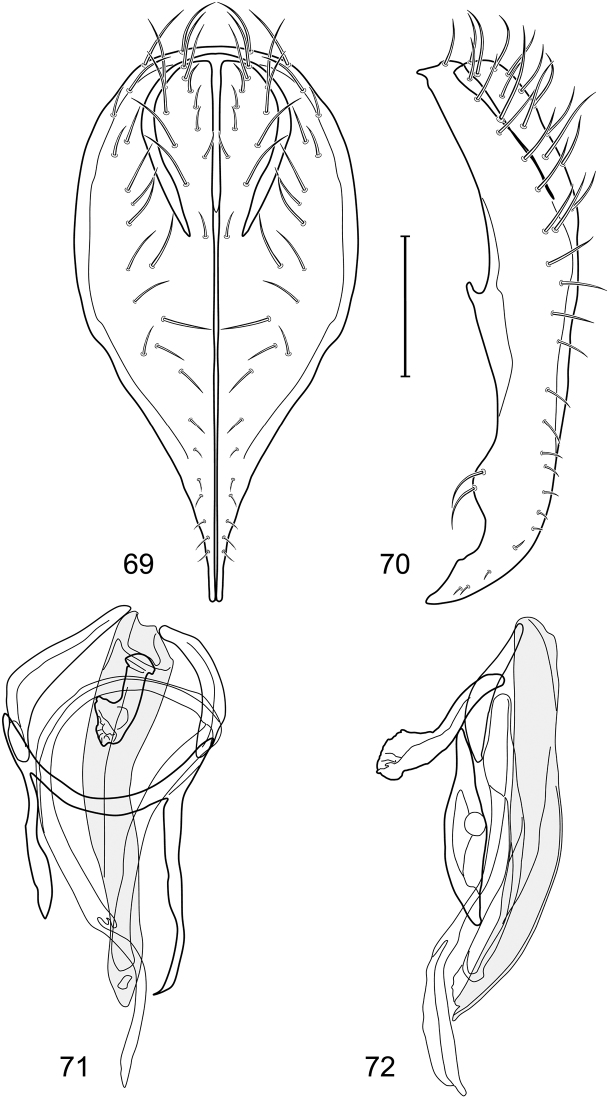
Illustration of *Polytrichophora setulosa* (Cresson) (male) (Jamaica. St. Elzbeth: Elim) **69** epandrium and cerci, posterior view **70** same, lateral view **71** internal structures of male terminalia (aedeagus [shaded], phallapodeme, gonite, hypandrium), ventral view **72** same, lateral view. Scale bar = 0.1 mm.

*Abdomen*: Tergites dorsally 2-toned, laterally lightly gray, becoming distinctly darker and subshiny medially; tergite 5 of male mostly gray medially, with whitish gray areas laterally, broadly pointed posteriorly. Male terminalia (Figs 69–72): Epandrium in posterior view (Fig. 69) mostly oval with relatively elongate, narrow, tapered, pointed ventral extensions, ventral extension in lateral view (Fig. 70) robustly developed, curved and tapered apically to acute point, bearing secondary point at midlength of extension that bears setulae; aedeagus complex, narrow, elongate, with distinct basiphallus and distiphallus, distiphallus with twisted, partially coiled, very elongate bands (Figs 71–72) very shallowly arched, almost straight and parallel sided, apex bluntly rounded; phallapodeme in lateral view (Fig. 72) relatively short, clavate with larger end toward hypandrium, sinuous, in ventral view (Fig. 71) asymmetrical, especially shallow keel at more clavate end; gonite in lateral view (Fig. 72) elongate, shallowly sinuous, slightly larger toward hypandrium, in ventral view (Fig. 71) asymmetrical with gonite on left side larger than right one, left gonite more angulate, right gonite more shallowly and evenly curved; hypandrium in lateral view (Fig. 72) moderately elongate, narrowly elliptical, in ventral view H-shaped but with anterior arms of differing lengths and much shorter posterior arms.

#### Type material.

The holotype male is labeled “Filadelfia[,] Costa Rica/muddy beach R[io].Tempisque/PPCalvert 18. 1 1910 [18 Jan 1910][date handwritten]/♂/TYPE 6133/690/Holo-TYPE Discocerina SETULOSA E. T. Cresson Jr [magenta; species name handwritten].” The holotype is double mounted (minuten pin in a rectangular card), is in good condition (the abdomen and hind legs have been removed, the abdomen dissected, and the parts are in an attached microvial), and is deposited in the ANSP (6133).

#### Type locality.

Costa Rica. Guanacaste: Filadelfia (Río Tempisque; 10°26.7'N, 85°32.9'W).

#### Other specimens examined.

Neotropical. BELIZE. **Stann Creek:** Twin Cays (Aanderaa Flats, dock area, east shore of East Island, mud flat near Lair Channel, south end of West Island, West Bay; 16°49.9'N, 88°06.1'W), 17-21 Mar 1988, W. N. Mathis (1♂, 1♀; USNM); Mullins River (17 km N Dangriga; 17°06.2'N, 88°17.8'W), 29 Mar 1988, W. N. Mathis (1♂; USNM); Placencia Lagoon, Rum Point (16°32.8'N, 88°22.1'W), 4-5 Nov 1987, D. and W. N. Mathis (4♀; USNM).

BOLIVIA. **La Paz:** Guanay (3 km E; 15°30.2'S, 67°52.3'W; 500 m), 14 Mar 2001, W. N. Mathis (1♂; USNM).

BRAZIL. **Amazonas.** Igarapé Cabeça Branca (ca. 40 km N Manaus; 02°35.1'S, 60°01.9'W; 65 m), 8 May 2010, D. and W. N. Mathis (1♂; USNM); Manaus, INPA (03°05.9'S, 59°59.1'W; 60 m), 4 May 2010, D. and W. N. Mathis (1♀; INPA. **Paraná.** Antonina (25°28'S, 48°41.3'W; 13 m), 3 Feb 2010, D. and W. N. Mathis (1♀; DZUP, USNM); Antonina (25°28.4'S, 48°40.9'W; beach/mangal), 3 Feb 2010, D. and W. N. Mathis (1♂; DZUP, USNM); Castro (Parque Lacustre; 24°47.4'S, 50°0.3'W; 990 m), 24-31 Dec 2009, D. and W. N. Mathis (2♂, 1♀; USNM); Castro (8 km N; 24°45.3'S, 49°58.9'W; 1010 m), 1 Jan 2010, D. and W. N. Mathis (1♀; DZUP, USNM); Curitiba, Universidade Federal do Paraná, Reserva Biológica (25°26.9'S, 49°14'W; 915 m), 1 Jan-24 Mar 2010, D. and W. N. Mathis (1♂, 2♀; DZUP, USNM); Matinhos (N.; 25°46.4'S, 48°30.8'W; 1 m; beach/estuary), 30 Jan25 Mar 2010, D. and W. N. Mathis (6♂, 2♀; DZUP, USNM); Parque Igauçu (25°33.4'S, 49°13.6'W; 880 m), 19 Jan-12 Apr 2010, D. and W. N. Mathis (11♂, 5♀; DZUP, USNM). **Santa Catarina.** Barra Velha (26°38'S, 48°40.9'W; beach), 29 Apr 2010, D. and W. N. Mathis (2♂; DZUP, USNM); Nova Teutônia (27°11'S, 52°23'W; 3–500 m), Oct 1970, F. Plaumann (1♀; MZUSP). **São Paulo.** Ubatuba, Praia do Estaleiro (23°20.5'S, 44°53'W; beach), 30 Mar 2010, D. and W. N. Mathis (3♂; DZUP, USNM); Ubatuba, Praia Puruba (23°21'S, 44°55.6'W; beach), 29–30 Mar 2010, D. and W. N. Mathis (7♂; USNM).

COSTA RICA. **Cartago:** La Suiza (9°51.5'N, 83°37.5'W), 28 Jun 2001, W. N. Mathis (25♂, 5♀; USNM). **Puntarenas:** Rincón (5 km S; 8°42.1'N, 83°30.8'W; 95 m), 10–11 Aug 2001, D. and W.N. Mathis (2♀; USNM).

DOMINICAN REPUBLIC. **Barahona:** Cabral (canals E of Cabral; 18°15.2'N, 71°13.4'W), 16 May 1995, W. N. Mathis (2♂, 1♀; USNM). **Independencia:** Duvergé (2 km S; 18°22'N, 71°31.4'W), 24 Mar 1999, W. N. Mathis (9♂; USNM).**La Vega:** Constanza (ca. 16 km SE; 18°50.6'N, 70°40.7'W; 1580 m), 15 May 1998, D. and W. N. Mathis (1♂; USNM); El Rio (9.5 km E; 19°0.9'N, 70°33.5'W; 980 m), 6 May 1995, W. N. Mathis (5♂, 2♀; USNM); Jarabacoa (1–2 km S; 19°06.9'N, 70°37'W; 520 m), 8–21 May 1995, W. N. Mathis (14♂, 8♀; USNM); Rio Camu (3.5 km NW La Vega; 19°13.7'N, 70°35.2'W; 100 m), 10 May 1995, W. N. Mathis (1♂, 2♀; USNM). **Puerto Plata:** Rio Camu (14 km E Puerto Plata; 19°41.9'N, 70°37.5'W), 23 May 1998, D. and W. N. Mathis (1♂; USNM).

GUYANA. Dubulay Ranch (small creek; 5°40.9'N, 57°51.5'W), 10 Apr 1994, W. N. Mathis (♂, ♀; USNM); Dubulay Ranch, Berbice River (5°40.9'N, 57°51.5'W), 9–11 Apr 1994, W. N. Mathis (♂, ♀; USNM); Karanambo (3°45.1'N, 59°18.6'W), 31 Mar 1994, W. N. Mathis (♂, ♀; USNM); Karanambo (Rupununi River; 3°45.1'N, 59°18.6'W; 85m), 1–3 Dec 2010, W. N. Mathis (1♂, 1♀; USNM); Karanambo, Rupununi River (ox bow; 3°45.1'N, 59°18.6'W), 2 Apr 1994, W. N. Mathis (♂, ♀; USNM); Karanambo, Rupununi River (ox bow; 3°45'N, 59°17.5'W; 85m), 2 Dec 2010, W. N. Mathis (2♂; USNM); Kumu River and Falls (25 km SE Lethem in Kanuku Mountains; 3°15.9'N, 59°43.6'W), 4–30 Apr 1994, 1995, W. N. Mathis (♂, ♀; USNM); Wiruni River (5°46.6'N, 58°01'W), 11 Apr 1994, W. N. Mathis (♂, ♀; USNM).

HONDURAS. **Cortés:** San Pedro Sula (8 km S; 15°25.7'N, 88°01.4'W), 25–26 Sep 1995, D. and W. N. Mathis (3♂, 1♀; USNM).

JAMAICA. **Clarendon:** Grantham (18°09.3'N, 77°23.8'W; 340 m), 16 Apr 2000, W. N. Mathis (3♂, 1♀; USNM); Rest (3 km N; 17°54.3'N, 77°21.4'W), 15 Apr 2000, W. N. Mathis (1♂; USNM). **Portland:** Berridale (18°06.5'N, 76°20'W), Rio Grande River, 25 Apr 2000, W. N. Mathis (1♂, 1♀; USNM); Hollywell (18°05.5'N, 76°43.6'W; 1170 m), 27 Apr 2000, W. N. Mathis (1♀; USNM); Reach (4 km N; 18°03.6'N, 76°20.4'W), 15 May 1996, D. and W. N. Mathis, H. B. Williams (3♂; USNM). **St. Andrew:** Clydsdale (18°04.9'N, 76°40.2'W; 1030 m), 29 Apr 2000, W. N. Mathis (2♀; USNM); Mavis Bank (1.7 km E; 18°02.4'N, 77°39.5'W; 575 m), Yallahs River, 21–22 Apr-1 May 2000, W. N. Mathis (3♀; USNM); Mavis Bank (4.3 km SE; 18°01.4'N, 76°38.1'W; 480 m); Yallahs River, 22–23 Apr 2000, W. N. Mathis (5♂, 3♀; USNM); Silver Hill Gap (18°05.3'N, 76°43'W; 940 m), 29 Apr 2000, W. N. Mathis (2♂, 1♀; USNM). **St. Elizabeth:** Elim (18°07.1'N, 77°40.5'W), 10 Apr 2000, W. N. Mathis (18♂, 3♀; USNM); Maggotty Falls (18°08.2'N, 77°45.1'W), 18 Apr 2000, W. N. Mathis (1♂; USNM); Wally Wash Great Pond (17°57.9'N, 77°48.6'W), 19 Apr 2000, W. N. Mathis (1♂; USNM); Ys Falls (18°09.3'N, 77°49.5'W), 17–18 Apr 2000, W. N. Mathis (1♀; USNM). **St. Mary:** Annotto Bay (18°16.2'N, 76°46.2'W; marsh), 25 Feb 1969, W. W. Wirth (4♂, 5♀; USNM). **St. Thomas:** Bath River, Bath (17°56.8'N, 76°21.6'W), 16 May 1996, D. and W. N. Mathis, H. B. Williams (1♀; USNM). **Westmoreland:** Savanna-La-Mar (18°13'N, 78°08'W), 13 Mar 1970, W. W. Wirth (1♀; USNM).

MEXICO. **Chiapas:** Finca Prusia (33 km S Jaltenango; 15°49'N, 92°42'W), 10–12 May 1985, W. N. Mathis (7♂, 21♀; USNM); Jaltenango (21 km S; 15°54'N, 92°40'W), 16 May 1985, W. N. Mathis (2♂, 2♀; USNM). **Tabasco:** Teapa (8 km SW; 17°31'N, 92°59'W), 6 May 1985, W. N. Mathis (2♂, 1♀; USNM). **Veracruz-Llave:** Fortin de las Flores (18°54'N, 97°W), 2 May 1985, W. N. Mathis (2♂, 1♀; USNM).

PUERTO RICO. Adjuntas (18°09.8'N, 66°43.2'W), 22 Sep 1995, D. and W. N. Mathis (5♂, 1♀; USNM); Jayuya (2 km E; Rio Saliente; 18°12.8'N, 66°33.9'W), 22 Sep 1995, D. and W. N. Mathis (2♂; USNM); Maricao (4 km WNW; 18°10.7'N, 66°59.6'W), 21 Sep 1995, D. and W. N. Mathis (12♂, 3♀; USNM); Playa de Guayanilla (18°0.4'N, 66°46.1'W), 19 Sep 1995, D. and W. N. Mathis (1♂; USNM).

ST. LUCIA. Castries (5 km S; 13°59'N, 60°00'W), 16 Jun 1991, D. and W. N. Mathis (2♂, 4♀; USNM); Laborie (5 km W; 13°49'N, 60°54'W), 15 Jun 1991, D. and W. N. Mathis (2♀; USNM).

#### Distribution

**Figure 73.**
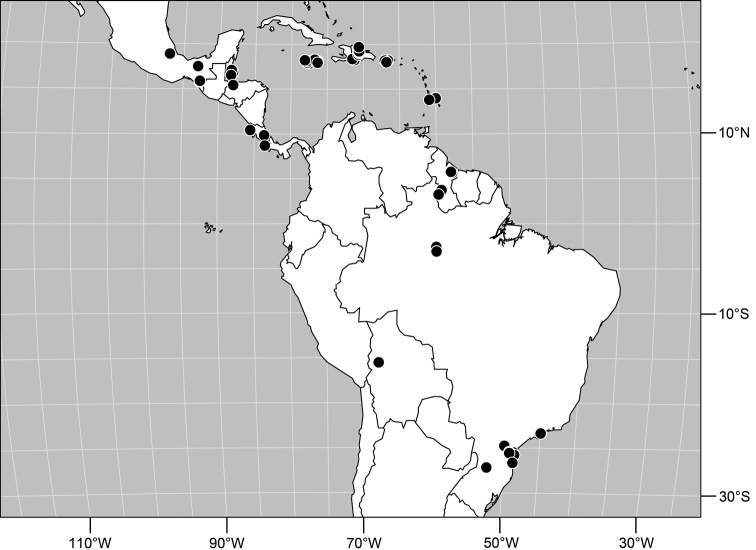
Distribution of *Polytrichophora setulosa* (Cresson).

(Fig. 73). Neotropical: Belize, Bolivia (La Paz), Brazil (Amazonas, Paraná, Santa Catarina, São Paulo), Costa Rica (Cartago, Puntarenas), Guyana, Honduras, Mexico (Chiapas, Tabasco, Veracruz-Llave), West Indies (Dominican Republic, Jamaica, Puerto Rico, St. Lucia).

#### Remarks.

Among congeners of the *conciliata* group, this species is very similar to *Polytrichophora rostra* as evidenced by both species having a crescent-shaped ventral epandrial process and pronounced asymmetry of the anterior hypandrial arms. This species is distinguished from *Polytrichophora rostra*, however, in the lateral view by the shape of the epandrium (Fig. 70), which has a secondary prong and has more gradual curvature of the ventral epandrial process, not abrupt as in *Polytrichophora rostra* (Fig. 65).

### Polytrichophora sinuosa sp. n.

urn:lsid:zoobank.org:act:A4D253E3-FFF6-4662-B9B1-A02143679F10

http://species-id.net/wiki/Polytrichophora_sinuosa

Figs 74 78

#### Diagnosis.

This species is distinguished from congeners by the following combination of characters: Small to moderately small shore flies, body length 1.65–2.10 mm.

*Head*: Frons dull, heavily microtomentose, 2-toned, mostly tan with some faint golden reflections on posterior 2/3, anterior 1/3 (from level of fronto-orbital setae anteriad), gray. Antenna mostly yellow to yellowish orange, anterior portion of pedicel and basodorsal area of basal flagellomere with some blackish coloration; arista with 5 dorsal rays. Face at narrowest point about equal to combined length of pedicel and basal flagellomere; face densely microtomentose, microtomentum with shiny to pearly luster, mostly yellowish to golden white but with considerable gold coloration in antennal grooves and extended laterally onto parafacial; parafacial not markedly differing from midfacies in color, more golden dorsally, becoming whiter ventrally; parafacial with slight to considerable ventral dilation; parafacial color not markedly different from that of middle of face, usually silvery white; parafacial 2–3 times wider ventrally than dorsally; gena moderately short, less than 1/4 eye height, subequal to height of basal flagellomere; gena-to-eye ratio 0.18–0.20.

*Thorax*: Mesonotum mostly dull, densely microtomentose, concolorous with posterior 2/3 of frons; pleural area blackish gray. Stout setae on apical half; anterior margin of wing lacking large, spine-like setae; costal vein ratio 0.65–0.70; M vein ratio 0.53–0.57. Forefemur lacking row of 9–10 short, stout setae along apical half of anteroventral surface; forefemur with posteroventral row of 7–10 short setulae, each equal to width of femur; tibiae mostly yellowish, mid- and hindtibia whitish to grayish over middle portion; basal tarsomeres yellow, apical 1–2 brown.

**Figures 74–77.**
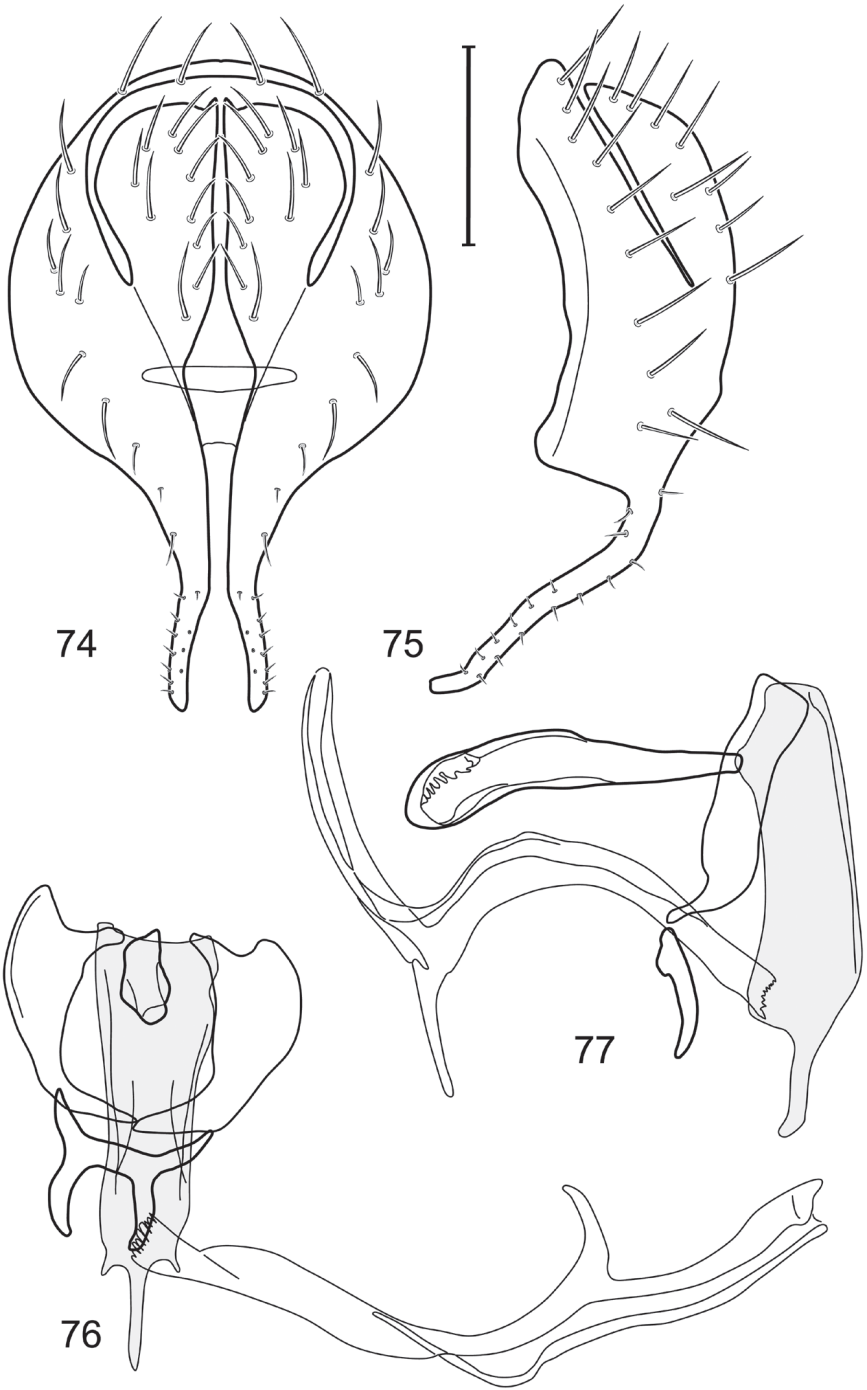
Illustration of *Polytrichophora sinuosa* sp. n. (male) (Trinidad. St. Andrew: Lower Manzanilla) **74** epandrium and cerci, posterior view **75** same, lateral view **76** internal structures of male terminalia (aedeagus [shaded], phallapodeme, gonite, hypandrium), ventral view **77** same, lateral view. Scale bar = 0.1 mm.

*Abdomen*: Tergites mostly broadly blackish brown, subshiny to shiny dorsomedially; tergite 5 of male broadly pointed posteriorly. Male terminalia (Figs 74–77): Epandrium connected dorsally above cerci (Fig. 75), in posterior view (Fig. 75) broadly oval with ventral, tapered extensions, oval portion evenly setulose; extensions parallel sided, separated apically with wide gap, apex narrowly rounded, extensions minutely setulose; ventral third of epandrium in lateral view (Fig. 75) shallowly sinuous, very narrow and elongate, curved anteroventrally, pointed apically; gonites almost symmetrical, in lateral view rod-like but relatively short, end toward hypandrium tapered and curved, opposite end truncate, in ventral view (Fig. 74) with flange on each side almost equal; aedeagus in lateral view (Fig. 77) complex, with distinct basiphallus that ends in an elongate digitiform extension, and a twisted distiphallus; phallapodeme in lateral view (Fig. 77) elongate, clavate, keel barely evident, in ventral view (Fig. 76) short, curved laterally to right; hypandrium in ventral view (Fig. 76) H-shaped, relatively small, in lateral view (Fig. 77) moderately elongate, tapered to point anteriorly.

#### Type material.

The holotype male is labeled “**TRINIDAD.** St. Andr[ew].: Low[er]. Manzanilla (12 km S, 10°24'N, 61°02'W)[,] bridgeNarivaRiv[er],20–27Jun1993, WNMathis/USNM ENT 00285967 [plastic bar code label]/HOLOTYPE ♂ *Polytrichophora sinuosa* Mathis & Zatwarnicki, USNM [red].” The holotype is double mounted (minute in a block of plastic), is in excellent condition, and is deposited in the USNM. One male paratype (USNM) bears the same label data as the holotype. A female paratype (USNM) is from TRINIDAD and TOBAGO. Trinidad. St. Andrew: Lower Manzanilla (16 km S; 10°22'N, 61°01'W), 20 Jun 1993, W. N. Mathis.

#### Type locality.

Trinidad and Tobago. Trinidad. St. Andrew: Lower Manzanilla (12 km S; 10°24'N, 61°02'W).

#### Other specimens examined.

Neotropical. TRINIDAD AND TOBAGO. Trinidad. **St. Andrew:** Lower Manzanilla (12 km S; 10°24'N, 61°02'W), bridge over Nariva River, 20–27 Jun 1993, W. N. Mathis (2♂, ♀; USNM). Tobago. **St. John:** Hermitage River and beach (11°18.9'N, 60°34.5'W), 22 Apr 1994, D. and W. N. Mathis (2♀; USNM).

#### Distribution

**Figure 78.**
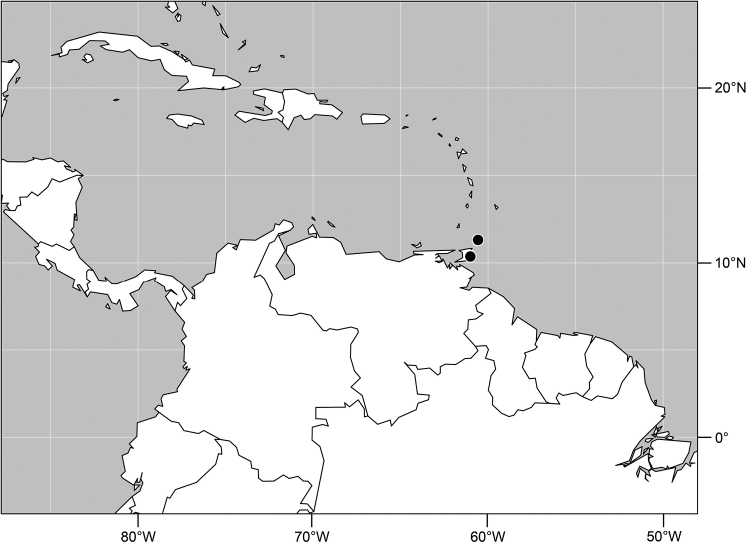
Distribution of *Polytrichophora sinuosa* sp. n.

(Fig. 78). Neotropical: Trinidad and Tobago.

#### Etymology.

The species epithet, *sinuosa*, is of Latin derivation and means winding in reference to the sinuous ventral extensions of the epandrium of this species.

#### Remarks.

This is a species of the *conciliata* group and is distinguished from other congeners of that group by the following combination of characters: Parafacial and gena not greatly dilated and high; forefemur lacking row of setulae along anteroventral surface, and setae along posteroventral surface are not elongate; tibiae yellowish (often with dorsal surface silvery white), and ventral epandrial extensions elongate, thinly developed, and sinuous in lateral view (Fig. 75).

##### The *desmata* Group

**Species included:** *Polytrichophora desmata* (Williston), *Polytrichophora pulchra* (Cresson).

**Discussion.** This species group is distinguished from the other groups by the following combination of characters: Parafacial color contrasted sharply with the much darker midfacies; parafacial with little or no ventral dilation; and abdomen mostly shiny, brownish black to black.

### Polytrichophora desmata

(Williston)

http://species-id.net/wiki/Polytrichophora_desmata

Figs 79 83

Psilopa desmata Williston 1896: 395. Wirth 1968a: 9 [Neotropical catalog; listed as an unrecognized species].
Polytrichophora desmata . Mathis and Edmiston 1991: 826 [generic combination]. Mathis and Zatwarnicki 1995: 185 [world catalog]. Zatwarnicki and Mathis 2001: 46 [figs of ♂ terminalia].
Polytrichophora boriqueni Cresson 1930: 77; 1946: 143 [review of Neotropical fauna]. Wirth 1968: 8 [Neotropical catalog]. Mathis and Edmiston 1991: 826 [synonymy].

#### Diagnosis.

This species is distinguished from congeners by the following combination of characters: Small to moderately small shore flies, body length 1.60–2.05 mm.

*Head*: Frons mostly black with moderate dusting of gray to whitish gray microtomentum; ocellar triangle slightly lighter in color; fronto-orbits creamy white, narrow. Antenna mostly yellow; basal flagellomere darkened apicodorsally; arista bearing 5 dorsal rays. Face, especially ventrad of antennal grooves, narrow, width less than combined length of scape and pedicel; facial color mostly dark but with investment of gray microtomentum medially; parafacial creamy to yellowish white, contrasted with the much darker midfacies; parafacials with little or no dilation ventrally; gena very short, height much less than height of basal flagellomere; gena-to-eye ratio 0.06–0.08.

*Thorax*: Mesonotum subshiny to shiny, blackish brown; postpronotum bluish gray; pleural areas blackish brown with sparse to moderate dusting of bluish gray to brownish gray microtomentum. Lacking stout setae along anterior margin of wing; costal vein ratio 0.84–0.86; M vein ratio 0.50–0.53. Forefemur with distinct row of 5–7 moderately long, slightly larger setae along posteroventral surface, lacking stout setae along apical half of anteroventral surface; femora blackish brown; tibiae darkened dorsally, becoming yellowish ventrally and at apices; basal tarsomeres yellow, apical 1–2 brown.

**Figures 79–82.**
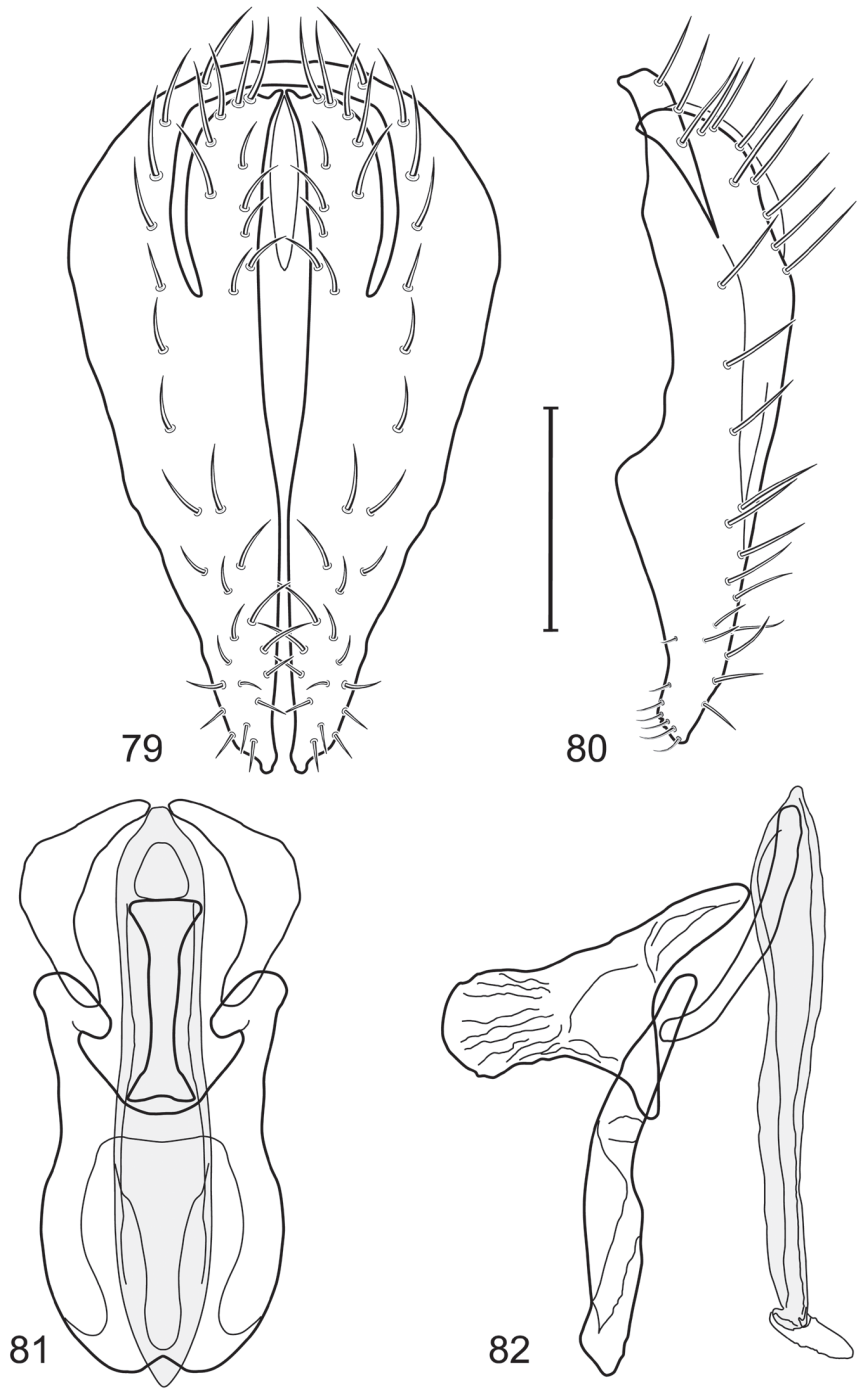
Illustration of *Polytrichophora desmata* (Williston) (male) (Dominica. Woodford Hill) **79** epandrium and cerci, posterior view **80** same, lateral view **81** internal structures of male terminalia (aedeagus [shaded], phallapodeme, gonite, hypandrium), ventral view **82** same, lateral view. Scale bar = 0.1 mm.

*Abdomen*: Tergites 1–4 subshiny, thinly microtomentose, blackish brown; tergite 5 of male distinctly more sparsely microtomentose and shinier than tergites 1–4, black. Male terminalia (Figs 79–82): Epandrium in posterior view (Fig. 79) generally as a rounded, elongate triangle, more or less uniformly setulose, dorsal margin broadly rounded, lateral margins tapered medially, both ventral extensions pointed, narrowly connected dorsally, in lateral view (Fig. 80) with ventral apex acutely pointed, cercus comparatively small but conspicuous; aedeagus narrowly tubular, in lateral view (Fig. 80) gradually and slightly tapered to narrowed apex, in posterior view (Fig. 79) moderately narrowly elongate with lateral margins nearly parallel sided; phallapodeme in lateral view (Fig. 82) roughly triangular with extended keel broadly rounded apically, in ventral view (Fig. 81) clavate with basal margin bluntly rounded; gonite in lateral view (Fig. 82) narrowly elongate, shallowly curved, in ventral view (Fig. 81) angularly arched, broader medially, narrowed at each apices; hypandrium in lateral view (Fig. 82) elongate, with lateral, irregular extension, in ventral view (Fig. 81) more or less H-shaped, with anterior portion having a membranous medial portion, posterolateral projections angularly hooked with hook robustly developed.

#### Type material.

The holotype male of *Psilopa desmata* is labeled “Type [round label with a red border]/Windward side St. Vincent, W.I. H. H. Smith./W.Indies. 1907–66./Psilopa desmata Will [handwritten, two red submarginal borders].” The holotype is double mounted (pin in a rectangular piece of cardboard), is in poor condition (head missing; mesonotum cracked anteriorly), and is deposited in the BMNH.

The holotype male of *Polytrichophora boriqueni* is labeled “Adjuntas, P[uerto].R[ico]. June 26, 1915/1105/TYPE Polytrichophora BORIQUENI E. T.Cresson,Jr. [magenta; species and generic names handwritten].” The holotype is double mounted (glued to a paper point), is in fair condition, and is deposited in the ANSP (no type number; Cresson stated that this specimen was to be deposited in the collection of the New York Academy of Sciences). Paratype is as follows: PUERTO RICO. Mayaguez (18°12.1'N, 67°08.7'W), 15–16 1914 (1♂; ANSP).

**Type locality.** West Indies. St. Vincent, windward side.

**Other specimens examined.** Neotropical. CUBA. **Cienfuegos:** Soledad, Jardin Botánico (22°7.5'N, 80°19.2'W), 13 Dec 1994, W. N. Mathis (2♂, 2♀; USNM); Topes de Collantes (5 km WNW; 21°56.5'N, 80°2.3'W; 600 m), 11 Dec 1994, W. N. Mathis (7♂, 3♀; USNM). **Pinar del Rio:** Soroa (22°47.7'N, 83°W), 4-6 Dec 1994, W. N. Mathis (1♂; USNM). **Sancti Spiritus:** Topes de Collantes (21°55.2'N, 80°02'W; 350 m), 10 Dec 1994, W. N. Mathis (5♂, 4♀; USNM); Topes de Collantes (21°54.4'N, 80°01.4'W; 670 m), 9-11 Dec 1994, W. N. Mathis (10♂, 9♀; USNM).

DOMINICA. Rosalie (15°22.3'N, 61°15.3'W), 23 Mar 1989, A. Freidberg (1♂, 1♀; USNM); Trafalgar Falls (15°19'N, 61°21'W), 19 Jun 1991, D. and W. N. Mathis (1♂, 1♀; USNM).

DOMINICAN REPUBLIC. **Barahona:** Baoruco (beach and river; 18°04.6'N, 71°05.5'W), 19 May 1998, D. and W. N. Mathis (4♂, 2♀; USNM); Cabral (canals E of Cabral; 18°15.2'N, 71°13.4'W), 16 May 1995, W. N. Mathis (2♂; USNM). **Distrito Nacional:** Santo Domingo (Jardín Botanico; 18°32.5'N, 69°57'W), 25 May 1998, D. and W. N. Mathis (7♂; USNM). **El Seibo:** El Seibo (5 km E; 18°44.73'N, 68°59.2'W; 120 m), 12 May 1995, W. N. Mathis (6♂, 1♀; USNM); Pedro Sáchez (18°51.4'N, 69°6.5'W), 26 May 1998, D. and W. N. Mathis (5♂, 2♀; USNM); Rincón (near; 18°45.3'N, 68°55.7'W), 12 May 1995, W. N. Mathis (1♂, 1♀; USNM). **Hato Mayor:** Hato Mayor (5.5 km E; 18°46.4'N, 69°12.5'W), 26 May 1998, D. and W. N. Mathis (3♂, 2♀; USNM). **La Vega:** Jarabacoa (1-2 km S; 19°06.9'N, 70°37'W; 520 m), 8–21 May 1995, 1998, D. and W. N. Mathis (16♂, 10♀; USNM); Jarabacoa (5 km S; 19°05.8'N, 70°36.5'W; 640 m), 8-20 May 1995, W. N. Mathis (2♀; USNM); Salto Baiguate (near Jarabacoa; 19°05.5'N, 70°36.9'W; 570 m), 9 May 1995, 1998, D. and W. N. Mathis (6♂; USNM); Salto Guasara (near Jarabacoa; 19°04.4'N, 70°42.1'W; 680 m), 9 May 1995, W. N. Mathis (9♂, 3♀; USNM); Rio Camu (3.5 km NW La Vega; 19°13.7'N, 70°35.2'W; 100 m), 10 May 1995, W. N. Mathis (2♂, 2♀; USNM). **Peravia:** Rio Ocoa (San José Ocoa; 18°31.7'N, 70°30.4'W), 21 May 1998, D. and W. N. Mathis (1♂; USNM); San José Ocoa (10 km NE; 18°35'N, 70°25.6'W), 21 May 1998, D. and W. N. Mathis (4♂; USNM). **Puerto Plata:** Rio Camu (14 km E Puerto Plata; 19°41.9'N, 70°37.5'W), 23 May 1998, D. and W. N. Mathis (3♂; USNM); Sonador (19°35.9'N, 70°36.2'W; 440 m), 18 May 1995, W. N. Mathis (1♂; USNM).

GRENADA. **St. Andrew:** Balthazar (12°07.7'N, 61°39.3'W), 19 Sep 1996, W. N. Mathis (4♂; USNM); Grand Étang (lake; 12°05.6'N, 61°41.7'W), 20 Sep 1996, W. N. Mathis (2♂, 2♀; USNM); La Force Bridges (12°07.6'N, 61°39.8'W), 13-19 Sep 1996, 1997, W. N. Mathis (6♂, 1♀; USNM). **St. George:** Beauséjour River (12°05.6'N, 61°44.3'W), 15 Sep 1996, W. N. Mathis (6♂, 1♀; USNM); Vendôme (1 km E; 12°04.8'N, 61°42.2'W), 14–17 Sep 1996, 1997, W. N. Mathis (7♂, 3♀; USNM). **St. John:** Concord Falls (12°07.1'N, 61°43'W), 14-21 Sep 1996, W. N. Mathis (9♂, 2♀; USNM); Palmiste (12°08.7'N, 61°44.4'W), 21 Sep 1996, W. N. Mathis (2♂, 1♀; USNM); Palmiste Lake (12°08.3'N, 61°44'W), 19 Sep 1996, W. N. Mathis (3♂; USNM). **St. Patrick:** Bathway Beach (12°12.6'N, 61°36.7'W), 18–20 Sep 1996, W. N. Mathis (1♀; USNM).

HAITI. Baie de Chouchou (19°49'N, 72°28.5'W), 8 Jun 1978, L. Raccurtt, R. Lowrie (1♂; USNM).

JAMAICA. **Clarendon:** Rest (3.5 km N; 17°54.1'N, 77°21.1'W), 9 May 1996, D. and W. N. Mathis, H. B. Williams (1♀; USNM); Rest (3 km N; 17°54.3'N, 77°21.4'W), 15 Apr 2000, W. N. Mathis (1♀; USNM). **Manchester:** Mandeville (18°03.5'N, 77°31.9'W), 7–13 May 1996, D. and W. N. Mathis, H. B. Williams (1♂, 2♀; USNM). **Portland:** Crystal Springs (18°12.5'N, 76°37.9'W), 18 May 1996, D. and W. N. Mathis, H. B. Williams (1♀; USNM). **St. Andrew:** Mavis Bank (1.5 km W; 18°01.4'N, 76°39.9'W), 22 Apr 2000, W. N. Mathis (2♂; USNM). **St. Elizabeth:** Brae River (18°05.2'N, 77°39.3'W), 10 May 1996, D. and W. N. Mathis, H. B. Williams (1♀; USNM); Elim (18°07.1'N, 77°40.6'W), 10 May 1996, D. and W. N. Mathis, H. B. Williams (1♂; USNM); Ys Falls (18°09.3'N, 77°49.5'W), 17–18 Apr 2000, W. N. Mathis (5♂, 3♀; USNM). **St. Thomas:** Bath Fountain Spring (17°57.6'N, 76°21.3'W), 15 May 1996, D. and W. N. Mathis, H. B. Williams (3♂; USNM).

PUERTO RICO. Adjuntas (18°09.8'N, 66°43.2'W), 22 Sep 1995, D. and W. N. Mathis (2♂, 2♀; USNM). Jayuya (2 km E; Rio Saliente; 18°12.8'N, 66°33.9'W), 22 Sep 1995, D. and W. N. Mathis (4♂, 4♀; USNM). Maricao (18°11.1'N, 66°58.9'W), 21 Sep 1995, D. and W. N. Mathis (14♂, 1♀; USNM). Maricao, Los Viveros (18°10.5'N, 66°59.2'W), 21 Sep 1995, D. and W. N. Mathis (11♂, 1♀; USNM). Mayaguez (18°12.1'N, 67°08.7'W), 15–16 Feb 1914 (1♂; paratype; ANSP). Rio Hoconuco (18°7.6'N, 67°2.6'W), 20 Sep 1995, D. and W. N. Mathis (4♂; USNM).

ST. LUCIA. Fond St. Jacques (13°50'N, 61°02'W), 13–14 Jun 1991, D. and W. N. Mathis (3♂, 1♀; USNM). Micoud (13°49'N, 60°54'W), 15 Jun 1991, D. and W. N. Mathis (7♂, 2♀; USNM). Soufrière Botanical Garden (13°51'N, 61°04'W), 12 Jun 1991, D. and W. N. Mathis (3♂, 1♀; USNM).

ST. VINCENT. **Charlotte:** Montreal (13°12'N, 61°11'W), 26 Mar-9 Jun 1989, 1991, D. and W. N. Mathis (1♂, 3♀; USNM); South Rivers (13°14.6'N, 61°09.3'W), 8 Sep 1997, W. N. Mathis (8♂; USNM); Yambou River (13°09.8'N, 61°08.7'W), 8–10 Sep 1997, W. N. Mathis (3♂; USNM). **St. Andrew:** Buccament Bay (near beach; 13°11'N, 61°16'W), 4 Sep 1997, W. N. Mathis (1♂; USNM); Vermont (13°13'N, 61°13'W), 5–8 Sep 1997, W. N. Mathis (2♂, 2♀; USNM). **St. Patrick:** Cumberland River (3 km E Spring Village; 13°15'N, 61°14'W), 10 Jun 1991, D. and W. N. Mathis (8♂, 13♀; USNM).

TRINIDAD AND TOBAGO. Tobago. **St. John:** Charlotteville (beach; 11°19.5'N, 60°32.9'W), 16–18 Apr 1994, D. and W. N. Mathis (2♂, 4♀; USNM); Charlotteville (2 km S; 11°19'N, 60°33'W), 10 Jun 1993, 1994, W. N. Mathis (2♀; USNM); Charlotteville (5 km S; 11°18.9'N, 60°34.5'W), Hermitage River and beach (11°18.9'N, 60°34.5'W), 22 Apr–11 Jun 1993, 1994, D. and W. N. Mathis (6♂, 6♀; USNM); Kings Bay Reservoir (11°17'N, 60°33'W), 15 Jun 1993, W. N. Mathis (9♂, 10♀; USNM); Parlatuvier (creek; 11°17.9'N, 60°35'W), 14 Jun 1993, W. N. Mathis (4♂, 6♀; USNM); Speyside (11°18'N, 60°32'W), 13–15 Jun 1993, W. N. Mathis (3♀; USNM); Speyside (Doctor River; 1 km NW; 11°18'N, 60°32'W), 12–13 Jun 1993, W. N. Mathis (1♂, 1♀; USNM). **St. Paul:** Delaford, Kings Bay (11°16'N, 60°32.8'W), 13 Jun 1993, W. N. Mathis (1♂; USNM); Kendall (11°14.3'N, 60°35.7'W), 21 Apr 1994, W. N. Mathis (2♂, 2♀; USNM); Roxborough (6 km NNW; 11°16'N, 60°35.4'W), 20 Apr 1994, W. N. Mathis (1♀; USNM). Trinidad. **Caroni:** San Rafael (2 km N; 10°34'N, 61°15'W; forest reserve), 22 Jun 1993, W. N. Mathis (1♂, 1♀; USNM); Talparo (2 km N, 10°31'N, 61°17'W), 22 Jun 1993, W. N. Mathis (2♂, 2♀; USNM). **St. Andrew:** Valencia (1 km W; 10°39'N, 61°13'W), Aripo River, 20 Jun 1993, W. N. Mathis (7♂, 1♀; USNM). **St. George:** Filette (1 km SE; 10°47'N, 61°21'W), Yarra River, 25 Jun 1993; W. N. Mathis (4♂, 4♀; USNM); Lalaja Road (10°43'N, 61°17'W; streamlet), 26 Jun 1993; W. N. Mathis (1♀; USNM); Marianne River (9 km S; 10°46'N, 61°18'W), 25 Jun 1993, W. N. Mathis (7♂, 5♀; USNM); Mount St. Benedict (10°39'N, 61°24'W), 18–21 Jun 1993, W. N. Mathis (1♂, 1♀; USNM); Mount St. Benedict (10°39'N, 61°24'W; creek near base), 19 Jun 1993, W. N. Mathis (8♂, 5♀; USNM); Tacariqua River, Caura Recreational area (10°43'N, 61°17'W), 21 Jun 1993, W. N. Mathis (2♂, 1♀; USNM). **Victoria:** Basse Terre (7 km E; 10°07'N, 61°14'W), 27 Jun 1993, W. N. Mathis (12♂, 6♀; USNM).

#### Distribution

**Figure 83.**
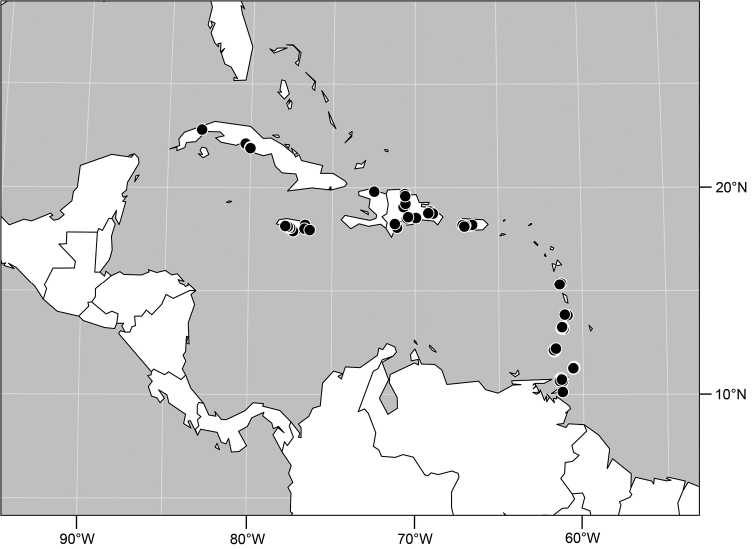
Distribution of *Polytrichophora desmata* (Williston).

(Fig. 83). Neotropical: Trinidad and Tobago, West Indies (Cuba, Dominica, Dominican Republic, Grenada, Haiti, Jamaica, Puerto Rico, St. Lucia, St. Vincent).

#### Remarks.

Williston (1896: 395), who commented on the specific locality of only one other species (*Ephydra pygmea*) from St. Vincent, noted that the holotype of *Psilopa desmata* was collected “Near the sea by open stream.”

This species belongs to the *desmata* species group, which in the New World includes only one other species, *Polytrichophora pulchra*. From the latter species, this species is distinguished by having a subshiny to shiny mesonotum that is bronzish black and the midfacies is either lacking a distinctly colored vitta or if a vitta is present then it is not extended dorsally past the midheight of the face.

### Polytrichophora pulchra

(Cresson)

http://species-id.net/wiki/Polytrichophora_pulchra

Figs 84 88

Discocerina pulchra Cresson 1918: 56.
Polytrichophora pulchra . Cresson 1946: 143 [generic combination, review of Neotropical fauna]. Wirth 1968: 8 [Neotropical catalog]. Mathis and Zatwarnicki 1995: 186 [world catalog]. Mathis 1997b: 39 [review, Belize].

#### Diagnosis.

This species is distinguished from congeners by the following combination of characters: Small shore flies, body length 1.45–1.90 mm.

*Head*: Frons mostly black with moderate dusting of gray to whitish gray microtomentum; ocellar triangle slightly lighter in color; fronto-orbits creamy white, narrow. Antenna mostly yellow; basal flagellomere darkened apicodorsally; arista bearing 5 dorsal rays. Face, especially ventrad of antennal grooves, narrow, width less than combined length of antennal scape and pedicel. Midfacies with broad vitta extended from oral margin to antennal bases, gray to yellowish gray, sharply contrasted with blackish brown, vertical laterofacies; parafacial yellowish white to entirely silvery white, contrasted with the much darker laterofacies; parafacials with little or no dilation ventrally; gena very short, height much less than height of basal flagellomere; gena-to-eye ratio 0.05–0.07.

*Thorax*: Mesonotum mostly microtomentose, dull, grayish brown to grayish tan, not subshiny to shiny; postpronotum bluish gray; pleural areas blackish brown with sparse to moderate dusting of bluish gray to brownish gray microtomentum. Lacking stout setae along anterior margin of wing; costal vein ratio 0.81–0.95; M vein ratio 0.57–0.61. Forefemur with distinct row of 5–7 moderately long, slightly larger setae along posteroventral surface, lacking stout setae along apical half of anteroventral surface; femora blackish brown; tibiae darkened dorsally, becoming yellowish ventrally and at apices; basal tarsomeres yellow, apical 1–2 brown.

**Figures 84–87.**
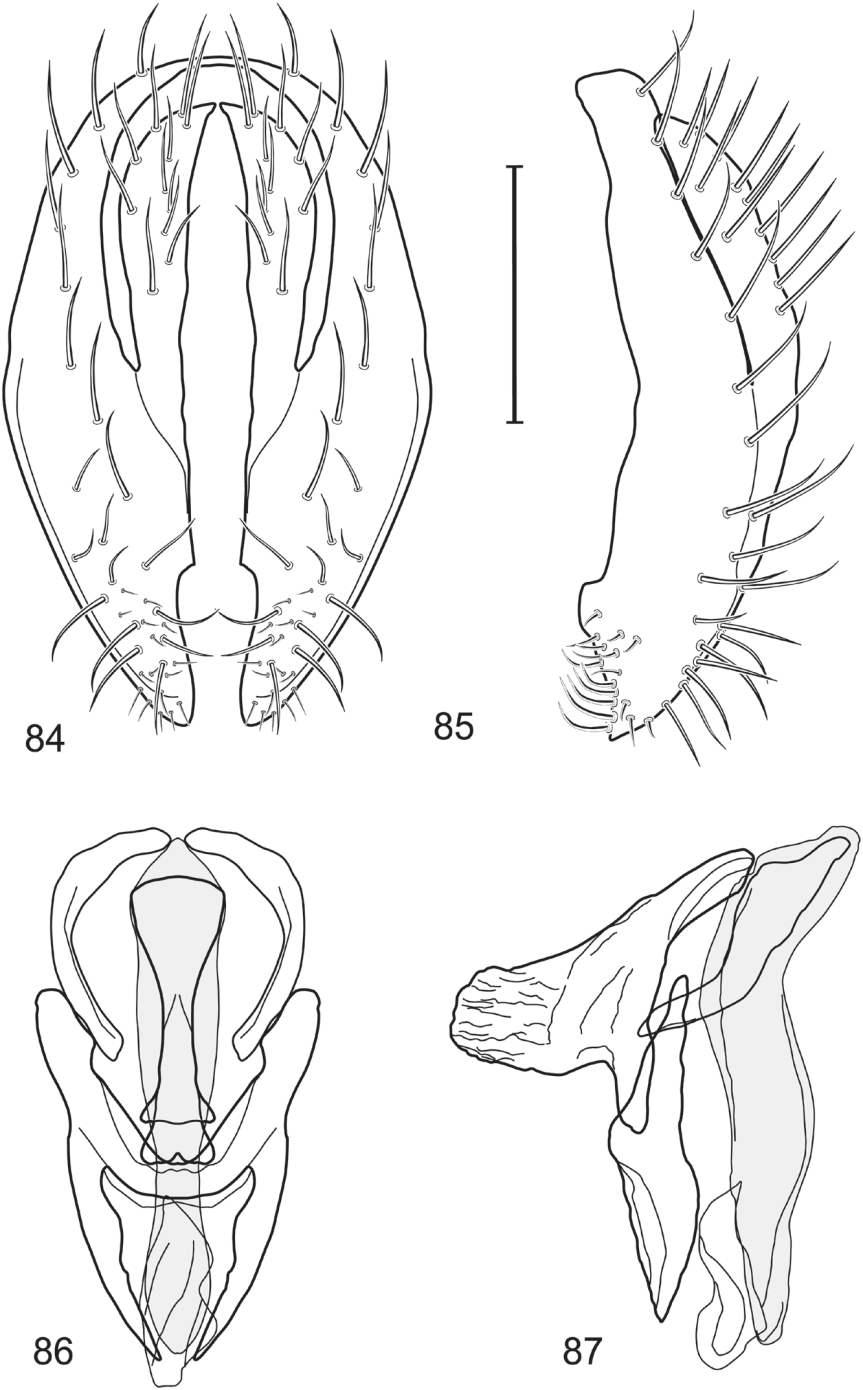
Illustration of *Polytrichophora pulchra* (Cresson) (male) (Peru. Madre dos Dios: Manu, Rio Manu, Pakitza) **84** epandrium and cerci, posterior view **85** same, lateral view **86** internal structures of male terminalia (aedeagus [shaded], phallapodeme, gonite, hypandrium), ventral view **87** same, lateral view. Scale bar = 0.1 mm.

*Abdomen*: Tergites subshiny to entirely shiny, very thinly microtomentose, brownish black; tergite 5 of male distinctly more sparsely microtomentose and shinier than tergites 1–4, black. Male terminalia (Figs 84–87): Epandrium in posterior view (Fig. 84) generally ovate, more or less uniformly setulose, dorsal margin broadly rounded, lateral margins widest a midheight, thereafter ventrally tapered medially, both ventral extensions pointed with a moderately narrow but deep gap, in lateral view (Fig. 85) narrow dorsally, becoming gradually wider ventrally, ventral apex acutely pointed and with a subapical, anterior, blunt, short projection; cercus comparatively narrow and elongate, conspicuous; aedeagus in lateral view (Fig. 87) narrowly tubular, sinuous, apex tapered to point and with a short, folded back, flap-like, apical, membranous distiphallus, in ventral view (Fig. 86) moderately narrowly elongate with lateral margins shallowly sinuous; phallapodeme in lateral view (Fig. 87) roughly triangular with distinct extended keel broadly rounded apically, in ventral view (Fig. 86) clavate with basal margin rounded truncate, with 2 bluntly clavate margins apically; gonite in lateral view (Fig. 87) comparatively robustly developed, elongate, shallowly angulate, more robustly developed toward aedeagal base, in ventral view (Fig. 86) conspicuously arched, slightly broader medially, narrowed at each apices; hypandrium large, in lateral view (Fig. 87) moderately elongate, with lateral, irregular extension, in ventral view (Fig. 86) more or less H-shaped, with arms of anterior portion oriented medially and posterior arms slightly flared laterally.

#### Type material.

The holotype female is labeled “Turrucares 22XII09 [22 Dec 1909][,] C[osta]R[ica][,]PPCalvert/Sweeping over mud/TYPE No. 6129 [pink; number handwritten]/310/Holo-TYPE Discocerina PULCHRA E. T. Cresson Jr [magenta; species and generic names handwritten].” The holotype is double mounted (minuten pin in rectangular card block), is in poor condition, and is deposited in the ANSP (6129).

#### Type locality.

Costa Rica. Alajuela: Turrucares (9°57.6'N, 84°19.2'W).

#### Other specimens examined.

Neotropical. BELIZE. **Stann Creek:** Cockscomb Basin Wildlife Sanctuary (16°47'N, 88°30'W), 5–6 Apr 1993, W. N. Mathis (3♂, 3♀; USNM); Man of War Cay, 8–15 Nov 1987, D. and W. N. Mathis (1♂, 1♀; USNM); Maya Center, Cabbage Haul Creek (16°48'N, 88°23'W), 3 Apr 1993, W. N. Mathis (9♂, 5♀; USNM); Placencia Lagoon, Rum Point (16°32.8'N, 88°22.1'W), 4–5 Nov 1987, D. and W. N. Mathis (1♀; USNM); Salt Creek (12 km N Dangriga; 17°13.4'N, 88°18.6'W), 28 Mar 1988, W. N. Mathis (2♂; USNM); Twin Cays (south end of West Island), Jan 1987, W. N. Mathis, C. Feller (1♂; USNM); Wee Wee Cay (16°45.9'N, 88°08.6'W), 24 Jan 1987, W. N. Mathis, C. Feller (1♀; USNM).

BOLIVIA. **La Paz:** Guanay (3 km E; 15°30.2'S, 67°52.3'W; 500 m), 14 Mar 2001, W. N. Mathis (10♂, 1♀; USNM); Mapiri (Rio Mapiri; 15°18.6'S, 68°13'W; 720 m), 17 Mar 2001, W. N. Mathis (4♂, 1♀; USNM); Mapiri (5 km west; 15°17.8'S, 68°15.6'W; 750 m), 16 Mar 2001, W. N. Mathis (3♂, 1♀; USNM); San Pedro (3 km NE; 16°S, 67°35.3'W; 780 m), 12 Mar 2001, W. N. Mathis (3♂, 1♀; USNM).

BRAZIL. **Amazonas.** Manaus, INPA (03°05.9'S, 59°59.1'W; 60 m), 4 May 2010, D. and W. N. Mathis (3♂, 2♀; INPA, USNM); Reserva Ducke (02°55.8'S, 59°58.5'W; 40 m), 12–28 Sep 1986, L. S. Aquino, U. Barbosa (1♂; INPA). **Pará.** Santana, Furo dos Macacos, Marajó (2°02.6'S, 47°44.6'W), Oct 1970 (1♂; MZUSP); Tucuruí, Morro do Senador (03°59.1'S, 49°44.8'W), Dec 2001, J. A. Rafael, J. Vidal (4♂, 3♀; INPA). **Paraná.** Curitiba, Universidade Federal do Paraná, Reserva Biológica (25°26.9'S, 49°14'W; 915 m), 18 Jan 2010, D. and W. N. Mathis (1♂; DZUP, USNM). **São Paulo.** Itú (23°15.9'S, 47°17.9'W), Sep 1960, M. A. V. D’Andretta (1♂; MZUSP); Ubatuba, Praia do Estaleiro (23°20.5'S, 44°53'W; beach), 30 Mar 2010, D. and W. N. Mathis (2♂; USNM); Ubatuba, Praia Puruba (23°21'S, 44°55.6'W; beach), 29 Mar 2010, D. and W. N. Mathis (1♂, 1♀; USNM).

COSTA RICA. **Cartago:** Cartago (9°51.4'N, 83°55.2'W), 25 May 1909, P. P. Calvert (1♂; ANSP); La Suiza (9°51.5'N, 83°37.5'W), 6 May 1926, P. Schild (2♂; ANSP); Pejibaye, Reserva Biológica El Copal, Ron Ron (09°47'N, 83°45'W; 1090 m), 4 Apr 2005, J. Azofeifa, D. Briceño (2♂, 3♀; INBIO). **Guanacaste:** La Fortuna, Quebrada Santa Fé (10°40.5'N, 85°12.1'W; 100–200 m), 8 Dec 2002, J. D. Gutiérez (2♂, 3♀; INBIO); Parque Nacional Santa Rosa, Bosque San Emilio (10°50.6'N, 85°36.8'W; 300 m), 6-7 Jul 2002, D. Briceño (4♂, 2♀; INBIO); Parque Nacional Santa Rosa, Camino Borrachos (10°50.6'N, 85°37'W; 300 m), 3-5 Oct 2002, D. Briceño (7♂, 7♀; INBIO). **Heredia:** Santo Domingo (INBIO Parque; 9°58.4'N, 84°5.6'W), 14 Jun 2003, D. and W. N. Mathis (4♂, 4♀; USNM). **Puntarenas:** Cabuya (9°37.8'N, 85°4.6'W), 20 Jun 2001, D. and W. N. Mathis (3♂; USNM); Dominical (9°14.8'N, 83°51.4'W; 0–2 m), 11 Jun 2003, D. and W. N. Mathis (3♂, 1♀; USNM). **San José:** Río Paraíso (9°33.8'N, 84°7.4'W; 350–400 m), 15-17 Feb 2003, D. and W. N. Mathis (9♂, 2♀; USNM).

ECUADOR. **Guayas:** Machala (NNE; 2°40'S, 79°13.4'W; 40 m), 13 Jan 1978, W. N. Mathis (1♀; USNM). **Puerto Orellana:** Rio Tiputini Biodiversity Station (0°38.2'S, 76°8.9'W), 12–26 Aug 1999, A. Baptista, M. Kotrba, W. N. Mathis (32♂, 10♀; USNM).

GUYANA. Conservation of Ecological Interactions and Biotic Associations (CEIBA; ca. 40 km S Georgetown; 6°29.9'N, 58°13.1'W), 13-21 Apr 1994, 1995, W. N. Mathis (3♂, 2♀; USNM); Kaieteur Falls (5°10.7'N, 59°29.2'W; 570 m), 7 Apr 1994, W. N. Mathis (1♂; USNM); Kaieteur Falls (5°10.5'N, 59°28.9'W), 21-24 Aug 1997, W. N. Mathis (7♂, 2♀; USNM); Karanambo, Rupununi River (ox bow; 3°45.1'N, 59°18.6'W), 2 Apr 1994, W. N. Mathis (3♂, 1♀; USNM); Karanambo, Rupununi River (ox bow; 3°45'N, 59°17.5'W; 85m), 2 Dec 2010, W. N. Mathis (7♂, 4♀; USNM); Karanambo (Rupununi River; 3°45'N, 59°18.6'W; 85m), 1-3 Dec 2010, W. N. Mathis (21♂, 2♀; USNM); Kumu River and Falls (25 km SE Lethem in Kanuku Mountains; 3°15.9'N, 59°43.6'W), 28–30 Apr 1995, W. N. Mathis (1♂, 3♀; USNM); Moco-Moco (30 km E Lethem in Kanuku Mountains; 3°18.2'N, 59°39.0'W), 3–29 Apr 1994, 1995, W. N. Mathis (5♂, 2♀; USNM); Paramakatoi (04°42'N, 59°42.8'W), 24-25 Aug 1997, W. N. Mathis (1♀; USNM).

MEXICO. **Chiapas:** Cacahoatán (7 km N; 15°03.8'N, 92°09.1'W), 22 Apr 1983, W. N. Mathis (1♀; USNM); Tejería (15°03.8'N, 92°09.1'W), 23 Feb 1998, A. Freidberg (1♂; USNM).

PANAMA. **Canal Zone:** Juan Mina (09°10'N, 79°39'W), 28 Sep 1923, R. C. Shannon (1♂; ANSP); Pedro Miguel (9°01.1'N, 79°36.7'W), 10 Apr 1933, R. C. Shannon (1♂; ANSP). **Chiriqui:** Monte Lirio (08°47.5'N, 82°49.5'W), 6 Apr 1923, R. C. Shannon (4♀; ANSP).

PERU. **Loreto:** Iquitos (14 km W; 03°52.1'S, 73°28.2'W), 21 Feb 1984, W. N. Mathis (1♂; USNM).

#### Distribution

**Figure 88.**
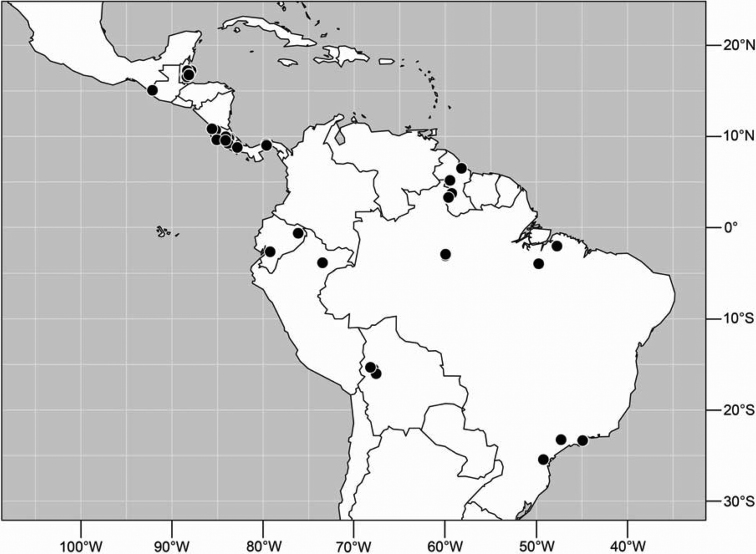
Distribution of *Polytrichophora pulchra* (Cresson).

(Fig. 88). Neotropical: Belize, Bolivia (La Paz), Brazil (Amazonas, Pará, Paraná, São Paulo), Costa Rica (Cartago, Guanacaste, Heredia, Puntarenas, San José), Ecuador (Guyana, Mexico (Chiapas), Panama, Peru (Loreto).

#### Remarks.

This species is similar to *Polytrichophora desmata*, the only other New World species in this group, and is distinguished from that species by the dull, grayish brown to brown mesonotum and by the vittate midfacies with the vitta extended from the oral margin to the antennal bases. The vitta is gray to yellowish gray and is sharply contrasted with the darker lateral portions of the midfacies.

##### The *orbitalis* Group

**Species included:** *Polytrichophora mimbres* sp. n., *Polytrichophora orbitalis* (Loew), *Polytrichophora salix* sp. n., *Polytrichophora setigera* Cresson, *Polytrichophora sturtevantorum* sp. n.

**Discussion.** The species in this group are quite similar externally and examination of structures of the male terminalia is often necessary to distinguish between them. This species group is distinguished from other groups by the following combination of characters: Parafacial 2-3 times wider ventrally; gena less high, less than 1/4 eye height; male tergite 5 lacking row of distinctive setae along posterior margin; row of stout setae along posteroventral surface of forefemur longer, subequal or longer than width of foretibia (*Polytrichophora salix* is an exception in having shorter setae); abdominal tergite 5 of male in dorsal view pointed apically.

### Polytrichophora mimbres sp. n.

urn:lsid:zoobank.org:act:C152CBD4-D477-466F-BE61-BD3D556B9DE5

http://species-id.net/wiki/Polytrichophora_mimbres

Figs 89 93

#### Diagnosis.

This species is distinguished from congeners by the following combination of characters: Small to moderately small shore flies, body length 1.85–2.25 mm.

*Head*: Frons dull to very faintly subshiny, tan with moderate investment of gray to whitish gray microtomentum; fronto-orbits narrow, gray to whitish gray. Antenna mostly yellow; basal flagellomere tannish yellow apicodorsally; arista with 5 dorsal rays. Face at narrowest point about equal to combined length of pedicel and basal flagellomere; face more densely microtomentose than frons, microtomentum faintly shiny, mostly gray to whitish gray; parafacial and gena becoming slightly more whitish to silvery white than face; parafacial becoming very slightly wider ventrally with little ventral dilation; gena moderately short, height less than height of basal flagellomere; gena-to-eye ratio 0.11–0.13.

*Thorax*: Mesonotum mostly dull to faintly subshiny, moderately densely microtomentose, mostly gray to tannish gray, completely gray laterally and anteriorly; pleural area mostly gray. Anterior margin of wing lacking spine-like setae; costal vein ratio 0.86–0.88; M vein ratio 0.50–0.56. Legs, except for tarsi but including most of coxae, gray; tibiae yellowish at apices; forefemur lacking a row of 9–10 short, stout setulae along apical half of anteroventral surface; forefemur with a row of 4–5, of moderately well-developed, evenly spaced posteroventral setae on apical half, each seta less than width of femur; tibiae mostly gray, only apices yellowish; basal tarsomeres yellow, apical 1-2 becoming slightly darker, mostly tannish yellow.

**Figures 89–92.**
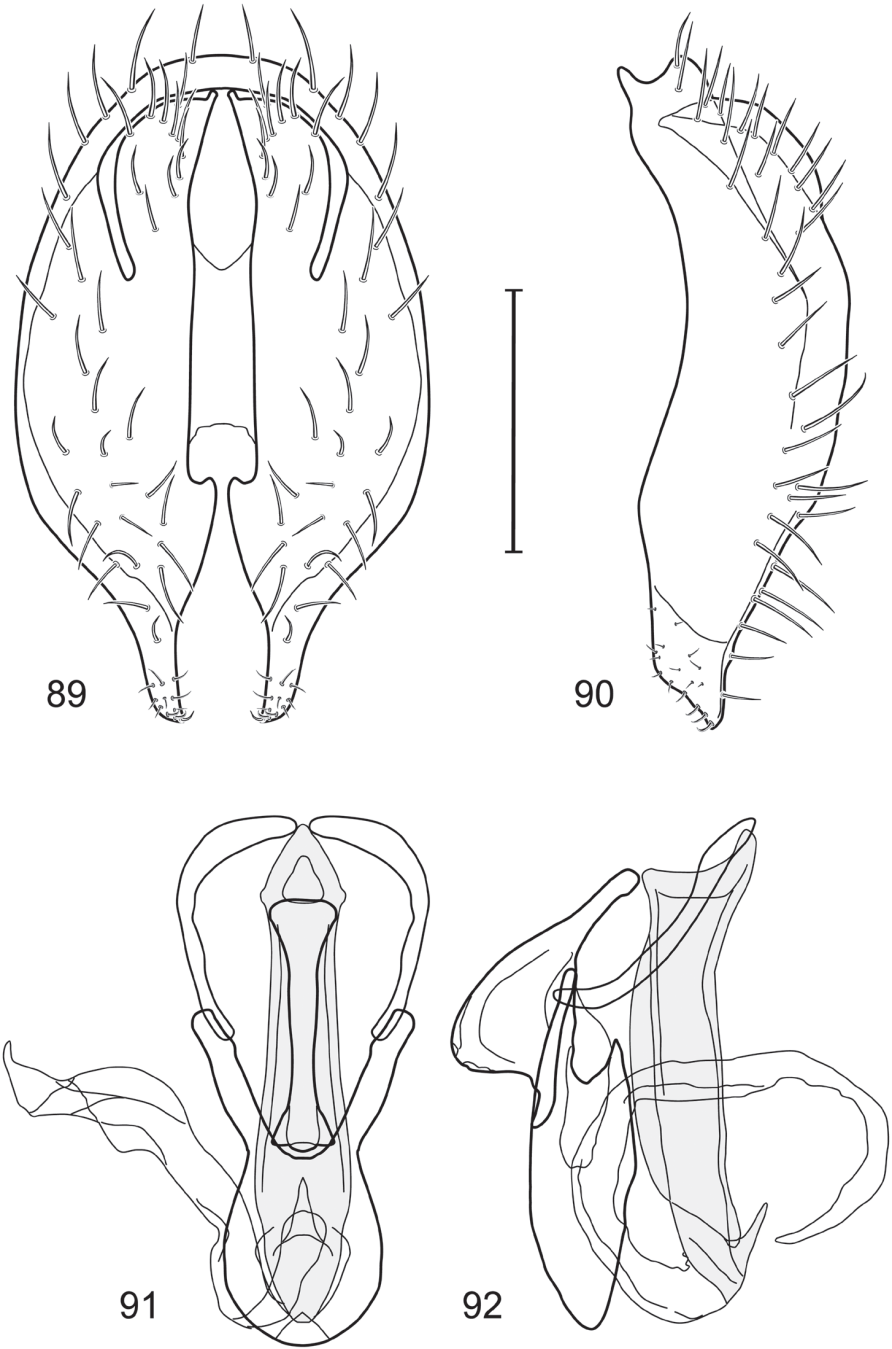
Illustration of *Polytrichophora mimbres* sp. n. (male) (Texas. Kinney: Pinto Creek) **89** epandrium and cerci, posterior view **90** same, lateral view **91** internal structures of male terminalia (aedeagus [shaded], phallapodeme, gonite, hypandrium), ventral view **92** same, lateral view. Scale bar = 0.1 mm.

*Abdomen*: Tergites dull, gray, medially with some grayish green, metallic luster; tergite 5 of male pointed apically, lacking row of 6–10, distinctly larger setae along extreme posterior margin with posterodorsal orientation. Male terminalia (Figs 89–92): Epandrium in posterior view (Fig. 89) generally elliptical, relatively uniformly setulose, dorsal margin rounded, both ventral extensions relatively wide, bluntly pointed, slightly turned medially, in lateral view (Fig. 90) with ventral apex acutely pointed, posterior margin shallowly concave; cercus large, conspicuous; aedeagus tubular but with distinct basiphallus and more membranous, more narrowly developed distiphallus, basiphallus tubular, distiphallus curved, irregularly tapered to narrow apex, in ventral view (Fig. 91) narrowly elongate with lateral margins very shallowly concave; phallapodeme in lateral view (Fig. 92) roughly triangular with extended keel truncate apically, in ventral view (Fig. 91) bluntly clavate at both apices; gonite in lateral view (Fig. 92) narrowly elongate, shallowly curved, in ventral view (Fig. 91) angularly arched, slightly wider toward base of aedeagus; hypandrium in lateral view (Fig. 92) elongate but relatively wide, in ventral view (Fig. 91) with anterior margin deeply and broadly rounded, posterior margin as lateral, parallel-sided projections.

#### Type material.

The holotype male is labeled “**USA. N[EW] M[EXICO].** Grant: Mimbres River (32°43.8'N, 107°52'W; 1665m), 22 Aug 2009, W. N. Mathis/USNM ENT 00285965 [plastic bar code label]/HOLOTYPE ♂ *Polytrichophora mimbres* Mathis & Zatwarnicki, USNM [red].” The holotype is double mounted (minuten pin in a block of plastic), is in excellent condition, and is deposited in the USNM. Forty-two paratypes (37♂, 5♀; USNM) are from the same locality as the holotype with dates from 1–22 Aug 2008, 2009.

#### Type locality.

United States. New Mexico. Grant: Mimbres River (New Mexico Highway 61 & Royal John Mine Road; 32°43.8'N, 107°52'W; 1665 m).

#### Other specimens examined.

Nearctic. UNITED STATES. COLORADO. **Rio Grande:** South Fork (37°40.2'N, 106°38.4'W; 2440 m; Malaise trap), 20 Jun 1972, W. W. Wirth (1♀; USNM).

NEVADA. **Clark:** Las Vegas Wash (36°2.3'N, 114°58.8'W), 10-11 May 2004, D. and W. N. Mathis (4♂, 7♀; USNM); Las Vegas Wash (36°5.2'N, 114°58.9'W), 10–13 May 2004, D. Mathis (1♀; USNM).

NEW MEXICO. **Catron:** Gila River (33°13.6'N, 106°15.1'W; 1750 m), 15 Aug 2007, D. and W. N. Mathis (1♂; USNM). **Grant:** Bill Evans Lake (32°52.1'N, 108°34.5'W; 1416 m), 14 Aug 2007, D. and W. N. Mathis (4♂; USNM); Silver City (Big Ditch; 32°46.4'N, 108°16.5'W; 1790 m), 14 Aug 2007, D. and W. N. Mathis (2♂; USNM). **Sandoval:** La Cueva (Junction of Highways 126 & 4; 35°52'N, 106°38.4'W; 2342 m), 14 Jun 2011, D. and W. N. Mathis (3♂, 1♀; USNM).

TEXAS. **Blanco:** Miller Creek (30°15.2'N, 98°31.7'W; 410 m), 3 Jun 2004, W. N. Mathis (2♂; USNM). **Kinney:** Del Rio (30 km E; Pinto Creek; 29°20'N, 100°31.6'W), 5 Jun 2004, W. N. Mathis (12♂, 9♀; USNM). **San Patricio:** Mathis (6.5 km S; Nueces River; 28°02.8'N, 97°51.8'W; 18 m), 5 Jun 2004, W. N. Mathis (1♂; USNM). **Travis:** Austin (Zilker Park; 30°15.8'N, 97°46.3'W), 2 Jun 2004, W. N. Mathis (2♂; USNM). **Uvalde:** Garner State Park (29°35.3'N, 99°44.3'W), 3 Apr 1955, W. W. Wirth (1♂; USNM).

UTAH. **Emery:** Green River (Green River; 38°59.6'N, 110°08.5'W; 1240 m), 31 Jul 2007, D. and W. N. Mathis (1♂; USNM); Green River (3.3 km N; 39°01.7'N, 110°09.7'W; 1253 m), 30 Jul-5 Aug 1992, 2007, D. and W. N. Mathis (10♂; USNM); San Rafael River (22.5 km SW Green River; 38°55.7'N, 110°24.5'W; 1270 m), 31 Jul 2007, D. and W. N. Mathis (5♂, 1♀; USNM). **Garfield:** Willow Tank-Hurricane Wash (37°23.2'N, 111°08'W; stock tank), 22 May 2001, D. and W. N. Mathis (1♂, 1♀; USNM). **Grand:** Thompson Spring (8.9 km N Thompson Springs; 39°02.3'N, 109°43.4'W; 1740 m), 16 Aug 2008, D. and W. N. Mathis (1♂; USNM). **Salt Lake:** Draper (40°31.6'N, 111°55.1'W; Jordan River; 1320 m), 10 May 2007, D. and W. N. Mathis (1♂; USNM). **Utah:** Thistle (40°0.4'N, 111°29.7'W; 1530 m), 11 May 2007, D. and W. N. Mathis (1♂; USNM).

#### Distribution

**Figure 93.**
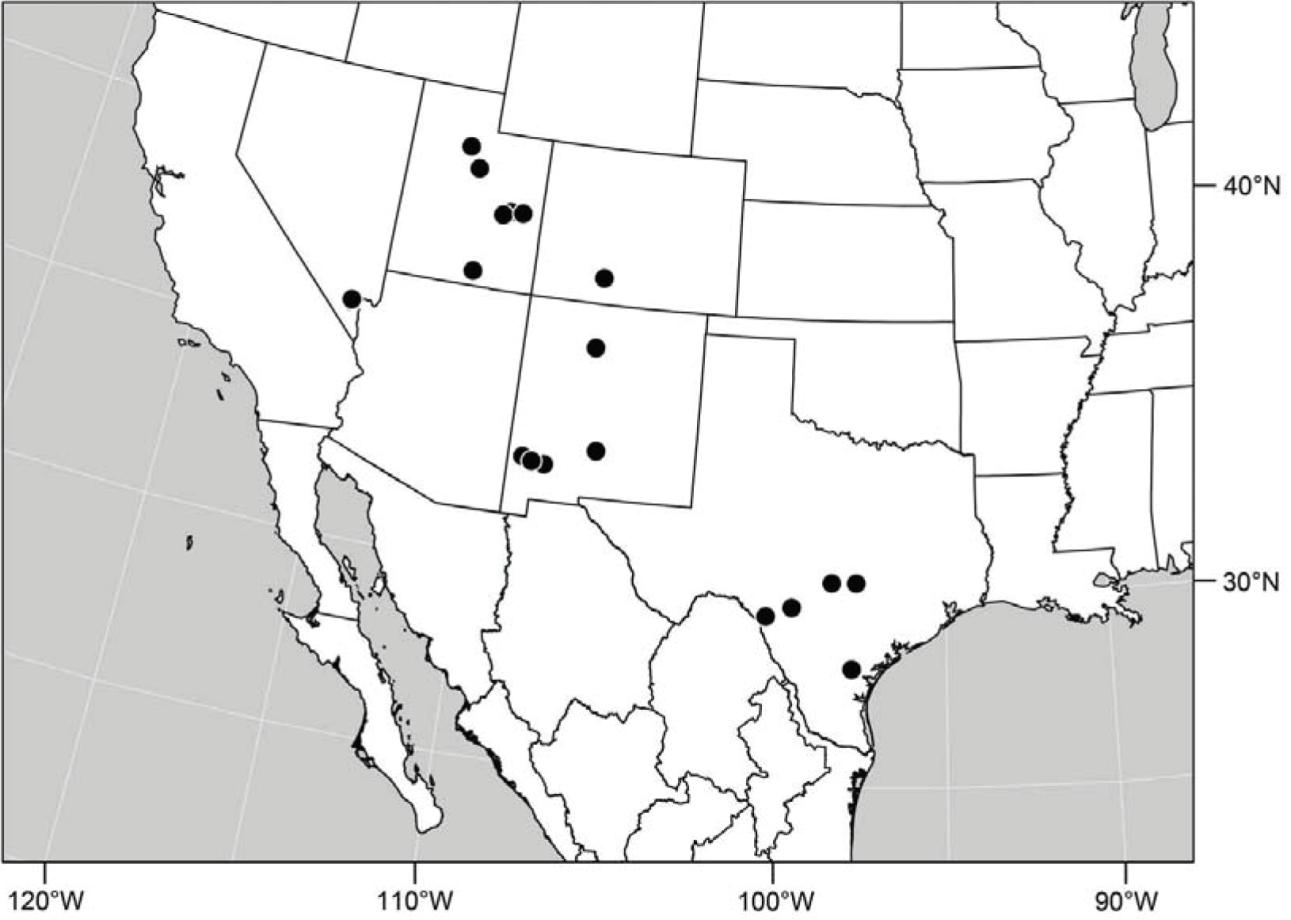
Distribution of *Polytrichophora mimbres* sp. n.

(Fig. 93). Nearctic: United States (Colorado, Nevada, New Mexico, Texas, Utah).

#### Etymology.

The species epithet, *mimbres*, is a noun in apposition and refers to the river in southern New Mexico where the type series was collected.

#### Remarks.

Among species of the *orbitalis* group, this species is distinguished by characters of the male terminalia as follows: Area between anterior arms of the hypandrium is partially sclerotized; the ventral epandrial processes are relatively wide, bluntly pointed, slightly turned medially (Fig. 89); the posteroventral edge of the epandrium in lateral view is very shallowly concave, and the taper at the apex of is more abrupt (Fig. 90).

### Polytrichophora orbitalis

(Loew)

http://species-id.net/wiki/Polytrichophora_orbitalis

Figs 94 98

Discocerina orbitalis Loew 1861: 354.
Polytrichophora orbitalis . Cresson 1924: 161 [generic combination]; 1942: 114 [review of Nearctic fauna]. Wirth 1965: 740 [Nearctic catalog]. Mathis and Zatwarnicki 1995: 185–186 [world catalog]. Zatwarnicki and Mathis 2001: 49 [figs. ♂ terminalia].

#### Diagnosis.

This species is distinguished from congeners by the following combination of characters: Small to moderately small shore flies, body length 1.40–2.10 mm.

*Head*: Frons largely dull to faintly subshiny, brown with moderate investment of gray to yellowish gray microtomentum, anterior margin with some orange coloration; fronto-orbits narrow, gray to whitish gray. Antenna mostly yellow; basal flagellomere tannish yellow apicodorsally; arista with 5 dorsal rays. Face at narrowest point about equal to combined length of pedicel and basal flagellomere; face more densely microtomentose than frons, microtomentum with faintly shiny, mostly gray to whitish gray; parafacial and gena becoming slightly more whitish to silvery white than face; parafacial becoming wider ventrally with moderate ventral dilation; gena moderately high, height less than combined length of pedicel and basal flagellomere; gena-to-eye ratio 0.11–0.13.

*Thorax*: Mesonotum mostly dull to faintly subshiny, moderately densely microtomentose, mostly tan to brown, becoming gray laterally and anteriorly; pleural area mostly gray. Anterior margin of wing lacking spine-like setae; costal vein ratio 0.81–0.95; M vein ratio 0.45–0.47. Legs, including most of coxae, yellow; forefemur lacking a row of 9–10 short, stout setulae along apical half of anteroventral surface; forefemur with a row of 4-5, of moderately well-developed, evenly spaced posteroventral setae on apical half, each seta less than width of femur; tibiae mostly gray, only apices yellowish; basal tarsomeres yellow, apical 1–2 becoming slightly darker, mostly tannish yellow.

**Figures 94–97.**
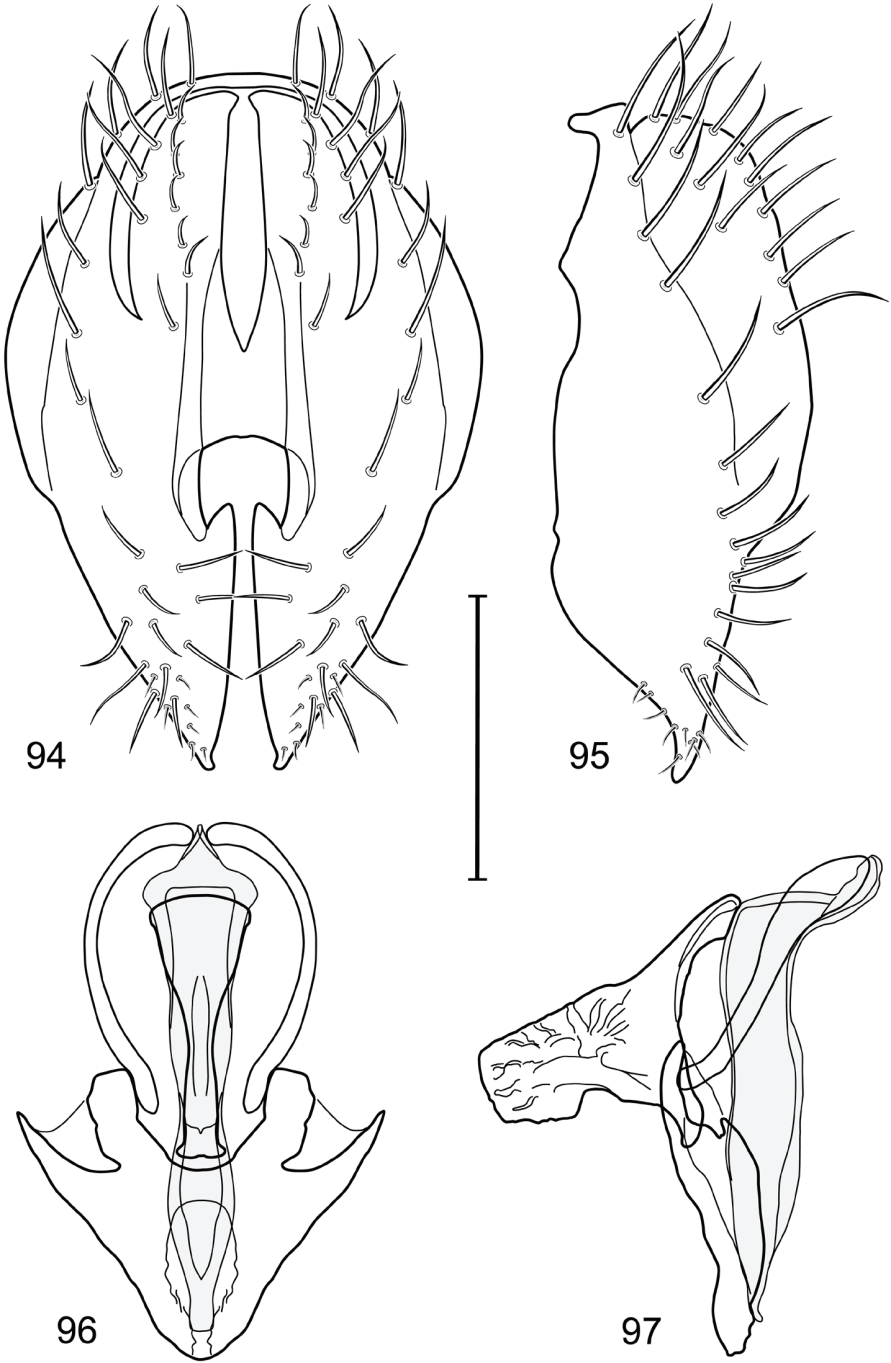
Illustration of *Polytrichophora orbitalis* (Loew) (male) (Ohio. Morgan: Oregonia) **94** epandrium and cerci, posterior view **95** same, lateral view **96** internal structures of male terminalia (aedeagus [shaded], phallapodeme, gonite, hypandrium), ventral view **97** same, lateral view. Scale bar = 0.1 mm.

*Abdomen*: Tergites dull to subshiny, gray to blackish brown; tergite 5 of male pointed apically, lacking row of 6–10, distinctly larger setae along extreme posterior margin with posterodorsal orientation. Male terminalia (Figs 94–97): Epandrium in posterior view (Fig. 94) generally elliptical, more or less uniformly setulose, dorsal margin rounded, both ventral extensions pointed, narrowly connected dorsally, in lateral view (Fig. 95) with ventral apex acutely pointed, cercus large, conspicuous; aedeagus tubular, tapered toward apex in lateral view to acute, partially recurved point, in ventral view (Fig. 96) narrowly elongate with lateral margins sinuous; phallapodeme in lateral view (Fig. 97) roughly triangular with extended keel truncate apically, in ventral view (Fig. 96) clavate with basal margin bluntly rounded; gonite in lateral view (Fig. 97) narrowly elongate, sinuous, in ventral view (Fig. 96) conspicuously arched, narrow, parallel sided; hypandrium in lateral view (Fig. 97) elongate, with lateral, irregular extension, in ventral view (Fig. 96) more or less broadly triangular, pointed anteriorly, posterior margin as 2 lateral projections, lateral one pointed, medial one wider and truncate apically.

#### Type material.

The lectotype female (not a male as Loew stated in the original description), designated herein, is labeled “[United States. District of Columbia] Loew Coll./orbitalis [handwritten]/Type 11147 [red, number handwritten]/Polytrichophora orbitalis (Lw.) det WWirth ‘[19]61 [species name handwritten, black submarginal border]/LECTOTYPE Discocerina orbitalis Loew ♀ By W. N.Mathis 1990 [species name and designator handwritten, black submarginal border].” The lectotype is double mounted (pin in a rectangular cork base), is in fair condition (pinned through base of abdomen, small amounts of verdigris, especially beneath the specimen), and is deposited in the MCZ (11147).

#### Type locality.

United States. District of Columbia: Washington (38°49.8'N, 77°00.6'W).

#### Other specimens examined.

Nearctic. CANADA. QUEBEC. Lac Meech (45°32.6'N, 73°54.2'W), 6 Jun 1960, W. W. Wirth (1♂; USNM).

UNITED STATES. CONNECTICUT. **Canaan:** Falls Village (41°57.4'N, 73°21.8'W), 29 Aug 1962, A. Stone (1♀; USNM). **Fairfield:** Putnam Park (41°02'N, 73°36.7'W), 22 Jul 1939, A. L. Melander (1♂; USNM). **Windham:** Eastford (41°53.6'N, 72°05.8'W), 25 Aug 1954, A. H. Sturtevant (1♂, 3♀; USNM).

DISTRICT OF COLUMBIA. Theodore Roosevelt Island (38°53.7'N, 77°03.7'W), 14 May 1977, W. N. Mathis (1♂; USNM); Washington (38°49.8'N, 77°00.6'W), D. L. Coquillett (1♂, 1♀; USNM).

MAINE. **Waldo:** Belfast (44°25.6'N, 69°0.4'W), 16-20 Sep 1946, C. W. Sabrosky (1♀; USNM).

MARYLAND. **Anne Arundel:** Edgewater (6 km S; 38°53'N, 76°33'W; Smithsonian Environmental Research Center), 4 Aug 1994, W. N. Mathis (4♂, 1♀; USNM). **Carroll:** Eldersburg (39°24.2'N, 76°57'W), 2 Jun 1985, J. E. Lowry, W. E. Steiner (1♀; USNM). **Frederick:** Point of Rocks (7.6 km W, 1.6 km N; 39°16.6'N, 77°31.9'W), 11 Jul 1982, G. F. and J. F. Hevel (1♂; USNM). **Garrett:** Broadford Lake (39°24.7'N, 79°22.4'W; 750 m), 20 Jun 2007, D. and W. N. Mathis (1♂, 1♀; USNM); Cranesville Swamp (39°31.9'N, 79°29.1'W; 775 m), 24 Aug 2006, D. and W. N. Mathis (1♂; USNM); Savage River (39°30.1'N, 79°06.9'W; 400 m), 23 Aug 2006, D. and W. N. Mathis (8♂, 1♀; USNM); Savage River Reservoir (near Floyd; 39°30.7'N, 79°09.3'W; 420 m), 23 Aug 2006, D. and W. N. Mathis (2♂; USNM). **Montgomery:** Cabin John (38°58.5'N, 77°09.5'W), 25 Jul 1972, G. C. Steyskal (1♂; USNM); Clarksburg, Little Bennett Regional Park (39°16'N, 77°16.8'W), 21 Sep 1990, M. J. and R. Molineaux, W. E. Steiner (2♂; USNM); Glen Echo (38°58.1'N, 77°08.4'W), 22 Aug 1922, W. L. McAtee (1♂; USNM); McKee-Beshers (39°05'N, 77°24.3'W), 7 Mar 2005, D. and W. N. Mathis (1♂, 1♀; USNM); Wheaton (39°02.3'N, 77°03.3'W), 26 Jul 1979, A. Freidberg (1♂; USNM). **Prince George'S:** Bladensburg (38°56.4'N, 76°56'W), 23 Sep 1915, R. C. Shannon (1♂; ANSP); Patuxent Wildlife Research Center (39°03'N, 76°48.2'W), 9 Jun 1967, W. W. Wirth (4♂, 3♀; USNM).

MASSACHUSETTS. **Barnstable:** Waquoit (41°35.4'N, 70°31.3'W), 16 Jul 1939, A. H. Sturtevant (1♂; USNM); Woods Hole (42°32.1'N, 70°39.6'W), 4 Sep 1950, A. H. Sturtevant (1♂; USNM).

MICHIGAN. **Midland:** Midland (43°36.9'N, 84°14.8'W), 28 Aug 1943, R. R. Dreisbach (1♂; USNM). **Wayne:** Detroit (42°21'N, 83°02.5'W), 2 May–1 Jul 1938, 1944, G. C. Steyskal (4♀; ANSP, USNM).

NEW HAMPSHIRE. **Grafton:** Franconia Notch (44°08.5'N, 71°41.3'W), 8 Jul 1931, J. M. Aldrich (1♂; USNM).

NEW YORK. **Cattaraugus:** Allegany State Park, Salamanca (42°06.4'N, 78°45.8'W), 28 May-3 Jun 1963, W. W. Wirth (1♂; USNM). **Richmond:** South Beach, Staten Island (40°35.4'N, 74°05.5'W), 7 Oct 1916, A. H. Sturtevant (1♀; USNM). **Rockland:** Nyack (W; 41°05.4'N, 73°55.1'W), 11 Jun 1939, A. L. Melander (1♀; ANSP, USNM). **Suffolk:** Cold Spring Harbor, Long Island (40°52.3'N, 73°27.4'W), Aug, A. L. Melander (1♂, 1♀; ANSP, USNM). **Sullivan:** Beaverkill (41°58.9'N, 74°50.1'W), 10 Aug 1909, E. T. Cresson, Jr. (1♀; ANSP). **Wyoming:** Letchworth State Park (42°38.5'N, 77°58.9'W), 13 Jun 1963, W. W. Wirth (3♂, 1♀; USNM).

OHIO. **Ashtabula:** Pymatuning Lake State Park (41°39.4'N, 80°27.7'W), 13 Sep 1976, B. A. Steinly (2♂, 2♀; USNM). **Highland:** Rocky Fork State Park (39°10.7'N, 83°31'W), 17 Jul 1974, J. Regensberg (1♂; USNM). **Loraine:** Beaver Creek near Amherst (41°24.2'N, 82°14'W), 22 Aug 1977, B. A. Steinly (23♂, 25♀; USNM). **Lawrence:** Vesuvius Lake (38°34.6'N, 82°37.5'W), 23 Aug 1974, J. Regensberg (6♂, 2♀; USNM). **Meigs:** Forked Run (39°03.9'N, 81°46.3'W), 27 Aug 1974, J. Regensberg (1♂, 1♀; USNM). **Mercer:** Grand Lake, St. Marys near Montezuma (40°29.6'N, 84°33'W), 26 May 1977 B. A. Steinly (2♂, 2♀; USNM). **Morgan:** Oregonia (39°04'N, 84°13.2'W), 14 Jul 1974, J. Regensberg (2♂, 5♀; USNM).

PENNSYLVANIA. **Allegheny:** Jacks Run, Allegheny, 14 Jun 1908 (1♂; ANSP). **Delaware:** Swarthmore (39°54.1'N, 75°21.1'W), 19 Jun-16 Aug 1908, 1920, E. T. Cresson, Jr. (11♂, 9♀; ANSP). **Erie:** Presque Isle State Park, Thompson'S Bay (42°08.3'N, 80°07'W), 6 May 1977, B. A. Steinly (1♂, 1♀; USNM).**Montgomery:** Lansdale (40°14.5'N, 75°17.1'W), 12 Jul 1908 (1♀; ANSP); Narberth (3.2 km N; 40°10.5'N, 75°15.6'W), 9 Sep 1915, E. T. Cresson, Jr. (1♂; ANSP).

VIRGINIA. **Accomack:** Assateague Island, near Refuge headquarters (37°54.5'N, 75°21.6'W), 15 Jun–3 Oct 2005, 2007, D. and W. N. Mathis (13♂, 4♀; USNM). **Arlington:** Barcroft Park, 4-Mile Run (38°50.8'N, 77°06.1'W), 9 Oct 2006, H. B. Williams (1♂; USNM). **Chesterfield:** Pocahontas State Park (37°23.1'N, 77°32.4'W), 11 May 2002, D. and W. N. Mathis (1♂; USNM). **Culpeper:** Lake Pelham (38°27.8'N, 78°02.7'W), 28 Apr 2006, D. and W. N. Mathis (9♂, 3♀; USNM). **Fairfax:** Dead Run (mouth; 38°58'N, 77°10.4'W), 24 Apr 2006, D. and W. N. Mathis (9♂, 10♀; USNM); Great Falls (Clay Pond; 39°00.1'N, 77°15.4'W), 23 Apr–13 Sep 2006, 2007, D. and W. N. Mathis, H. B. Williams, T. Zatwarnicki (51♂, 18♀; USNM); Great Falls (quarry; 38°59.1'N, 77°14.8'W; 50 m), 23 Apr 2007, D. and W. N. Mathis (1♂, 1♀; USNM); Great Falls (swamp trail; 38°59.4'N, 77°15.2'W), 12 May 2006, D. and W. N. Mathis (11♂, 2♀; USNM); Great Falls (Potomac River; 39°0.2'N, 77°15.2'W), 13 Sep 2007, D. and W. N. Mathis, H. B. Williams (1♂; USNM); Great Falls (Patowmack Canal; 39°00.1'N, 77°15.2'W), 27 Jun–29 Aug 2006, 2007, D. and W. N. Mathis (21♂, 7♀; USNM); Great Falls (quarry; 38°59.1'N, 77°14.8'W; 50 m), 13 Jun 2007, D. and W. N. Mathis (4♂; USNM); Turkey Run (38°57.8'N, 77°09.4'W), 4 May 2006, D. and W. N. Mathis (6♂, 2♀; USNM); Turkey Run (mouth; 38°57.9'N, 77°09.4'W), 20 Apr-17 Sep 2006, 2007, D. and W. N. Mathis, H. B. Williams, T. Zatwarnicki (85♂, 15♀; USNM). **Grayson:** Galax (15 km S; Chestnut Creek; 36°34.9'N, 80°53.5'W), 28 Jun 1986, W. E. Steiner (1♂, 1♀; USNM). **Henry:** Martinsville Reservoir (36°44.7'N, 79°52.2'W), 17 May 2005, D. and W. N. Mathis (1♂; USNM). **Madison:** Criglersville (1.6 km W; 38°28.4'N, 78°19.9'W; 185 m; Robinson River), 19 May–1 Jul 2005, D. and W. N. Mathis (1♂, 2♀; USNM). **Middlesex:** Dragon Run (37°38'N, 76°41.7'W), 17 Sep 2004, D. and W. N. Mathis (10♂, 3♀; USNM). **Patrick:** Meadows of Dan (36°44.2'N, 80°22.9'W), 18 May 2005, D. and W. N. Mathis (3♂, 5♀; USNM). **Prince William:** Prince William Forest Park: (38°34'N, 77°22'W; Carter Pond), 13 Aug 1993, D. and W. N. Mathis (2♂, 1♀; USNM); Prince William Forest Park, South Quantico Creek (shoreline, 38°34'N, 77°22'W), 10 Jul-13 Aug 1993, D. and W. N. Mathis (18♂, 21♀; USNM). **Roanoke:** Salem (Roanoke River; 37°16.1'N, 80°02.2'W; 300 m), 23 Sep 2007, D. and W. N. Mathis (4♂, 1♀; USNM). **Spotsylvania:** Rappahannock River (38°18.8'N, 77°32.5'W), 15 Apr-14 Aug 2006, 2007, D. and W. N. Mathis (11♂, 6♀; USNM). **Stafford:** Aquia Creek (38°29.1'N, 77°23.8'W), 6 Jun 2005, D. and W. N. Mathis (1♀; USNM); Aquia Harbour (38°27.9'N, 77°23.3'W), 15 May–21 Jul 1988, 2000, D. and W. N. Mathis (29♂, 7♀; USNM); Aquia Harbour, Lions Park (38°27'N, 77°23.3'W), 10 Apr-4 Nov 2003, 2005, 2006, 2007, D. and W. N. Mathis (32♂, 7♀; USNM); Curtis Park Lake (38°26'N, 77°33.3'W), 2 Jul 2005, D. Mathis (1♀; USNM); Falmouth (38°19.2'N, 77°28.1'W; Rappahannock River; 9 m), 30 Jun 2007, D. and W. N. Mathis (3♂; USNM); Lunga Reservoir (38°32'N, 77°27.4'W), 27 Apr 2006, D. and W. N. Mathis (1♀; USNM). **Westmoreland:** Westmoreland State Park (38°09.7'N, 76°51.9'W), 16 Sep 1994, D. and W. N. Mathis (3♂, 2♀; USNM). **Independent Cities:** Fredericksburg (Rappahannock River; 38°18.3'N, 77°27.5'W), 14 Apr 2006, D. and W. N. Mathis (1♂, 5♀; USNM); Norfolk (36°50.8'N, 76°17.1'W), 14 Aug 1969, G. C. Steyskal (1♂; USNM).

WEST VIRGINIA. **Hardy:** Baker (39°02.5'N, 78°44.9'W; 405 m), 12 Jul 2007, D. and W. N. Mathis (2♂, 1♀; USNM); Mathias (38°52.6'N, 78°52'W; 465 m), 13 Jul 2007, D. and W. N. Mathis (1♂, 1♀; USNM); Trout Pond (38°57.4'N, 78°44.2'W; 595 m), 13 Jul 2007, D. and W. N. Mathis (5♂; USNM). **McDowell:** Roderfield (37°26.7'N, 81°42.2'W; 335 m), 25 Sep 2007, D. and W. N. Mathis (1♂, 1♀; USNM). **Mercer:** Ceres (Kee Reservoir; 37°18.4'N, 81°10.4'W; 757 m), 24 Sep 2007, D. and W. N. Mathis (1♂; USNM). **Summers:** Bluestone State Park (37°36.7'N, 80°56.1'W; 440 m), 26 Sep 2007, D. and W. N. Mathis (1♂; USNM).

#### Distribution

**Figure 98.**
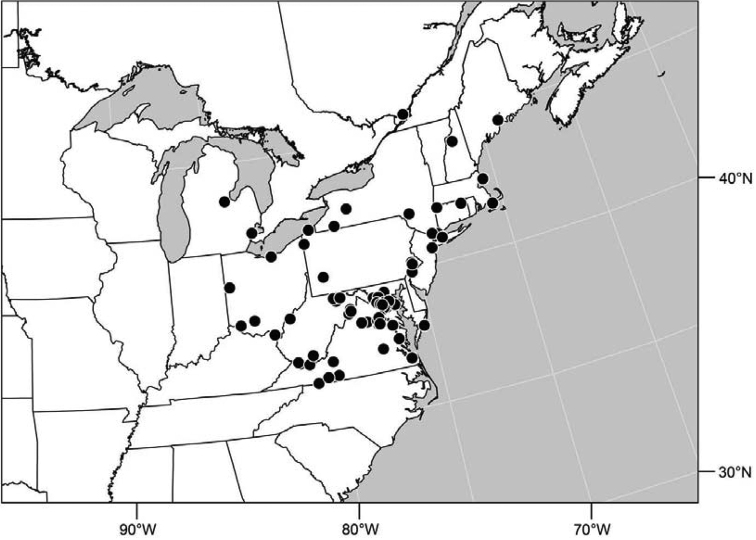
Distribution of *Polytrichophora orbitalis* (Loew).

(Fig. 98). Nearctic: Canada (Quebec), United States (Connecticut, District of Columbia, Maine, Maryland, Massachusetts, Michigan, New Hampshire, New York, Ohio, Pennsylvania, Virginia, West Virginia).

#### Remarks.

This is the nominate species of the *orbitalis* group and is distinguished by characters of the male terminalia as follows: Area between anterior arms of the hypandrium membranous; the ventral margin of the epandrium is abruptly tapered to apex; and the hypandrium is small and weakly sclerotized (Figs 94–97).

### Polytrichophora salix sp. n.

urn:lsid:zoobank.org:act:75880734-F118-407A-A1FC-7EECDAF6D1FA

http://species-id.net/wiki/Polytrichophora_salix

Figs 99 103

#### Diagnosis.

This species is distinguished from congeners by the following combination of characters: Small to moderately small shore flies, body length 1.70–2.30 mm.

*Head*: Frons dull, moderately to heavily microtomentose, mesofrons grayish brown to grayish black, more grayish anteriorly and on ocellar tubercle; parafrons black with gray microtomentum, slightly grayer anteriorly; fronto-orbits silvery gray microtomentum. Antenna short, mostly yellowish orange but black dorsally, basal flagellomere short, length subequal to height of pedicle, slightly darker, more brownish yellow; arista with 5 dorsal rays. Face wider than combined length of pedicel and basal flagellomere; face densely microtomentose, seriaceus, yellowish to lightly golden white; parafacial and gena mostly faintly golden white, becoming silvery white; parafacial with moderate ventral dilation; gena moderately short, height subequal to height of basal flagellomere, eye-to-cheek ratio 0.19–0.22.

*Thorax*: Mesonotum mostly subshiny, moderately densely microtomentose, mostly brown but becoming grayer anterolaterally; pleural areas mostly gray. Wing generally infumate, light to dark brown; anterior margin of wing lacking spine-like setae; costal vein ratio 0.68–0.71; M vein ratio 0.50–0.56. Forefemur lacking row of 9–10 short, stout setae along apical half of anteroventral surface; forefemur with row of 7–9, longer, evenly spaced posteroventral setae; femora and tibia mostly gray, apices slightly yellowish; tarsi yellow, especially ventrally, often blackish dorsally, apical 1–2 tarsomeres brown.

**Figures 99–102.**
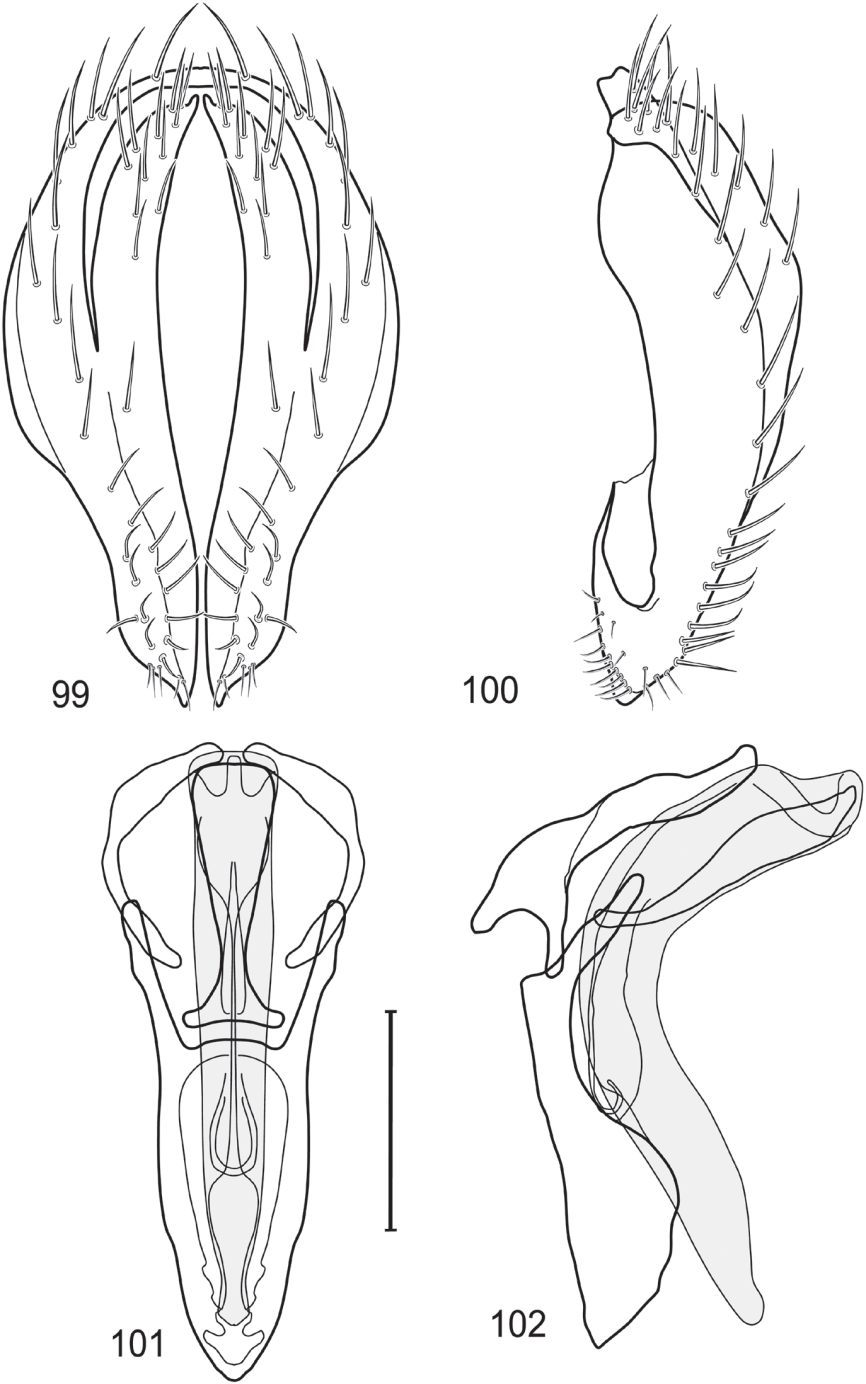
Illustration of *Polytrichophora salix* sp. n. (male) (Idaho. Kootenai: Fernan Lake) **99** epandrium and cerci, posterior view **100** same, lateral view **101** internal structures of male terminalia (aedeagus [shaded], phallapodeme, gonite, hypandrium), ventral view **102** same, lateral view. Scale bar = 0.1 mm.

*Abdomen*: Subshiny blackish gray; tergites 1–3 about equal, shorter than tergites 4–5; dorsum of male tergite 5 entirely black, similar to preceding tergites, posterior margin bluntly rounded to truncate, not bearing row of 6–10, distinctly larger setulae along posterior margin with posterodorsal orientation; sternites 4–5 of male without dense row of setulae along posterior margin. Male terminalia (Figs 99–102): Epandrium in posterior view (Fig. 99) generally as an inverted pear, pyriform, widest a midheight, conspicuously narrowed on ventral 1/3, uniformly setulose, dorsal margin rounded, both ventral extensions robustly developed, with short, acute point apicomedially, in lateral view (Fig. 100) generally shallowly arched, ventral apex moderately acutely pointed, cercus large, conspicuous; aedeagus tubular, in lateral view (102) distinctly L-shaped medially, apex tapered to acute point, in ventral view (Fig. 101) elongate, straight, tapered toward apex, lateral margins nearly straight; phallapodeme in lateral view (Fig. 102) elongate, keel in lateral view shallowly developed apically as a narrow, almost digitiform projection, in ventral view (Fig. 101) bluntly clavate at attachment with aedeagal base, opposite end T-shaped; gonite in lateral view (Fig. 102) narrowly elongate, shallowly curved, in ventral view (Fig. 101) angularly arched, slightly wider toward base of aedeagus; hypandrium in lateral view (Fig. 102) moderately elongate with long, digitiform posterior projection, in ventral view (Fig. 101) H-shaped with anterior extensions abutting anteriorly, posterior arms digitiform projections slightly flared laterally.

#### Type material.

The holotype male is labeled “**USA A[LAS]K[A].** Mat[anuska]-Su[sitna]: Willow Creek (61°46.1'N, 150°04.2'W; 50 m), 10 July 2006,D.&W.N.Mathis/USNM ENT 00285962 [plastic bar code label]/HOLOTYPE ♂ *Polytrichophora salix* Mathis & Zatwarnicki, USNM [red].” The holotype is double mounted (minuten pin in a block of plastic), is in excellent condition, and is deposited in the USNM. Seven paratypes (4♂, 3♀; USNM) bear the same label data as the holotype. Other paratypes are as follows: ALASKA. **Fairbanks North Star:** Fairbanks, Lake Ballaine (64°52.2'N, 147°49.5'W; 160 m), 2 Aug 2011, D. and W. N. Mathis (1♀; USNM). **Kenai Peninsula:** Homer (59°38.8'N, 151°31.5'W), 2 Aug 2002, D. and W. N. Mathis (2♂; USNM). **Matanuska-Susitna:** Talkeetna (62°18.9'N, 150°06.3'W), 4 Aug 2003, D. and W. N. Mathis (1♀; USNM); Willow Creek (61°46.1'N, 150°04.2'W; 50 m), 10 Jul 2006, D. and W. N. Mathis (5♂, 3♀; USNM). **Valdez-Cordova (Census Area):** Gulkana River (19.3 km N Glennallen; 62°16.1'N, 145°23.1'W), 7 Aug 2011, D. and W. N. Mathis (1♀; USNM); Valdez (4.8 km N; 61°05.8'N, 146°14.6'W), 8 Jul 2006, D. and W. N. Mathis (1♀; USNM).

**Type locality.** United States. Alaska. Matanuska-Susitna: Willow Creek (61°46.1'N, 150°04.2'W; 50 m).

Other Specimens Examined–Nearctic. UNITED STATES. ALASKA. **Nome (Census Area):** Pilgrim Hot Springs (65°05.6'N, 164°55.6'W), 3 Aug 2012, D. and W. N. Mathis (1♂; USNM).

IDAHO. **Kootenai:** Fernan Lake (47°40.5'N, 116°43.6'W), 16 Jul 1968, W. N. Mathis (1♂; USNM).

NEW MEXICO. **Sandoval:** La Cueva (Junction of Highways 126 & 4; 35°52'N, 106°38.4'W; 2342 m), 14 Jun 2011, D. and W. N. Mathis (1♂; USNM).

OREGON. **Benton:** Cary'S Grove (44°22.6'N, 123°36.1'W), 2 Sep 1974, W. N. Mathis (3♂, 1♀; USNM); Rock Creek (6.4 km SW Philomath; 44°30.1'N, 123°26.2'W), 29 May 1972, W. N. Mathis (2♀; USNM). **Linn:** Waterloo (44°29.6'N, 122°49.5'W), 24 Jul 1974, W. N. Mathis (1♂; USNM).

#### Distribution

**Figure 103.**
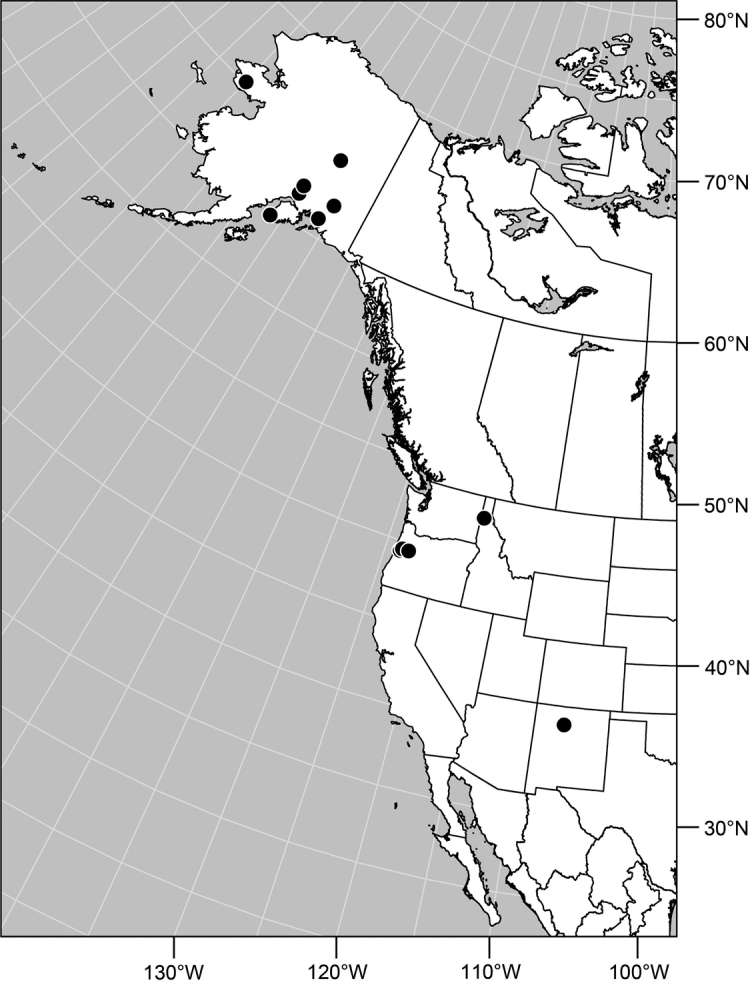
Distribution of *Polytrichophora salix* sp. n.

(Fig. 103). Nearctic: United States (Alaska, Idaho, New Mexico, Oregon).

#### Etymology.

The species epithet, *salix*, is the Latin word for willow and refers to the type locality in Alaska, Willow Creek.

#### Remarks.

This species is distinguished from congeners of the *orbitalis* group externally by having the row of stout setae along the posteroventral surface of the forefemur short (length less than width of foretibia) and male tergite five is bluntly rounded to truncate. Among characters of the male terminalia that distinguish this species are the following: Epandrium in posterior view (Fig. 99) generally as an inverted pear, pyriform, widest a midheight; both ventral epandrial extensions are robustly developed, with short, acute point apicomedially; hypandrium in ventral view (Fig. 101) H-shaped with anterior extensions abutting anteriorly, posterior arms as digitiform projections that are slightly flared laterally.

### Polytrichophora setigera

(Cresson)

http://species-id.net/wiki/Polytrichophora_setigera

Figs 104 108

Discocerina setigera Cresson 1916: 148.
Polytrichophora setigera . Cresson 1942: 114 [generic combination, review of Nearctic fauna]. Wirth 1965: 740 [Nearctic catalog]. Mathis and Zatwarnicki 1995: 186 [world catalog].
Polytrichophora orbitalis of authors, not Loew [misidentification]. Zatwarnicki and Mathis 2001: 49 [illustration of male terminalia from Jackson County, Oregon].

#### Diagnosis.

This species is distinguished from congeners by the following combination of characters: Small shore flies, body length 1.60–1.95 mm.

*Head*: Frons largely dull to faintly subshiny, brown with moderate investment of gray microtomentum, anterior margin mostly concolorous, some specimens faintly orange; fronto-orbits narrow, gray to whitish gray. Antenna mostly yellow; basal flagellomere tannish yellow apicodorsally; arista with 5 dorsal rays. Face at narrowest point about equal to combined length of pedicel and basal flagellomere; face more densely microtomentose than frons, microtomentum with faintly shiny, mostly gray to whitish gray; parafacial and gena becoming slightly more whitish to silvery white than face; parafacial becoming wider ventrally with moderate ventral dilation; gena moderately high, height less than combined length of pedicel and basal flagellomere; gena-to-eye ratio 0.13–0.16.

*Thorax*: Mesonotum mostly dull to faintly subshiny, moderately densely microtomentose, mostly tan to brown, becoming gray laterally and anteriorly; pleural area mostly gray. Anterior margin of wing lacking spine-like setae; costal vein ratio 0.72–0.77; M vein ratio 0.48–0.55. Legs, including most of coxae, yellow; forefemur lacking a row of 9–10 short, stout setulae along apical half of anteroventral surface; forefemur with a row of 4–5, moderately well-developed, evenly spaced posteroventral setae on apical half, each seta less than width of femur; tibiae mostly gray, only apices yellowish; basal tarsomeres yellow, apical 1–2 becoming slightly darker, mostly tannish yellow.

**Figures 104–107.**
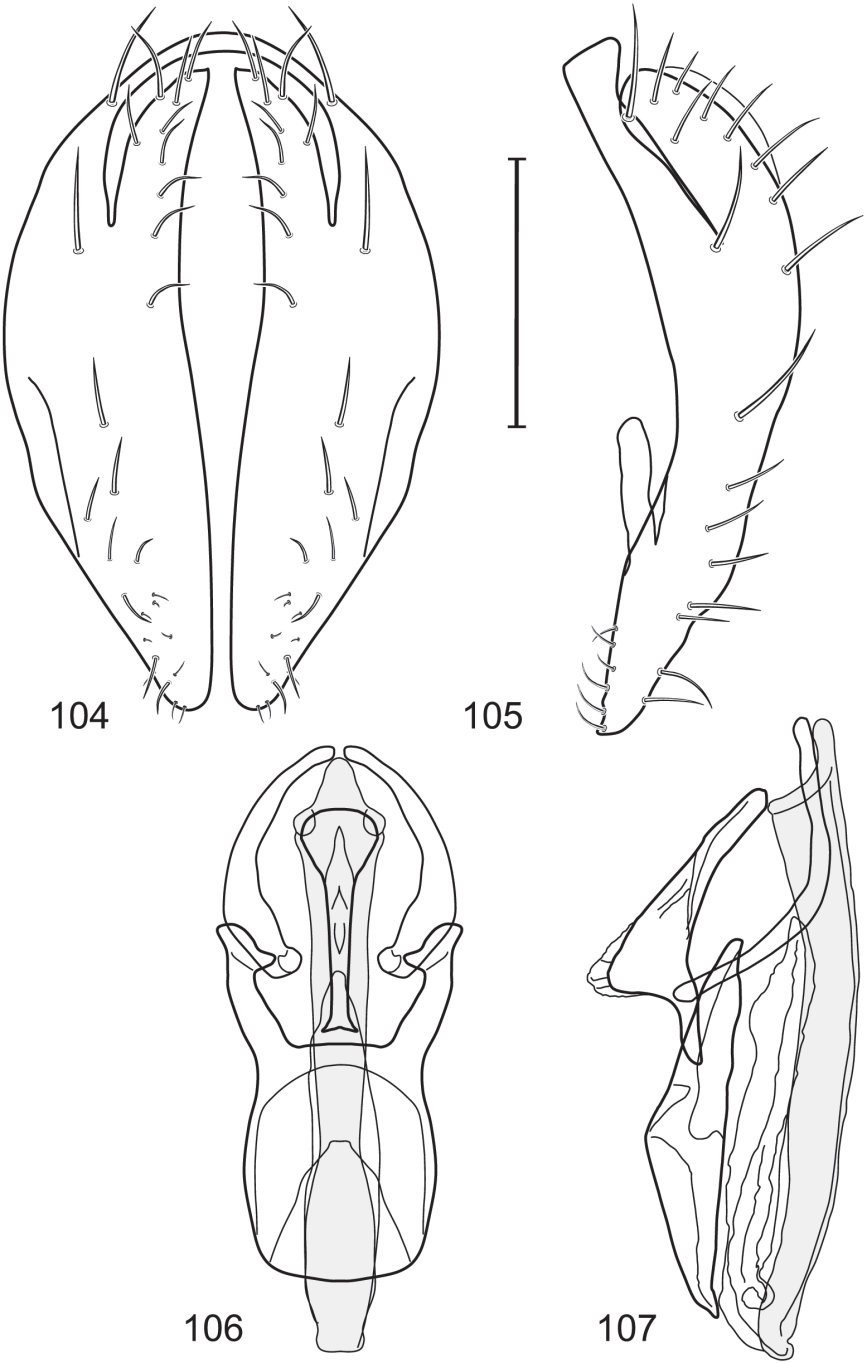
Illustration of *Polytrichophora setigera* (Cresson) (male) (Idaho. Chatcolet) **104** epandrium and cerci, posterior view **105** same, lateral view **106** internal structures of male terminalia (aedeagus [shaded], phallapodeme, gonite, hypandrium), ventral view **107** same, lateral view. Scale bar = 0.1 mm.

*Abdomen*: Tergites subshiny with faint metallic tannish to dark gray luster medially, laterally gray; tergite 5 of male pointed, lacking distinctive row of setulae apically. Male terminalia (Figs 104-107): Epandrium in posterior view (Fig. 104) generally elliptical, more or less uniformly setulose, although slightly clumped dorsally and ventrally, dorsal margin broadly rounded, both ventral extensions pointed, narrowly connected dorsally, in lateral view (Fig. 105) with ventral apex acutely pointed; cercus relatively small but conspicuous; aedeagus tubular, in lateral view (Fig. 107) slightly tapered toward apex to bluntly rounded apex, in ventral view (Fig. 106) narrowly elongate with lateral margins very shallowly sinuous; phallapodeme in lateral view (Fig. 107) roughly triangular with extended keel bluntly rounded apically, in ventral view (Fig. 106) clavate with basal margin bluntly rounded; gonite in lateral view (Fig. 107) narrowly elongate, rounded angulate, angle obtuse, in ventral view (Fig. 106) conspicuously obtusely angulate, narrow, almost parallel sided; hypandrium in lateral view (Fig. 107) elongate, with lateral, irregular extension, in ventral view (Fig. 106) more or less as a rounded square with posterior, lateral arms.

#### Type material.

The holotype male is labeled “MesaGrande[,] SonomaCo[,] V 1908 Cal[ifornia]/JPBaumberger Collector/♂/Holo-TYPE Discocerina SETIGERA E. T. Cresson Jr [magenta; species and generic names handwritten]. The holotype is double mounted (minute pin in rectangular card), is in good condition, and is deposited in the ANSP (6101).

#### Type locality.

United States. California. Sonoma: Mesa Grande (32°56.6'N, 117°11.1'W).

#### Other specimens examined.

Nearctic. UNITED STATES. CALIFORNIA. **Alameda:** Berkeley Hills (37°53'N, 122°14.3'W), 11 Apr 1908 (3♂; ANSP). **San Francisco:** San Francisco (37°47.3'N, 122°29.7'W), 27 May 1908, F. E. Blaisdell (1♂; ANSP).

OREGON. **Harney:** Page Springs (42°47'N, 118°51'W; 1300 m), 6 Aug 2005, D. and W. N. Mathis (6♂, 2♀; USNM); Pike Creek (42°34.5'N, 118°31.7'W; 1320 m), 7 Aug 2005, D. and W. N. Mathis (2♂, 2♀; USNM). **Jackson:** Rogue Elk Park, Rogue River, (ca. 8 km NE Shady Cove; 42°39.4'N, 122°46.3'W), 28 Jul 1974, P. H. Arnaud, Jr. (1♂; USNM). **Union:** North Powder (3 km N; 45°03'N, 117°55.2'W), 13 Jul 1988, W. N. Mathis (1♀; USNM).

WASHINGTON. **Asotin:** Snake River at Grande Ronde River (48°04.8'N, 116°58.9'W;), 9 May 1915, A. L. Melander (1♂; ANSP). **Franklin:** Columbia River (NW Pasco; 46°21.5'N, 119°15.5'W), 29 Jul 1998, D. and W. N. Mathis (1♂; USNM); Pasco (3.2 km E; 46°15'N, 119°07'W), 8 Aug 1975, W. N. Mathis (1♀; USNM); Ringold (NW Pasco: 46°30.4'N, 119°15.3'W), 2-29 Jul 1988, 1998, D. and W. N. Mathis (8♂, 1♀; USNM); Sacajawea State Park (46°12'N, 119°02.4'W; 100 m), 12 Jul 1988, D. and W. N. Mathis (1♀; USNM).

#### Distribution

**Figure 108.**
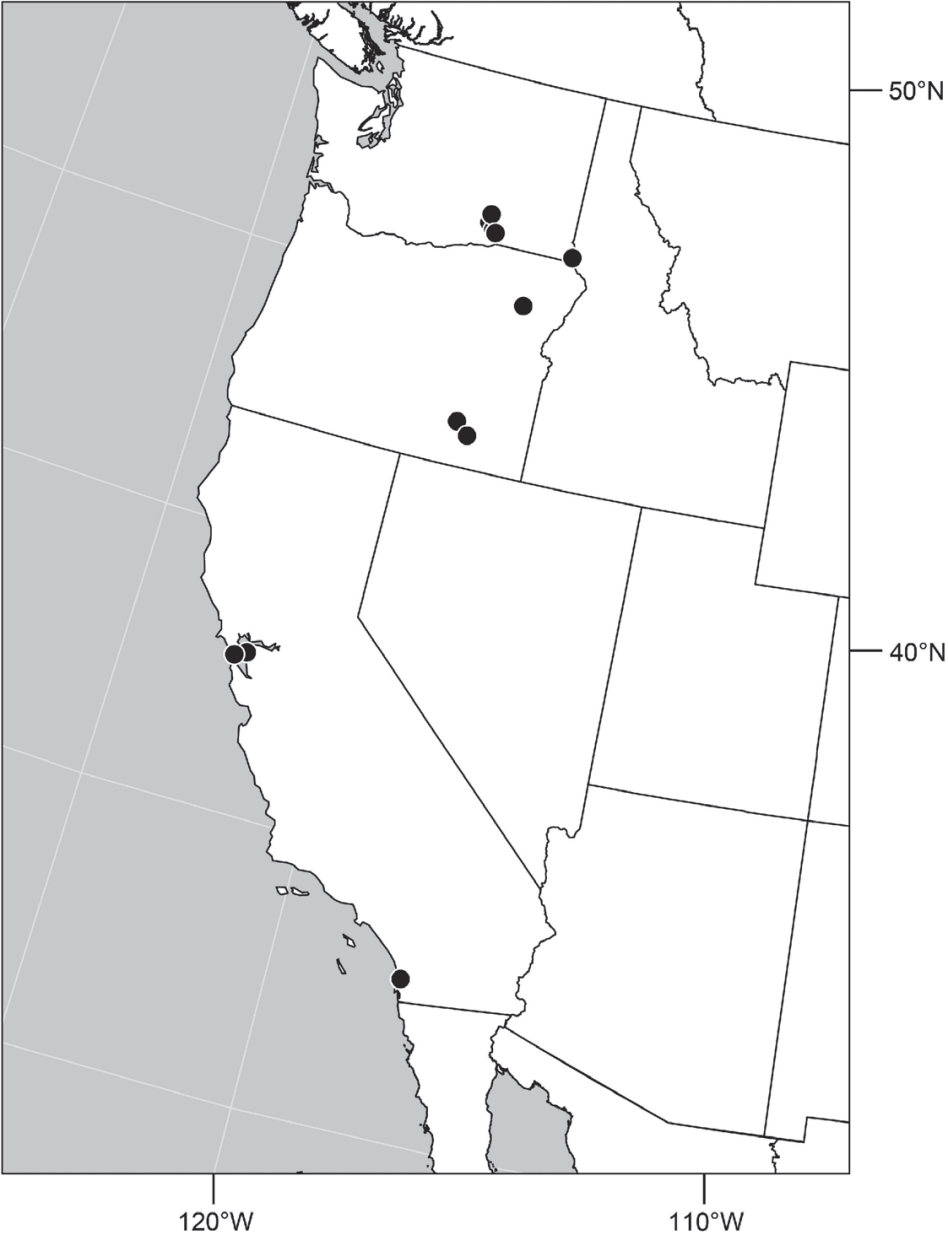
Distribution of *Polytrichophora setigera* (Cresson).

(Fig. 108). Nearctic: United States (California, Oregon, Washington).

#### Remarks.

Among congeners of the *orbitalis* group, this species is distinguished by characters of the male terminalia as follows: Area between anterior arms of the hypandrium membranous; the ventral margin of the epandrium is gradually tapered to apex; the hypandrium is large and more heavily sclerotized (Figs 104–107).

### Polytrichophora sturtevantorum sp. n.

urn:lsid:zoobank.org:act:38956C1E-B145-4512-BD77-FE2F22A9AE72

http://species-id.net/wiki/Polytrichophora_sturtevantorum

Figs 109 113

Polytrichophora orbitalis of authors, not Loew [misidentification]. Deonier 1964: 121 [key, Iowa, as *Discocerina orbitalis*]; 165:502 [ecological observations, as *Discocerina orbitalis*].

#### Diagnosis.

This species is distinguished from congeners by the following combination of characters: Small shore flies, body length 1.20–1.60 mm.

*Head*: Frons largely dull to faintly subshiny, brown with moderate investment of gray to yellowish gray microtomentum, anterior margin with some orange coloration; fronto-orbits narrow, gray to whitish gray. Antenna mostly yellow; basal flagellomere tannish yellow apicodorsally; arista with 5 dorsal rays. Face at narrowest point about equal to combined length of pedicel and basal flagellomere; face more densely microtomentose than frons, microtomentum with faintly shiny, mostly gray to whitish gray; parafacial and gena becoming slightly more whitish to silvery white than face; parafacial becoming wider ventrally with moderate ventral dilation; gena moderately high, height less than combined length of pedicel and basal flagellomere; gena-to-eye ratio 0.09–0.12.

*Thorax*: Mesonotum mostly dull to faintly subshiny, moderately densely microtomentose, mostly tan to brown, becoming gray laterally and anteriorly; pleural area mostly gray. Anterior margin of wing lacking spine-like setae; costal vein ratio 0.82–0.86; M vein ratio 0.50–0.55. Legs, including most of coxae, yellow; forefemur lacking a row of 9–10 short, stout setulae along apical half of anteroventral surface; forefemur with a row of 4–5, of moderately well-developed, evenly spaced posteroventral setae on apical half, each seta less than width of femur; tibiae mostly gray, only apices yellowish; basal tarsomeres yellow, apical 1–2 becoming slightly darker, mostly tannish yellow.

**Figures 109–112.**
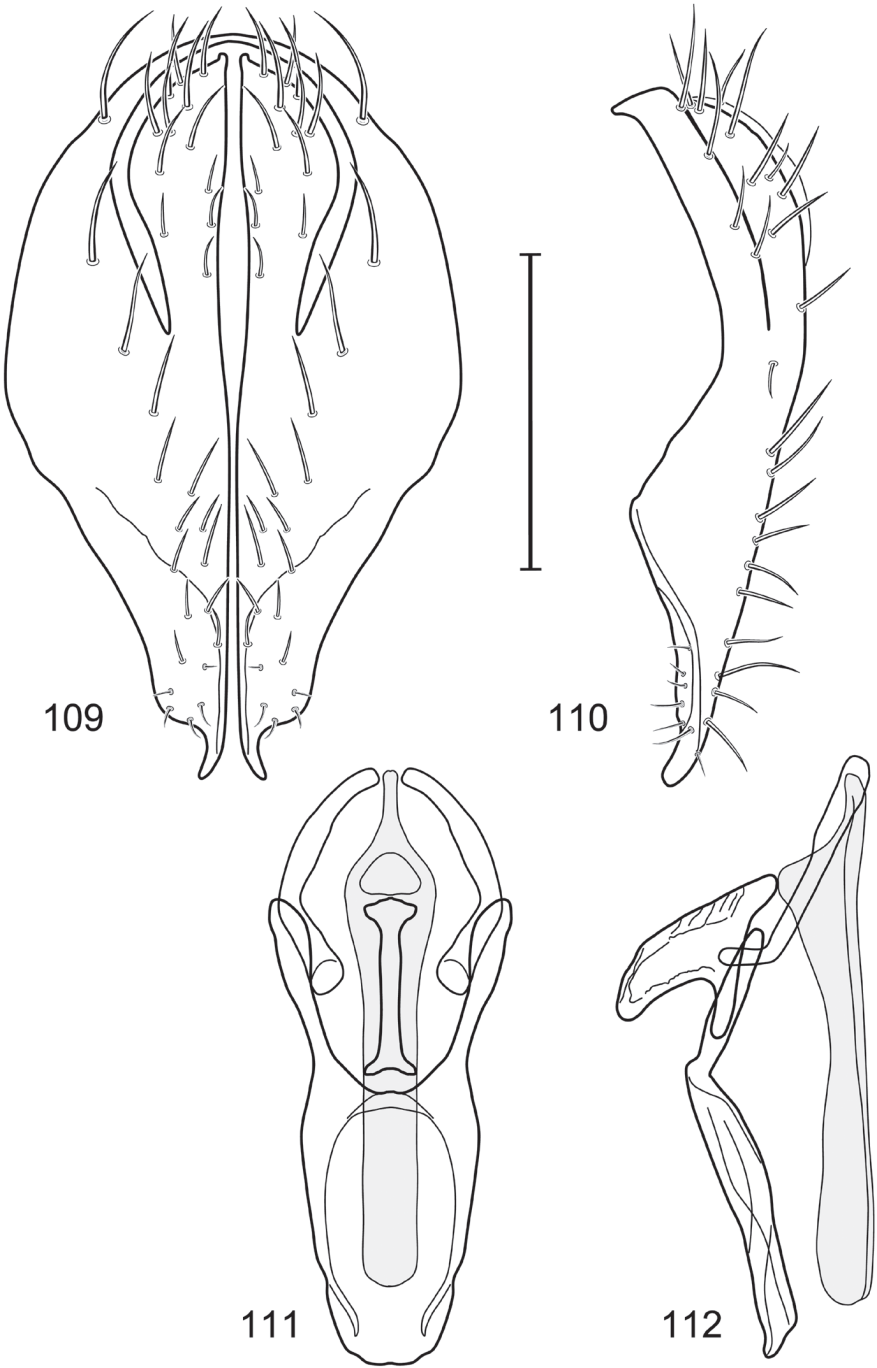
Illustration of *Polytrichophora sturtevantorum* sp. n. (male) (Illinois. Cass: Beardstown) **109** epandrium and cerci, posterior view **110** same, lateral view **111** internal structures of male terminalia (aedeagus [shaded], phallapodeme, gonite, hypandrium), ventral view **112** same, lateral view. Scale bar = 0.1 mm.

*Abdomen*: Tergites dull to subshiny, gray to blackish brown; tergite 5 of male pointed apically, lacking row of 6-10, distinctly larger setae along extreme posterior margin with posterodorsal orientation. Male terminalia (Figs 109-112): Epandrium in posterior view (Fig. 109) generally elliptical, more setulose medially, these becoming shorter ventrally, dorsal margin rounded, both ventral extensions relatively wide until small, digitiform, ventral projections, in lateral view (Fig. 110) with ventral apex narrowly pointed; cercus large, conspicuous; aedeagus simple, lacking distinct basiphallus and distiphallus, in lateral view (Fig. 112) narrowly tubular, straight, anterior margin shallowly curved, posterior margin straight, apex bluntly rounded, in ventral view (Fig. 111) with apical ¾ parallel sided, apex bluntly rounded, base tapered to form a narrow, digitiform base; phallapodeme in lateral view (Fig. 112) roughly triangular with distinct extended keel truncate apically, in ventral view (Fig. 111) with a short bar, T-shaped at both apices; gonite in lateral view (Fig. 112) narrowly elongate, end toward hypandrium distinctly angulate, in ventral view (Fig. 111) angularly arched, slightly wider toward base of hypandrium; hypandrium in lateral view (Fig. 112) elongate, obtusely angulate, in ventral view (Fig. 111) narrowly H-shaped with relatively long anterior and posterior arms.

#### Type material.

The holotype male is labeled “**USA. TN.** Shelby: MeemanShelby St[ate]Park [Mississippi River] 35°20.4'N, 90°2.1'W; 98 m, 10 Jun 2004, W. N. Mathis/USNM ENT 00285964 [plastic bar code label]/HOLOTYPE ♂ *Polytrichophora sturtevantorum* Mathis & Zatwarnicki, USNM [red].” The holotype is double mounted (minuten pin in a block of plastic), is in excellent condition, and is deposited in the USNM. Ten paratypes (7♂, 3♀; USNM) bear the same label data as the holotype.

#### Type locality.

United States. Tennessee. Shelby: Meeman Shelby State Park (Mississippi River; 35°20.4'N, 90°2.1'W; 98 m).

#### Other specimens examined.

Nearctic. UNITED STATES. ILLINOIS. **Adams:** Quincy (Mississippi River; 39°56.1'N, 91°25.1'W), 3 Sep 1995, J. F. Edmiston (5♂, 4♀; USNM). **Cass:** Beardstown (40°01.1'N, 90°25.5'W), 18 Sep 1954, A. H. Sturtevant (3♂; USNM). **Cook:** Chicago (41°52.7'N, 87°37.8'W) (1♀; ANSP).

IOWA. **Allamakee:** Waterville (5 km ESE; 43°11'N, 91°14.1'W), 3 Aug 1960, D. L. Deonier (1♂; USNM). **Boone:** Fraser Milldam (42°07.6'N, 93°58.6'W), 4 Jul 1960, D. L. Deonier (4♂, 5♀; USNM); Ledges State Park (Des Moines River; 41°59.5'N, 93°53.5'W), 14 Jun–16 Aug 1960, D. L. Deonier (16♂, 10♀; USNM). **Hamilton:** Goose Lake (42°19.9'N, 93°37.4'W), 22 Jul-6 Aug 1960, D. L. Deonier (3♀; USNM). **Louisa:** Wapello (6.5 km NE; 41°13.8'N, 91°07.2'W), 9 Aug 1960, D. L. Deonier (31♂, 10♀; USNM). **Monona:** Lewis and Clark State Park (42°02'N, 96°10'W), 6 Jun 1960, D. L. Deonier (2♂; USNM). **Story:** Ames (42°02.1'N, 93°37.2'W), 12 May–17 Jul 1960, D. L. Deonier (11♂, 15♀; USNM). **Warren:** Banner Mine Area (41°26.4'N, 93°33.8'W), 7 Aug 1960, D. L. Deonier (2♂, 1♀; USNM).

KANSAS. **Riley:** Manhattan (39°11'N, 96°34'W), 6 Jun 1932, C. W. Sabrosky (1♂; ANSP).

LOUISIANA. **Sabine:** Many, 6 Jul 1933, R. Nabors, C. W. Sabrosky (1♂; ANSP).

MISSISSIPPI. **Oktbbeha:** Agriculture College (State College; 33°27.1'N, 88°47'W), 30 Oct 1922 (1♂; ANSP). **Washington:** Leroy Percy State Park (W Hollandale; 33°09.8'N, 90°56.2'W; 63 m), 9 Jun 2004, W. N. Mathis (20♂, 1♀; USNM).

MISSOURI. **Jackson:** Atherton (39°11.2'N, 94°18.3'W) (1♂; ANSP). **Laclede:** Lebanon (37°40.8'N, 92°39.8'W), 19 Sep 1954, A. H. Sturtevant (1♂; USNM). **Lawrence:** La Russell (37°11.5'N, 94°01.1'W), 6 Sep 1961, D. L. Deonier (3♂, 1♀; USNM). **St. Louis:** St. Louis (38°37.6'N, 90°12'W), 25 Jun 1951, H. Stalker (1♂; USNM).

MONTANA. **Gallatin:** Three Forks (44°53.5'N, 111°53.1'W), 3 Aug 1918, A. L. Melander (1♀; ANSP).

OHIO. **Ashtabula:** Pymatuning Lake State Park (41°39.4'N, 80°27.7'W), 13 Sep 1976, B. A. Steinly (4♂, 4♀; USNM). **Claremont:** Stonelick (39°13.6'N, 84°03.5'W), 1 Aug 1974, J. Regensberg (2♂, 3♀; USNM). **Hocking:** Logan (39°32'N, 82°26.9'W), 18 May 2003, D. and W. N. Mathis (1♂; USNM). **Morgan:** Burr Oak State Park (39°33.3'N, 82°03.9'W), 26 Aug 1974, J. Regensberg (7♂, 9♀; USNM). **Preble:** Seven-mile Creek at Eaton (39°43.8'N, 84°35.9'W), 24 Jul 1974, J. Regensberg (2♂; USNM). **Van Wert:** Little Auglaize River (40°46.7'N, 84°30.3'W), 18 Aug 1976, B. A. Steinly (15♂, 17♀; USNM).

TEXAS. **Galveston:** Galveston Island (29°10'N, 94°05'W), 14 May 1993, D. and W. N. Mathis (1♂; USNM). **Jasper:** Boykin Springs (31°05'N, 94°17'W), 15 May 1993, D. and W. N. Mathis (4♂; USNM).

WISCONSIN. **Washburn:** (46°40.4'N, 90°53.7'W), 10 Jun-10 Aug 1951, 1953, R. H. Jones (8♂, 5♀; USNM).

#### Distribution

**Figure 113.**
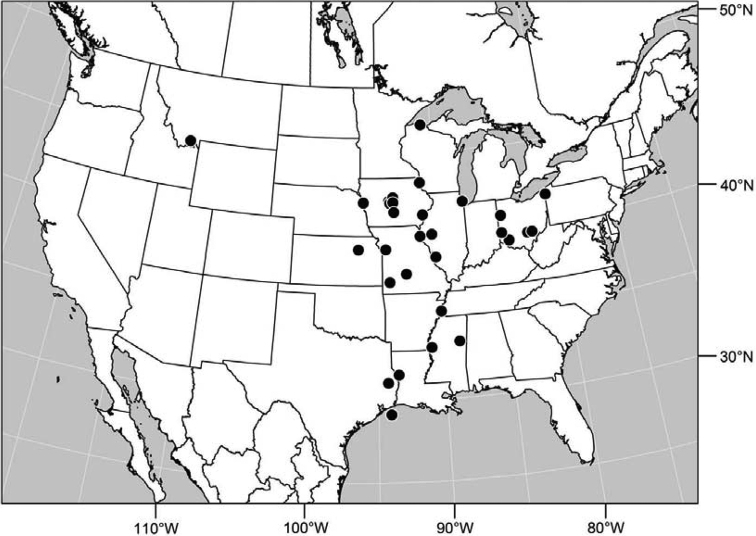
Distribution of *Polytrichophora sturtevantorum* sp. n.

(Fig. 113). Nearctic: United States (Illinois, Iowa, Kansas, Louisiana, Missippi, Missouri, Montana, Ohio, Tennessee, Texas, Wisconsin).

#### Etymology.

The species epithet, *sturtevantorum*, is a plural genitive patronym to recognize and honor a father and son. The father, Alfred H. Sturtevant (1891-1970), was a world-renowned geneticist who also conducted taxonomic research on shore flies (Sturtevant and Wheeler 1954) and was an indefatigable collector of acalyptrate Diptera. Most of A. H. Sturtevant'S shore-fly collection is deposited in the USNM, where his son, William C. Sturtevant (1926-2007; see Merrill and Goddard 2002 for biographical information), was a colleague and distinguished scholar in the Department of Anthropology (USNM).

#### Remarks.

Among species of the *orbitalis* group, this species is distinguished by characters of the male terminalia as follows: Area between anterior arms of the hypandrium is partially sclerotized; the ventral epandrial processes are relatively wide until small, digitiform, ventral projections (Fig. 109); the posteroventral edge of the epandrium in lateral view is evenly curved; and the taper toward the apex is more gradual and acute (Fig. 110).

##### The reginae Group

**Species included:** *Polytrichophora prolata* sp. n. and *Polytrichophora reginae* Mathis.

#### Discussion.

Externally, this species group is recognized by having male tergite five bearing a distinct row of six to ten longer setae along the extreme posterior margin. These setae have a posterodorsal orientation.

The monophyly of this group is questionable. Although the male tergite five is somewhat similar, structures of the male terminalia are remarkably different and may indicate that the two included species are not that closely related. In *Polytrichophora reginae*, the ventral portion of the epandrium in posterior view is pointedly rounded, which is unique within the genus. In *Polytrichophora prolata*, the ventral portion of the epandrium is as two, elongated, and narrow processes, more typical of many other congeners.

### Polytrichophora prolata sp. n.

urn:lsid:zoobank.org:act:364101EE-9D9B-4E9E-AC13-8D6A9EE1E9B8

http://species-id.net/wiki/Polytrichophora_prolata

Figs 114 118

#### Diagnosis.

This species is distinguished from congeners by the following combination of characters: Moderately small shore flies, body robust, body length 2.55–2.85 mm.

*Head*: Frons largely dull, gray to dark brown with moderate investment of gray to yellowish gray microtomentum, anterior margin with some yellowish to orange coloration; fronto-orbits moderately narrow, whitish gray to yellowish white. Antenna mostly yellow; dorsum of pedicel blackish; basal flagellomere tannish yellow apicodorsally; arista with 5 dorsal rays. Face relatively narrow, at narrowest point width less than combined length of pedicel and basal flagellomere; face more densely microtomentose than frons, microtomentum with shiny luster, midfacies mostly grayish tan to brown; parafacial yellowish gold, becoming more whitish gray; gena becoming slightly more whitish to silvery white than face; parafacial becoming slightly wider ventrally with little ventral dilation; gena moderately short, height less than height of basal flagellomere; gena-to-eye ratio 0.09–0.10.

*Thorax*: Mesonotum mostly dull to faintly subshiny, moderately densely microtomentose, mostly tan to brown, becoming gray laterally and anteriorly; pleural area mostly gray. Anterior margin of wing lacking spine-like setae; costal vein ratio 0.54–0.59; M vein ratio 0.35–0.37. Legs, including most of coxae, yellow; forefemur lacking a row of 9–10 short, stout setulae along apical half of anteroventral surface; forefemur with a row of 4–5, of moderately well-developed, evenly spaced posteroventral setae on apical half, each seta less than width of femur; tibiae mostly gray, only apices yellowish; basal tarsomeres yellow, apical 1–2 becoming slightly darker, mostly tannish yellow.

**Figures 114–117.**
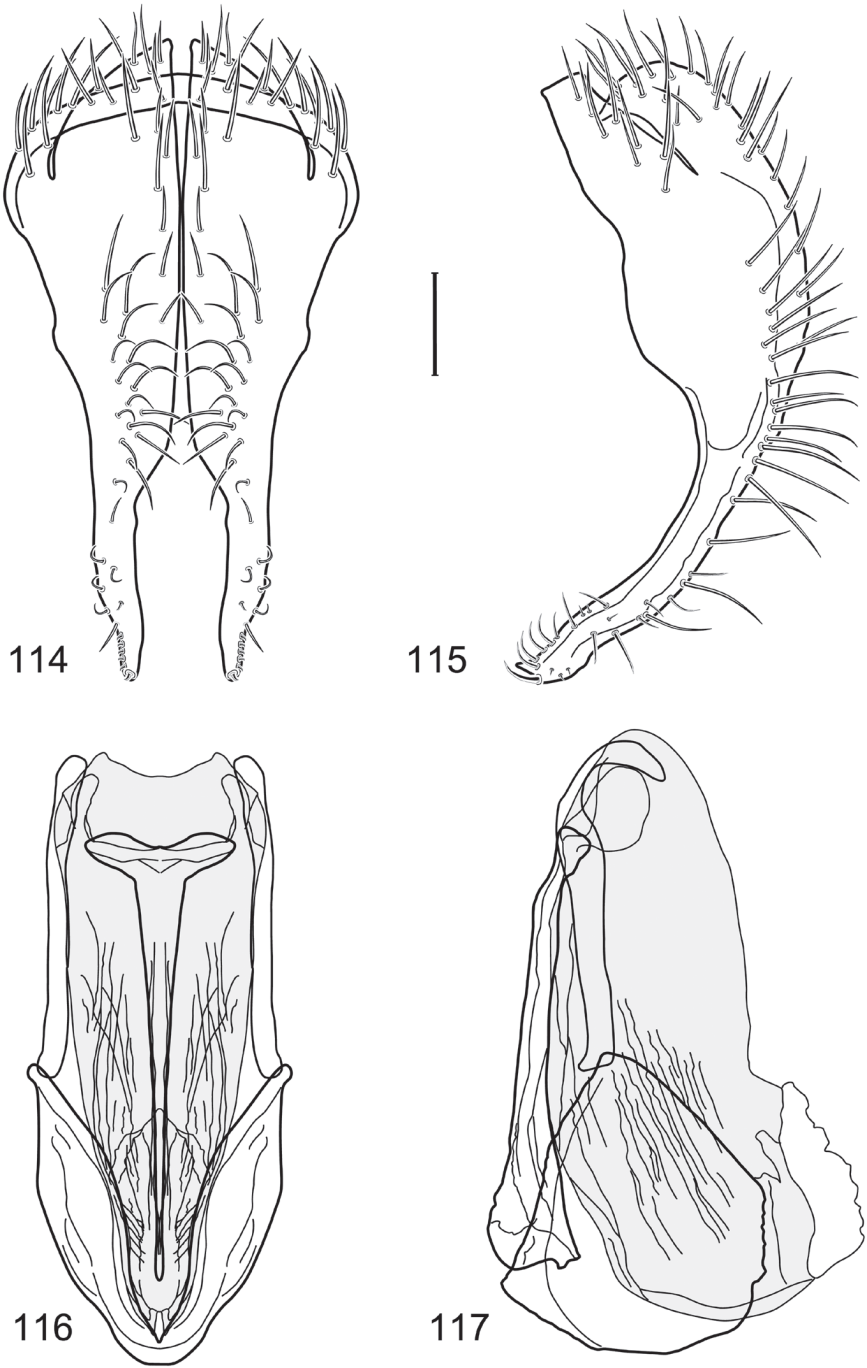
Illustration of *Polytrichophora prolata* sp. n. (male) (Belize. Stann Creek: Cockscomb Basin Wildlife Sanctuary) **114** epandrium and cerci, posterior view **115** same, lateral view **116** internal structures of male terminalia (aedeagus [shaded], phallapodeme, gonite, hypandrium), ventral view **117** same, lateral view. Scale bar = 0.1 mm.

*Abdomen*: Abdomen, including tergite 5 of male, entirely black, shiny dorsally, slightly grayish and very sparsely microtomentose ventrally; sternites 4-5 of male partially setulose but not as dense patches. Male terminalia (Figs 114-117): Epandrium in posterior view (Fig. 114) narrowly connected dorsally, dorsum bluntly rounded, each lateral half very narrowly triangular, tapered ventrally, ventral extensions well separated on ventral 1/3, but extended parallel to each other, each tapered to narrowly rounded point, extensions bearing short setulae, in lateral view (Fig. 115) as a bent turkey leg, ventral extension moderately well developed, mostly parallel sided, subapically shallowly sinuous, apex pointed; cerci evident in lateral view, moderately well developed; aedeagus in lateral view (Fig. 117) very robustly developed, relatively very broad, length slightly more than twice length, broadly truncate apically, in ventral view (Fig. 116) wedge-like, robust, basal half with lateral margins parallel sided, tapered to point on apical half; phallapodeme in lateral view (Fig. 117) elongate, clavate, keel forming club, in ventral view (Fig. 116) narrowly T-shaped with short, robust bar basally; gonite in lateral view (Fig. 117) elongate, rod-like on most of length, end toward aedeagal base distinctly curved, in ventral view (Fig. 116) narrowly bar-like, parallel sided, rounded at each end; hypandrium in lateral view (Fig. 117) irregularly quadrate, somewhat pointed toward gonite, in ventral view (Fig. 116) robustly and deeply V-shaped.

**Type material.** The holotype male of *Polytrichophora prolata* is labeled “**BELIZE.** Stann Creek District: Cockscomb Basin W[i]ldlife Sanct[uary]. (16°47'N, 88°30'W)[,] 5-6Apr1993, W.Mathis/USNM ENT 00285966 [plastic bar code label]/HOLOTYPE ♂ *Polytrichophora prolata* Mathis & Zatwarnicki, USNM [red].” The holotype is double mounted (minuten pin in a block of plastic), is in excellent condition, and is deposited in the USNM. Nineteen paratypes (10♂, 9♀; USNM) bear the same label data as the holotype.

#### Type locality.

Belize. Stann Creek: Cockscomb Basin Wildlife Sanctuary (16°45'N, 88°30'W).

#### Other specimens examined.

Neotropical. BRAZIL. **Paraná.** Morretes (25°28'S, 48°59.1'W), 29 Aug 2000, D. and W. N. Mathis (1♀; USNM).

ECUADOR. **Puerto Orellana:** Rio Tiputini Biodiversity Station (0°38.2'S, 76°8.9'W), 12-26 Aug 1999, A. Baptista, M. Kotrba, W. N. Mathis (4♂, 3♀; USNM).

PERU. **Madre de Dios:** Rio Manu, Pakitza (11°56.6'S, 71°16.9'W; 250 m), 9–23 Sep 1988, W. N. Mathis (2♂, 1♀; USNM).

#### Distribution

**Figure 118.**
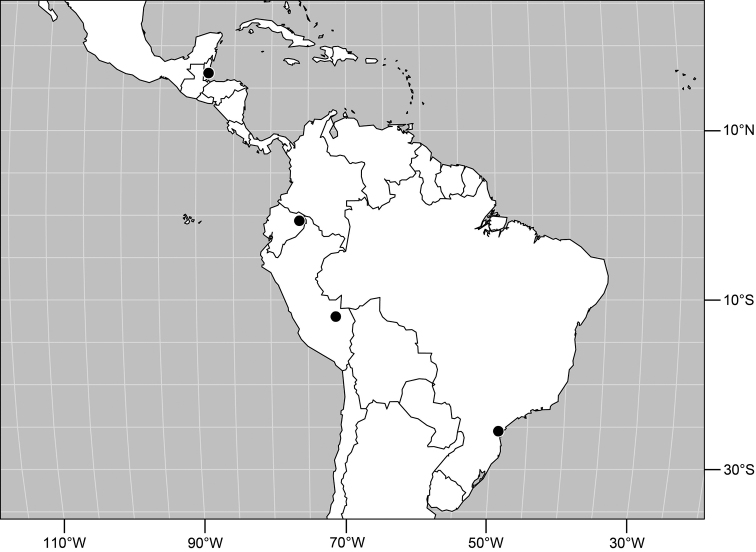
Distribution of *Polytrichophora prolata* sp. n.

(Fig. 118). Neotropical: Belize, Brazil (Paraná), Ecuador (Puerto Orellana), Peru (Madre de Dios).

#### Etymology.

The species epithet, *prolata*, is of Latin derivation and refers to the prolongated, ventral extensions of the epandrium.

#### Remarks.

This species is distinguished from *Polytrichophora reginae* in being larger (body length of 2.80 mm or longer), having male tergites one through five entirely black and somewhat subshiny (not bicolored as in *Polytrichophora reginae*), and in having male sternites four and five more uniformly setulose (not in dense patches as in *Polytrichophora reginae*). In addition, the ventral portion of the epandrium (Figs 114–115) is as two narrow, elongated processes (pointedly rounded in *Polytrichophora reginae*).

### Polytrichophora reginae

Mathis

http://species-id.net/wiki/Polytrichophora_reginae

Figs 119 123

Polytrichophora reginae Mathis 1997: 39.

#### Diagnosis.

This species is distinguished from congeners by the following combination of characters: Small to moderately small shore flies, body length 1.70–2.40 mm.

*Head*: Frons dull, heavily microtomentose, 2-toned, mostly tan with some faint golden reflections on posterior 2/3, anterior 1/3 (from level of fronto-orbital setae anteriad), gray. Antenna mostly yellow to yellowish orange, anterior portion of pedicel and basodorsal area of basal flagellomere with some blackish coloration; arista with 5 dorsal rays. Face at narrowest point about equal to combined length of pedicel and basal flagellomere; face densely microtomentose, microtomentum with shiny to pearly luster, mostly white but with considerable gold coloration in antennal grooves and extended laterally onto parafacial; parafacial not markedly differing from middle of face in color, more golden dorsally, becoming whiter ventrally; parafacial with slight to considerable ventral dilation; parafacial 2–3 times wider ventrally than dorsally; gena relatively short, less than 1/4 eye height; gena-to-eye ratio 0.12–0.14.

*Thorax*: Mesonotum mostly dull, densely microtomentose, concolorous with posterior 2/3 of frons; pleural area blackish gray. Anterior margin of wing lacking spine-like setae; costal vein ratio 0.36–0.43; M vein ratio 0.56–0.62. Forefemur lacking row of 9–10 short, stout setae along apical half of anteroventral surface; forefemur with a row of 7–9, well-developed, evenly spaced posteroventral setae, each equal to width of femur; tibiae mostly yellowish; basal tarsomeres yellow, apical 1–2 brown.

**Figures 119–122.**
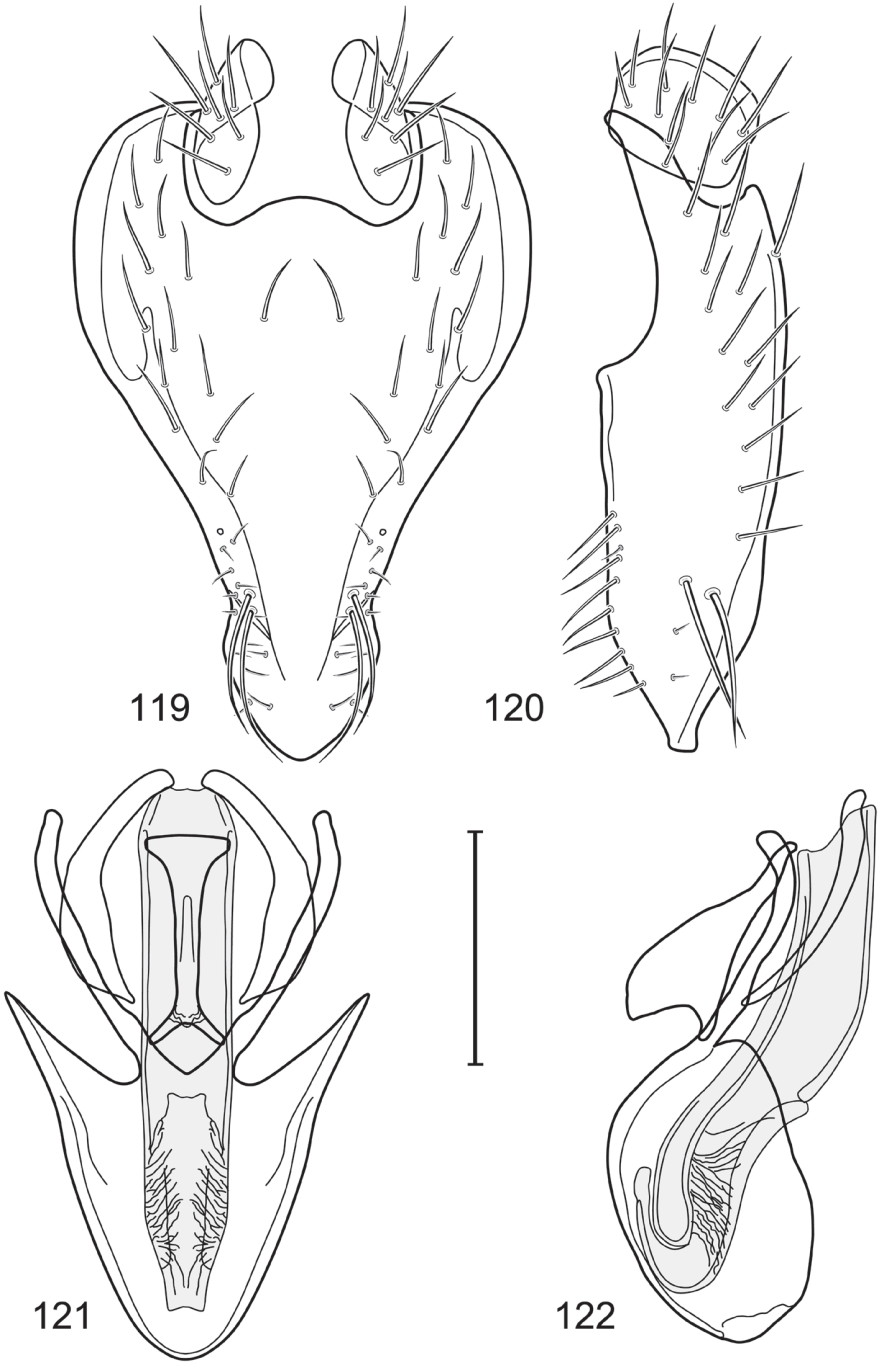
Illustration of *Polytrichophora reginae* Mathis (male) (Ecuador. Loja: Catamayo) **119** epandrium and cerci, posterior view **120** same, lateral view **121** internal structures of male terminalia (aedeagus [shaded], phallapodeme, gonite, hypandrium), ventral view **122** same, lateral view. Scale bar = 0.1 mm.

*Abdomen*: Tergites 1–4 bicolored, subshiny to dull, dark gray medially, gray lateroventrally; tergites 3–4 of male subequal in length, tergite 4 not unusually longer, tergite 3 not produced ventrolaterally; dorsum of tergite 5 of male not darker than preceding tergites and usually with distinct, semicircular, grayish areas laterally; tergite 5 of male in dorsal view blunt, truncate, bearing row of 6-10, distinctly larger setae along extreme posterior margin with posterodorsal orientation; tergite 5 of male also gray on posterior 1/2-1/3; sternites 3-4 of male with dense row of setulae along posterior margin; sternite 5 as 2 subtriangular sternites. Male terminalia (Fig s 119–122): Epandrium not connected dorsally above cerci, in posterior view (Fig. 119) tapered ventrally to an acutely pointed apex under which lies cordate-shaped, laterally flanged anterior margin (Fig. 119); epandrium in lateral view (Fig. 120) slightly curved anteroventrally, pointed apically, and with subapical ledge that also ends ventrally in a point, bearing large, subapical setae laterally (Figs 119–120); gonite in lateral view rod-like, shallowly curved (Fig. 122); aedeagus in lateral view (Fig. 122) shallowly sinuous, bifurcate apically, anterior process sclerotized, posterior process longer, wider, membranous, apex complicated with gill-like folds laterally, apex slender, recurved anterodorsally giving appearance of rounded apex; phallapodeme with extended flange fan-like (Fig. 122); hypandrium in lateral view sinuous (Fig. 122), in posterior view deeply Y-shaped.

#### Type material.

The holotype male is labeled “BELIZE.StannCreek Dist: Wee Wee Cay, 6-9 November 1987[,] W.N. and D. Mathis/HOLOTYPE Polytrichophora reginae ♂ W. N.Mathis USNM [species name and gender handwritten, red].” The holotype is double mounted (minuten pin in a block of plastic), is in excellent condition, and is deposited in the USNM. The allotype female and 15 paratypes (3♂, 12♀; USNM) bear the same label data as the holotype. A female paratype is from the same district in Belize as follows: Twin Cays (Aanderaa Flats), Nov 1987, W. N. and D. Mathis (1♀; USNM). Other paratypes are as follows: PERU. **Cuzco:** Paucartambo, Atalaya (Río Alto Madre de Dios; 12°53.1'S, 71°21.6'W; 600 m), 4 Sep 1988, W. N. Mathis (2♂; USNM). **Huánuco:** Huánaco (10 km N), Rio Huallaga, 4 Feb 1984, W. N. Mathis (2♂, 1♀; USNM). **Madre de Dios:** Río Manu, Cocha Salvador (11°59.9'S, 71°13.9'W 300m), 14 Sep 1988, W. N. Mathis (1♂, 1♀; USNM); Diamante (Río Alto Madre de Dios; 12°19.9'S, 70°57.5'W; 400 m), 7 Sep 1988, W. N. Mathis (2♂, 1♀; USNM); Quebrada Romero (near; Rio Manu; 12°07'S, 70°58'W), 8 Sep 1988, W. N. Mathis (1♀; USNM); Río Manu, Pakitza (11°56.6'S, 71°16.9'W; 250 m), 9–23 Sep 1988, W. N. Mathis (4♂, 4♀; USNM).

West Indies. ST. LUCIA. Marquis (1.5 km S, 14°01'N, 60°55'W), 17 Jun 1992, W. N. and D. Mathis (2♂, 3♀; USNM).

#### Type locality.

Belize. Stann Creek: Wee Wee Cay (16°45.9'N, 88°08.6'W).

#### Other specimens examined.

Neotropical. ARGENTINA. **Tucumán:** Río Colorado (27°09'S, 65°21.4'W), 17 Jan 1948, R. Golbach (1♀; USNM).

BOLIVIA. **El Beni:** Huachi, Sep, W.M. Mann (1♂, 1♀; USNM); Rosario Lake, Rogagua, 28 Oct-9 Nov 1921, W.M. Mann (1♀; USNM). **La Paz:** Guanay (3 km E; 15°30.2'S, 67°52.3'W; 500 m), 14 Mar 2001, W. N. Mathis (15♂, 8♀; USNM); Mapiri (Rio Mapiri; 15°18.6'S, 68°13'W; 720 m), 17 Mar 2001, W. N. Mathis (1♂, 2♀; USNM); Puente Villa (2 km E; 16°24'S, 67°38'W; 1960 m), 11 Mar 2001, W. N. Mathis (2♂; USNM); San Pedro (3 km NE; 16°S, 67°35.3'W; 780 m), 12 Mar 2001, W. N. Mathis (5♂, 7♀; USNM); Tajlihui (15°40.8'S, 67°41.7'W; 590 m), 12 Mar 2001, W. N. Mathis (1♂; USNM).

BRAZIL. **Paraná.** Morretes (25°28'S, 48°59.1'W), 29 Aug 2000, D. and W. N. Mathis (1♂; USNM); Parque Igauçu (25°33.4'S, 49°13.6'W; 880 m), 12 Apr 2010, D. and W. N. Mathis (4♀; DZUP, USNM). **São Paulo.** Ubatuba, Praia do Estaleiro (23°20.5'S, 44°53'W; beach), 29 Mar 2010, D. and W. N. Mathis (2♀; DZUP, USNM); Ubatuba, Praia Puruba (23°21'S, 44°55.6'W; beach), 29 Mar 2010, D. and W. N. Mathis (5♂; USNM).

COSTA RICA. **Cartago:** La Suiza (9°51.5'N, 83°37.5'W), 28 Jun 2001, W. N. Mathis (1♂; USNM). **Guanacaste:** Liberia (10°37.8'N, 85°26.4'W), 27 Mar 1987, J. M. Hill (1♀; USNM); Santa Cruz (14 km S; 10°10.4'N, 85°35.7'W; 180 m), 23 Jun 2001, D. and W. N. Mathis (8♂, 2♀; USNM). **Heredia:** Santo Domingo (Parque INBIO; 9°59'N, 85°06'W), 23 Jun 2001, D. and W. N. Mathis (1♀; USNM).

DOMINICAN REPUBLIC. **Barahona:** Baoruco (18°04.6'N, 71°05.5'W; beach and river), 19 May 1998, D. and W. N. Mathis (1♀; USNM). **Hato Mayor:** Hato Mayor (5.5 km E; 18°46.4'N, 69°12.5'W), 26 May 1998, D. and W. N. Mathis (1♂; USNM). **La Vega:** Constanza (ca. 14 km SE; 18°51.4'N, 70°41.2'W; 1505 m), 15 May 1998, D. and W. N. Mathis (1♂; USNM); El Rio (9.5 km E; 19°0.9'N, 70°33.5'W; 980 m), 6 May 1995, W. N. Mathis (1♂; USNM); Jarabacoa (1-2 km S; 19°06.9'N, 70°37'W; 520 m), 8-21 May 1995, 1998, D. and W. N. Mathis (15♂, 15♀; USNM); Rio Camu (3.5 km NW La Vega; 19°13.7'N, 70°35.2'W; 100 m), 10 May 1995, 1998, D. and W. N. Mathis (16♂, 2♀; USNM); Salto Baiguate (near Jarabacoa; 19°05.5'N, 70°36.9'W; 570 m), 9-16 May 1995, 1998, D. and W. N. Mathis (2♂, 5♀; USNM). **Peravia:** Rio Ocoa (San José Ocoa; 18°31.7'N, 70°30.4'W), 21 May 1998, D. and W. N. Mathis (1♀; USNM). **Puerto Plata:** Rio Camu (14 km E Puerto Plata; 19°41.9'N, 70°37.5'W), 23 May 1998, D. and W. N. Mathis (10♂; USNM).

ECUADOR. **El Oro:** Pasaje (6 km E; 3°18.1'S, 79°47.1'W), 13 Jan 1978, W. N. Mathis (1♂, 6♀; USNM). **Guayas:** Gala (3°0.7'S, 79°44.3'W), Dec 1955, J. R. Levi-Castillo (5♀; USNM). **Loja:** Catamayo (3°59'S, 79°21'W), Dec 1955, J. R. Levi-Castillo (4♂, 1♀; USNM). **Manabí:** Pichincha (0°08.8'S, 78°28.5'W), Aug 1955, J. R. Levi-Castillo (6♀; USNM). **Puerto Orellana:** Rio Tiputini Biodiversity Station (0°38.2'S, 76°8.9'W), 12–26 Aug 1999, A. Baptista, M. Kotrba, W. N. Mathis (10♂, 3♀; USNM).

EL SALVADOR. Acajutla (13°35.4'N, 89°49.8'W), Dec 1953, W. B. Heed (2♀; USNM).

GRENADA. **St. Andrew:** Balthazar (12°07.7'N, 61°39.3'W), 19 Sep 1996, W. N. Mathis (1♂; USNM). **St. John:** Palmiste Lake (12°08.3'N, 61°44'W), 19 Sep 1996, W. N. Mathis (1♂; USNM).

GUYANA. Kaieteur Falls (5°10.5'N, 59°28.9'W), 21-24 Aug 1997, W. N. Mathis (1♀; USNM); Karanambo (Rupununi River; 3°45.1'N, 59°18.6'W; 85m), 1-3 Dec 2010, W. N. Mathis (2♂, 1♀; USNM); Pirara Ranch and River (03°32.1'N, 59°40.5'W), 24-25 Apr 1995, W. N. Mathis (1♀; USNM); Takutu Mountains (6°15'N, 59°05'W), 17 Dec 1983, P. J. Spangler, W. E. Steiner (1♀; USNM).

HONDURAS. **Cortés:** Omoa (15°47.8'N, 87°58.4'W), 26 Sep 1995, D. and W. N. Mathis (1♀; USNM); San Pedro Sula (8 km S; 15°25.7'N, 88°01.4'W), 25-26 Sep 1995, D. and W. N. Mathis (3♂, 1♀; USNM).

JAMAICA. **Clanendon:** Grantham (18°09.3'N, 77°23.8'W; 340 m), 16 Apr 2000, W. N. Mathis (1♀; USNM). **Manchester:** near Mandeville (18°03.5'N, 77°31.9'W), 15-18 Apr 2000, W. N. Mathis (1♂; USNM). **Portland:** Berridale (18°06.5'N, 76°20'W), Rio Grande River, 25 Apr 2000, W. N. Mathis (2♀; USNM). **St. Andrew:** Mavis Bank (4.3 km SE; 18°01.4'N, 76°38.1'W; 480 m); Yallahs River, 22-23 Apr 2000, W. N. Mathis (1♂, 1♀; USNM); Wag Water River, 25 Feb 1969, W. W. Wirth (1♀; USNM)l. **St. Thomas:** Mt. Lebanus (17°58.2'N, 76°32.7'W), 16 May 1996, D. and W. N. Mathis, H. B. Williams (1♀; USNM).

MEXICO. **Chiapas:** Finca Esperanza (26°38'N, 103°28'W), 26 Aug 1956, R. Nettel F. (1♂; USNM); Puente Mezcalapa (17°21.9'N, 93°23.5'W; light trap), 22 May 1964, F. S. Blanton (7♂, 5♀; USNM). **San Luis Potosi:** Tamazunchale (21°15.3'N, 98°47.3'W), 23 Nov 1946, F. E. Skinner (1♂, 1♀; USNM). **Veracruz-Llave:** Fortín de las Flores (18°54'N, 97°0'W; light trap), Jun 1964, F. S. Blanton (3♂; USNM).

NICARAGUA. **León:** La Cruz de la India (5 km SW, on highway 26; 12°43.2'N, 86°19.8'W; 215 m), 22 Jun 2007, W. E. Woodley (2♂, 5♀; USNM).

PANAMA. **Panama:** Canal Zone, Gamboa, Pipeline Road (9°07'N, 79°42'W), Jul 1967, W. W. Wirth (1♂; USNM); Canal Zone, Pedro Miguel (9°01.1'N, 79°36.7'W), R. C. Shannon (1♂; USNM); Tocúmen (9°05.2'N, 79°23.1'W), 13 Feb 1953, F. S. Blanton (1♂; USNM).

PERU. **Huánuco:** Tingo Maria (1 km S; 9°18.5'S, 76°0.3'W), 6 Feb 1984, W. N. Mathis (3♀; USNM). **Madre de Dios:** Río Manu, Erika (near Salvación; 12°50.7'S, 71°23.3'W; 550 m), 5-6 Sep 1988, W. N. Mathis (1♂, 2♀; USNM).

PUERTO RICO. Adjuntas (18°09.8'N, 66°43.2'W), 22 Sep 1995, D. and W. N. Mathis (4♂, 1♀; USNM). Maricao (18°11.1'N, 66°58.9'W), 21 Sep 1995, D. and W. N. Mathis (4♂, 1♀; USNM). Maricao (4 km WNW; 18°10.7'N, 66°59.6'W), 21 Sep 1995, D. and W. N. Mathis (1♂; USNM). Playa de Guayanilla (18°0.4'N, 66°46.1'W), 19 Sep 1995, D. and W. N. Mathis (1♂; USNM). Rio Hoconuco (18°7.6'N, 67°2.6'W), 20 Sep 1995, D. and W. N. Mathis (3♂, 1♀; USNM).

ST. VINCENT. **St. David:** Richmond Beach (13°18'N, 61°14'W), 28 Mar 1989, W. N. Mathis (1♂; USNM).

TRINIDAD AND TOBAGO. Trinidad. **St. Andrew:** Valencia (1 km W; 10°39'N, 61°13'W), Aripo River, 20 Jun 1993, W. N. Mathis (4♂, 2♀; USNM). **St. George:** Arima (8 km N; 10°41'N, 61°18'W), Verdant Vale, 19 Jun 1993, W. N. Mathis (2♂; USNM). Tobago. **St. John:** Bloody Bay River (11°18'N, 60°38'W), 14 Jun 1993, W. N. Mathis (1♂, 3♀; USNM); Charlotteville (5 km S; 11°18.9'N, 60°34.5'W), Hermitage River and beach, 10 Jun 1993, W. N. Mathis (2♀; USNM); Kings Bay Reservoir (11°17'N, 60°33'W), 15 Jun 1993, W. N. Mathis (5♂, 5♀; USNM); Parlatuvier (creek; 11°17.9'N, 60°35'W), 20 Apr 1994, W. N. Mathis (1♂; USNM); Speyside (Doctor River; 1 km NW; 11°18'N, 60°32'W), 12-13 Jun 1993, W. N. Mathis (1♂, 2♀; USNM). **St. Paul:** Delaford, Kings Bay (11°16'N, 60°32.8'W), 13 Jun 1993, W. N. Mathis (1♀; USNM).

#### Distribution

**Figure 123.**
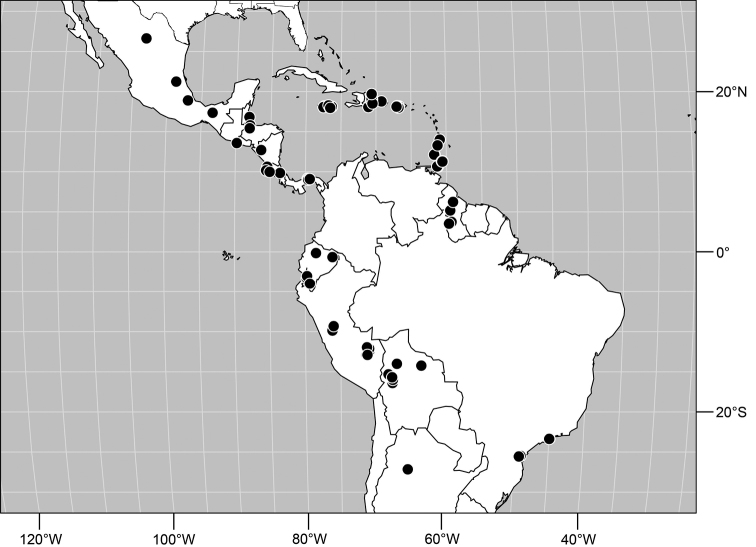
Distribution of *Polytrichophora reginae* Mathis.

(Fig. 123). Neotropical: Argentina (Tucumán), Belize, Bolivia (El Beni, La Paz), Brazil (Paraná, São Paulo), Costa Rica (Cartago, Guanacaste, Heredia), Ecuador (El Oro, Guayas, Loja, Manabí, Puerto Orellana), El Salvador, Guyana, Honduras, Mexico (Chiapas, San Luis Potosi, Veracruz-Llave), Nicaragua, Panama, Peru (Huánuco, Madre de Dios), Trinidad and Tobago, West Indies (Dominican Republic, Grenada, Jamaica, Puerto Rico, St. Lucia, St. Vincent).

#### Remarks.

This species is distinguished from *Polytrichophora prolata* in having a smaller body (body length of 2.40 mm or smaller). In addition, male tergites one through four are bicolored (mostly blackish brown dorsally in contrast with the lateroventral surface being gray), male tergite five is truncate and is gray on the posterior one-third, and male sternites three and four have a dense patch of setulae along the posterior margin. Structures of the male terminalia (Figs 119–122) are unique within the genus in having the ventral margin pointedly rounded, without two apical processes.

## Supplementary Material

XML Treatment for Discocerinini

XML Treatment for Facitrichophora

XML Treatment for Facitrichophora atrella

XML Treatment for Facitrichophora carvalhorum

XML Treatment for Facitrichophora manza

XML Treatment for Facitrichophora panama

XML Treatment for Polytrichophora

XML Treatment for Polytrichophora adarca

XML Treatment for Polytrichophora agens

XML Treatment for Polytrichophora arnaudorum

XML Treatment for Polytrichophora barba

XML Treatment for Polytrichophora conciliata

XML Treatment for Polytrichophora flavella

XML Treatment for Polytrichophora marinoniorum

XML Treatment for Polytrichophora rostra

XML Treatment for Polytrichophora setulosa

XML Treatment for Polytrichophora sinuosa

XML Treatment for Polytrichophora desmata

XML Treatment for Polytrichophora pulchra

XML Treatment for Polytrichophora mimbres

XML Treatment for Polytrichophora orbitalis

XML Treatment for Polytrichophora salix

XML Treatment for Polytrichophora setigera

XML Treatment for Polytrichophora sturtevantorum

XML Treatment for Polytrichophora prolata

XML Treatment for Polytrichophora reginae
